# Differential functions of RhoGDIβ in malignant transformation and progression of urothelial cell following N-butyl-N-(4-hydmoxybutyl) nitrosamine exposure

**DOI:** 10.1186/s12915-023-01683-2

**Published:** 2023-08-28

**Authors:** Xiaohui Hua, Ronghao Zou, Xiaoyue Bai, Yuyao Yang, Juan Lu, Chuanshu Huang

**Affiliations:** 1 Department of Occupational Health and Environmental Health, School of Public Health, Anhui Medical University, Hefei, Anhui 230032 People’s Republic of China; 2 Oujiang Laboratory (Zhejiang Lab for Regenerative Medicine, Vision and Brain Health), Key Laboratory of Laboratory Medicine, Ministry of Education, School of Laboratory Medicine and Life Sciences, Wenzhou Medical University, Wenzhou, 325035 Zhejiang China

**Keywords:** RhoGDIβ, Tumor promotion/progression, Cancer invasion, Bladder carcinogenesis

## Abstract

**Background:**

Functional role of Rho GDP-dissociation inhibitor beta (RhoGDIβ) in tumor biology appears to be contradictory across various studies. Thus, the exploration of the molecular mechanisms underlying the differential functions of this protein in urinary bladder carcinogenesis is highly significant in the field. Here, RhoGDIβ expression patterns, biological functions, and mechanisms leading to transformation and progression of human urothelial cells (UROtsa cells) were evaluated following varying lengths of exposure to the bladder carcinogen *N-butyl-N-*(4-hydmoxybutyl) nitrosamine (BBN).

**Results:**

It was seen that compared to expression in vehicle-treated control cells, RhoGDIβ protein expression was downregulated after 2-month of BBN exposure, but upregulated after 6-month of exposure. Assessments of cell function showed that RhoGDIβ inhibited UROtsa cell growth in cells with BBN for 2-month exposure, whereas it promoted the invasion of cells treated with BBN for 6 months. Mechanistic studies revealed that 2-month of BBN exposure markedly attenuated DNMT3a abundance, and this led to reduced miR-219a promoter methylation, increased miR-219a binding to the *RhoGDIβ* mRNA 3’UTR, and reduced RhoGDIβ protein translation. While after 6-mo of BBN treatment, the cells showed increased PP2A/JNK/C-Jun axis phosphorylation and this in turn mediated overall *RhoGDIβ* mRNA transcription and protein expression as well as invasion.

**Conclusions:**

These findings indicate that RhoGDIβ is likely to inhibit the transformation of human urothelial cells during the early phase of BBN exposure, whereas it promotes invasion of the transformed/progressed urothelial cells in the late stage of BBN exposure. The studies also suggest that RhoGDIβ may be a useful biomarker for evaluating the progression of human bladder cancers.

**Supplementary Information:**

The online version contains supplementary material available at 10.1186/s12915-023-01683-2.

## Background

Bladder cancer (BC) is the tenth most common form of cancer worldwide, with an estimated 573,278 newly diagnosed cases and 212,536 deaths in 2020 [1]. In the USA, a total of 81,400 new cases and 17,980 deaths were estimated in 2020 [2]. Since muscle-invasive bladder cancer (MIBC) can develop to life-threatening metastases and aggressive tumors contributing to nearly 100% of BC-related deaths [3], it is imperative to elucidate the mechanisms underlying BC invasion and metastasis to relieve the mortality of highly invasive human BCs.

Rho family proteins function as molecular switches in various cellular processes, including motility, cytoskeletal organization, cell growth, and differentiation [4]. Rho GDP-dissociation inhibitor (RhoGDI)-β is a guanine nucleotide dissociation inhibitor (GDI) specific for the Rho family of small GTPases. As one of prominent member of the Rho family regulators, RhoGDIβ has been implicated in cancer progression; however, reports have presented contradictory evidence as to the nature of any correlation between cancer progression and RhoGDIβ expression. RhoGDIβ was found to be upregulated in ovarian [5], breast [6], gastric [7], and pancreatic cancer cells that show high perineural invasion [8]. In other studies, RhoGDIβ was reported to suppress BC cell invasion [9], and its expression was inversely correlated with invasion of human BC cells [10]. In human BCs, the functional role of RhoGDIβ is even controversial. Earlier studies found RhoGDIβ as a novel metastasis suppressor gene, and it is mechanistically involved in suppressing tumor invasion and metastasis in model systems of human BC [9, 11]. However, recent studies have shown that RhoGDIβ is elevated in most human BC tissues and all BBN-induced high invasive BCs, facilitating human BC cell invasion and metastasis both in vitro and in vivo [12, 13]. A more precise role for RhoGDIβ in cancer development and subsequent invasive activities remains to be determined.

Accordingly, the study reported here sought to uncover the putative role of RhoGDIβ in BC carcinogenesis. Expression profiles of RhoGDIβ protein in SV40-immortalized human urothelial cells (UROtsa cells) were examined following varying lengths of exposure to the BC-inducing carcinogen *N*-butyl-*N*-(4-hydroxy butyl) nitrosamine (BBN). By characterizing the role of the *RhoGDIβ* gene in regulating cell transformation and tumor invasion in vitro, this study has shown that RhoGDIβ functions as an inhibitor of cell transformation/growth and an activator of malignant progression/invasion in BCs.

## Results

### Biphasic roles of RhoGDIβ in bladder epithelium cells treated with varying lengths of BBN exposures

**Fig. 1:**
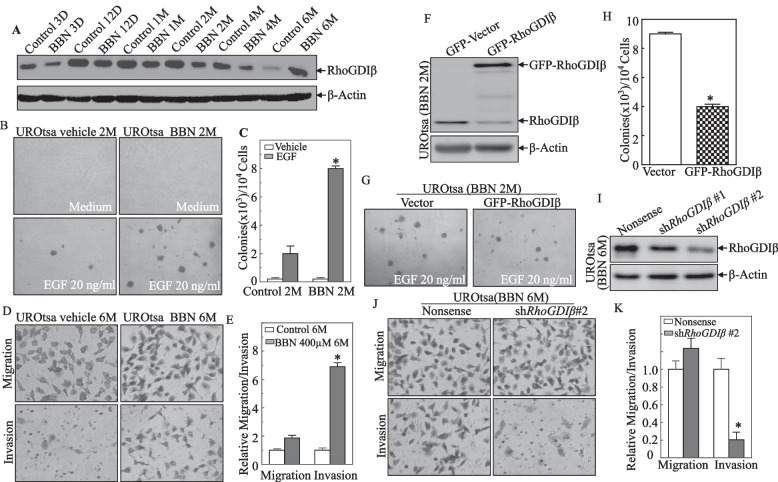
Biphasic roles of RhoGDIβ in UROtsa cells after different BBN exposure lengths. **A** Treatment of UROtsa cells with 400 μM BBN for times indicated. The cell lysates prepared at each time point were evaluated RhoGDIβ expression using Western blot. The bands of RhoGDIβ and β-actin were not derived from the same membrane due to big differential binding intensities of two antibodies. **B**, **C** UROtsa^C2mo^ and UROtsa^BBN2mo^ cells were tested in a soft agar assay in the presence or absence of EGF (20 ng/ml). Representative images of colonies of indicated cells are shown; results are presented as colonies/10^4^ cells seeded. Bars represent mean ± SD of three independent experiments. The asterisk indicates significant difference vs. control group (*p* < 0.05). **D**, **E** UROtsa^C6mo^ and UROtsa^BBN6mo^ cell invasion (Invasion Chamber). The asterisk indicates significant difference between cells. **F** Ectopic RhoGDIβ constructs were stably transfected into UROtsa^BBN2mo^ cells, and the over-expressed efficiency of RhoGDIβ protein was assessed by using Western blot. **G**, **H** Cell anchorage-independent growth of UROtsa^BBN2mo^/Vector and UROtsa^BBN2mo^/GFP-RhoGDIβ cells was determined in soft agar in the presence of EGF (20 ng/ml). Representative images of colonies are shown. The asterisk indicates significant difference vs. vector transfectants (*p* < 0.05). **I** RhoGDIβ knockdown constructs were stably transfected into UROtsa^BBN6mo^ cells, and knockdown efficiency was then determined using Western blot. **J**, **K** UROtsa^BBN6mo^/Nonsense and UROtsa^BBN6mo^/sh*RhoGDIβ*#2 transfectants were subjected to transwell invasion assay. The asterisk indicates significant difference in comparison to UROtsa^BBN6mo^/Nonsense cells

Recent data showed that RhoGDIβ was elevated in most clinical human BC samples in comparison to the paired adjacent normal bladder tissues [12]. However, there still is no direct evidence suggesting a role of RhoGDIβ in bladder carcinogenesis. As shown in Fig. 1A, as compared to the time-paired control cells, RhoGDIβ protein levels were only slightly changed in cells treated for 3 days (D), 12 days, and 1 month (M). However, expression was significantly downregulated with 2 and 4 months of exposure. Interestingly, treatment for 6 months led to an over-expression of the RhoGDIβ protein.

As macroscopic lesions in mouse bladders were observed after 3 months of exposure to BBN (0.05%) [14, 15], the study here exposed the cells to BBN for 2 and 6 months to study effects on bladder cell growth and invasion, respectively. First, to evaluate cell anchorage-independent growth ability, UROtsa (Control 2 months; UROtsa^C2mo^) and UROtsa (BBN 2 months; UROtsa^BBN2mo^) cells were grown in soft agar assay in the presence of EGF. It was seen that a 2-month BBN treatment remarkably increased ability of UROtsa cell anchorage-independent growth in the soft agar as compared with that in treatment with EGF alone (Fig. 1B and C), whereas 2-month BBN treatment did not show any observable effect on invasion ability of UROtsa cells (Figs. S1A and B). In contrast to the observations in 2-month BBN-treated UROtsa cells, 6 months of exposure to BBN led to a significant increase in the invasive ability of the UROtsa cells accompanied by inhibition of anchorage-independent growth (Fig. 1D, E and Fig. S2A, B). Given the dynamic alterations of RhoGDIβ expression in 2 and 6 months of BBN treatment, we anticipate that the decreased RhoGDIβ expression at 2 months of BBN treatment might be associated with the increased anchorage-independent growth of the UROtsa cells, while the increases in RhoGDIβ may promote the invasion in 6-month BBN-treated UROtsa cells.

To test the above hypothesis, we transfected a RhoGDIβ-expressing construct fused with a GFP tag into UROtsa^BBN2mo^ cells (Fig. 1F). Ectopic expression of RhoGDIβ resulted in potent inhibition of anchorage-independent growth of the UROtsa^BBN2mo^ cells (Fig. 1G, H), but did not affect cell migration and invasion (Additional file 1: Fig. S1). In another set of studies, UROtsa^BBN6mo^ cells were transfected with shRNA that specifically targeted human RhoGDIβ; stable UROtsa^BBN6mo^/Nonsense and UROtsa^BBN6mo^/sh*RhoGDIβ* transfectants were established (Fig. 1I). Knockdown of *RhoGDIβ* led to dramatically attenuation of UROtsa^BBN6mo^ cell invasion (Fig. 1J, K), but only had a marginal effect on anchorage-independent growth (Additional file 1: Fig. S2). These results demonstrate the biphasic roles of RhoGDIβ in inhibiting anchorage-independent growth of UROtsa cells followed by BBN treatment for 2 months and in promoting cell invasion by 6 months of BBN exposure, further revealing the differential functions of RhoGDIβ in BBN-induced cell transformation and tumor progression.

### Upregulation of miR-219a contributed to RhoGDIβ protein translation and cell growth inhibition in 2-month BBN-treated cells

**Fig. 2:**
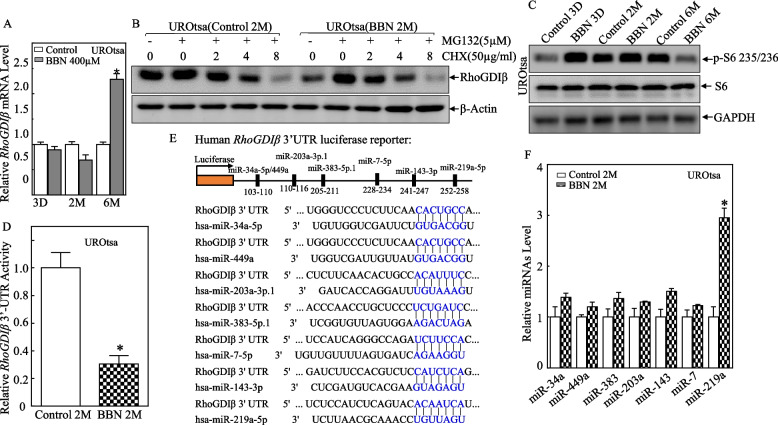
Effects of 2-month BBN treatment on *RhoGDIβ* mRNA 3’-UTR activity and miR-219a expression in UROtsa cells. **A** UROtsa cells were treated with 400 µM BBN for 3 days, 2 months, and 6 months. The cells then had their total RNA extracted and then processed for measures of *RhoGDIβ* mRNA levels using real-time PCR. **B** UROtsa^C2mo^ and UROtsa^BBN2mo^ cells were pre-treated with 5 µM MG132 for 6 h, the cells were then subjected to determining RhoGDIβ protein degradation rates in the presence of CHX (50µg/mL) for different lengths of time. The bands of RhoGDIβ and β-actin were not derived from the same membrane due to big differential binding intensities of two antibodies. **C** Indicated cell extracts were evaluated for levels of S6 phosphorylation at Ser235/236 and total S6 using Western blot. GAPDH was used as a protein loading control. **D** *RhoGDIβ* 3’-UTR-driven luciferase and pRL-TK reporters were transiently co-transfected into UROtsa^C2mo^ and UROtsa^BBN2mo^ cells. The luciferase activity of each transfectant was then evaluated. Bars represent mean ± SD from three independent experiments. The asterisk indicates significant change compared with control transfectant (*p* < 0.05). **E** Potential miRNA binding sites in *RhoGDIβ* mRNA 3’-UTR were predicted using the TargetScan and miRcode databases. **F** Quantitative real-time PCR was used to measure miRNA expression. Bars represent mean ± SD from three independent experiments. The asterisk indicates significant increase in comparison to vehicle control cells (*p* < 0.05)

To investigate the potential underlying mechanisms for the RhoGDIβ regulation, we evaluated the regulatory effects of BBN on the *RhoGDIβ* mRNA. As shown in Fig. 2A, *RhoGDIβ* mRNA expression in the UROtsa cells was slightly reduced by 2 months, but increased by 6 months, following BBN exposure. This result was inconsistent with that observation for the RhoGDIβ protein and suggested that the BBN inhibition of RhoGDIβ protein expression after 2-month exposure was occurring at the level of protein degradation or translation. To determine if this regulation was at the degradation level, the 2-month BBN-treated UROtsa cells and control cells were treated for various time period with the protein biosynthesis inhibitor cycloheximide (CHX; for indicated times) after treatment with MG132 proteasome inhibitor (6 h). As both sets of cells had similar RhoGDIβ protein degradation rates (Fig. 2B), this thus excluded degradation as the means of regulating RhoGDIβ. To determine if RhoGDIβ might be regulated at the translation level, the phosphorylated and total S6 ribosomal protein were evaluated. The results showed the phosphorylation of S6 ribosomal protein was not consistent with RhoGDIβ alterations in the UROtsa^C2mo^ and UROtsa^BBN2mo^ cells (Fig. 2C). As such, possible involvement of S6 ribosomal protein in the noted BBN effects on RhoGDIβ protein translation could be reasonably excluded as occurring.

As miRNA can repress expression by binding to complementary sequences in the 3’ untranslated region (3’-UTR) of target mRNA and so inhibit protein translation [16, 17], a *RhoGDIβ* 3’-UTR luciferase reporter was transiently co-transfected with pRL-TK into UROtsa^C2mo^ and UROtsa^BBN2mo^ cells to evaluate the *RhoGDIβ* mRNA 3’-UTR activity. The result indicated that the *RhoGDIβ* mRNA 3’-UTR activity was markedly reduced in the UROtsa^BBN2mo^ cells in comparison to that in the vehicle control cells (Fig. 2D). Based on these results, TargetScan and miRcode databases were employed to screen for potential miRNA binding sites in the 3’-UTR regions of *RhoGDIβ* mRNA. As shown in Fig. 2E, the bioinformatics analysis indicated that 3’-UTR regions of RhoGDIβ mRNA have multiple miRNA potential binding sites, including miR-34a, miR-449a, miR-383, miR-203a, miR-143, miR-7, and miR-219a. To identify which miRNA might be responsible for the regulation of RhoGDIβ translation, the expression of these various miRNAs in UROtsa^C2mo^ and UROtsa^BBN2mo^ cells was evaluated. The results showed that miR-219a expression was specifically upregulated in the UROtsa^BBN2mo^ cells (Fig. 2F), indicating that it may be involved in downregulation of RhoGDIβ translation in 2-month BBN-treated UROtsa cells.

**Fig. 3:**
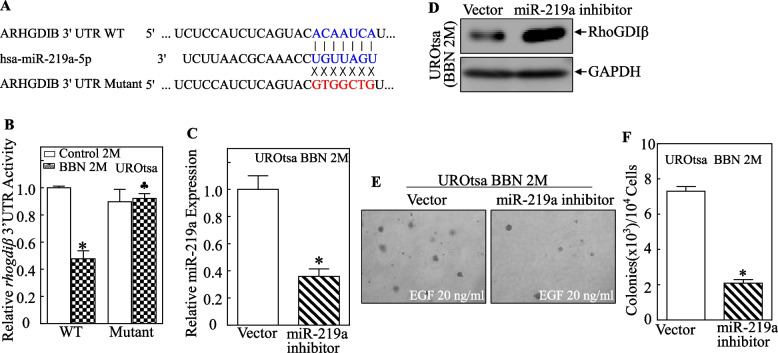
MiR-219a levels, RhoGDIβ protein expression, and anchorage-independent growth among UROtsa cells treated with BBN for 2 months. **A** Schematic of miR-219a binding sites and its mutant in *RhoGDIβ* 3’-UTR luciferase reporter. **B** WT or miR-219a binding site mutant *RhoGDIβ* 3’-UTR luciferase reporter and pRL-TK reporter were transiently co-transfected into UROtsa^C2mo^ and UROtsa^BBN2mo^ cells. The luciferase activity of each transfectant was evaluated and results presented as relative *RhoGDIβ* 3’-UTR activity. The asterisk indicates significant difference from vehicle control (*p* < 0.05). The club symbol indicates significant difference from WT reporter transfectant (*p* < 0.05). **C** miR-219a inhibitor lentivirus was used to infect UROtsa^BBN2mo^ cells, and knockdown efficiency was then determined by using real-time PCR. Results are presented as mean ± SD of triplicate experiments. The asterisk indicates significant decrease vs. control (*p* < 0.05). **D** RhoGDIβ protein expression was evaluated using Western blot. GAPDH was used as a protein loading control. **E**, **F** Representative images of colony formation by UROtsa^BBN2M^ (Vector) and UROtsa^BBN2M^ (miR-219a inhibitor) cells in the soft-agar assay; colonies were captured and scored under an inverted microscope. Results are presented as colonies/10^4^ cells. Bars represent mean ± SD from three independent experiments. The asterisk indicates significant difference vs. control vector transfectant (*p* < 0.05)

To determine if miR-219a specifically targeted the 3’-UTR of *RhoGDIβ* mRNA, a wild-type (WT) *RhoGDIβ* mRNA 3’-UTR luciferase reporter was constructed, and then a point mutation in the miR-219a binding site was generated as illustrated in Fig. 3A. Both WT and mutant reporters were then stably transfected into UROtsa^C2mo^ and UROtsa^BBN2mo^ cells to test whether miR-219a binding to *RhoGDIβ* mRNA 3’-UTR was required for the BBN inhibition of its activity. As shown in Fig. 3B, point mutations in the miR-219a binding site completely reversed BBN-inducible inhibition of luciferase activity, indicating that the miR-219a binding site was crucial for this inhibition. Further, to determine the role of miR-219a in the inhibition of RhoGDIβ protein expression after a 2-mo BBN exposure, a miR-219a inhibitor was stably transfected into UROtsa^BBN2mo^ cells, and miR-219a levels were evaluated as shown in Fig. 3C. Ectopic expression of miR-219a inhibitor profoundly increased RhoGDIβ protein expression (Fig. 3D). It was noted that the miR-219a inhibitor attenuated the anchorage-independent growth of 2-month BBN-treated UROtsa cells (Fig. 3E, F). Collectively, these results showed that BBN treatment induced miR-219a expression, resulting in attenuation of RhoGDIβ protein translation and further resulting in anchorage-independent growth of human urothelial cells.

### Downregulated DNMT3a mediated miR-219a promoter hypomethylation, miR-219a transcriptional upregulation and RhoGDIβ translation inhibition following 2-month BBN exposure

**Fig. 4:**
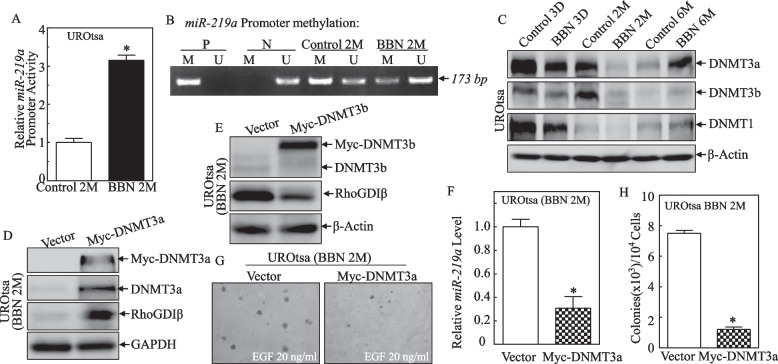
Downregulated DNMT3a-mediated promoter hypomethylation, and miR-219a transcription and RhoGDIβ inhibition in UROtsa cells following a 2-month BBN exposure. **A** UROtsa^C2mo^ and UROtsa^BBN2mo^ cells were transiently transfected with miR-219a promoter-driven luciferase reporter to determine promoter transcriptional activity. Results are presented as miR-219a promoter activity relative to vehicle control (relative miR-219a promoter activity). Bars represent mean ± SD of three independent experiments. The asterisk indicates significant difference vs. vehicle control (*p* < 0.05). **B** Methylation status of miR-219a promoter in UROtsa^C2mo^ and UROtsa^BBN2mo^ cells was determined using methylation-specific PCR [MS-PCR]. A primer set (173bp) was used to evaluate methylated (M) and unmethylated (U) copies of the *miR-219a DMR* gene. Methylated DNA was used as positive control (P); unmethylated control was used as the negative control (N). **C** DNA methyl-transferase DNMT3a, DNMT3b, and DNMT1 expression were evaluated using Western blot in UROtsa cells exposed to BBN (400 μM) for indicated periods. β-Actin was used as a protein loading control. **D**, **E** Ectopic DNMT3a or DNMT3b with Myc-tag constructs were stably transfected into UROtsa^BBN2mo^ cells. The efficiency of over-expressed DNMT3a or DNMT3b on downstream RhoGDIβ protein expression was assessed using Western blot. **F** miR-219a levels in UROtsa^BBN2mo^/Vector and UROtsa^BBN2mo^/Myc-DNMT3a cells were determined by using real-time PCR. Results are presented as the mean ± SD of triplicate experiments. The asterisk indicates significant decrease vs. control (*p* < 0.05). **G**, **H** UROtsa^BBN2mo^/Vector and UROtsa^BBN2mo^/Myc-DNMT3a cells were subjected to soft agar in the presence of EGF (20 ng/ml). The number of colonies was scored, and outcomes presented as colonies/10^4^ seeded cells. The asterisk indicates significant decrease *vs.* vector cells (*p* < 0.05)

Epigenetic modification is implicated in DNA methylation-mediated silencing of miRNAs in human cancer cells [18], and methylation-mediated epigenetic modification in the miR-219 promoter is an essential regulatory mechanism for miR-219 expression in chronic inflammation [19]. Therefore, the current study next investigated whether DNA methylation of the miR-219 promoter was involved in the earlier process of BBN-induced cell transformation. Because CpG islands often co-localize in promoter regions and cytosine methylation of CpG dinucleotides frequently leads to transcriptional silencing [20], a 2-kb segment with the predicted CpG island was cloned into a pGL3-Basic firefly luciferase reporter (miR-219 promoter) and its activity in UROtsa^C2mo^ and UROtsa^BBN2mo^ cells was examined. It was seen that miR-219a promoter activity was upregulated in UROtsa^BBN2mo^ cells compared with in control cells (Fig. 4A), indicating that this region of the promoter contained regulatory elements that control miR-219a transcription. To assess whether upregulation of miR-219a promoter activity after the 2-month BBN exposure was due to promoter methylation, differentially methylated regions (DMR) were examined using methylation-specific PCR (MS–PCR). The results indicated the 2-month BBN treatment caused reductions of methylated DNA (M) and concurrent increases in unmethylated DNA (U) levels in comparison to vehicle control cells under the same experimental conditions (Fig. 4B).

To investigate the potential role of any DNA methyltransferases (DNMTs) on the hypermethylation of miR-219a DMR, potential effects of BBN exposure on DNMTs protein expression were evaluated. The results indicated the 2-month BBN treatment of the UROtsa cells resulted in significantly decreased DNMT3a and DNMT3b protein expression, but no alteration in DNMT1 (Fig. 4C). This was suggestive of a finding that DNMT3a/3b might be involved in the epigenetic methylation of miR-219a promoter. Accordingly, to clarify this, ectopic DNMT3a or DNMT3b with Myc-tag constructs were stably transfected into UROtsa^BBN2mo^ cells. Ectopic overexpression of DNMT3a led to the upregulatory effects of BBN on RhoGDIβ expression in these cells (Fig. 4D), whereas ectopic expression of DNMT3b showed a downregulatory effect on RhoGDIβ expression (Fig. 4E), suggesting that DNMT3a, but not DNMT3b, was associated with RhoGDIβ alteration upon 2-month BBN treatment. This notion was greatly supported by the results that overexpression of DNMT3a resulted in reductions of miR-219a levels in UROtsa^BBN2mo^ cells (Fig. 4F) and also attenuated growth of UROtsa cells after 2-month BBN exposure (Fig. 4G, H). Taken together, our results indicated that downregulation of DNMT3a, but not DNMT3b, was crucial for miR-219 promoter hypomethylation and the consequent mediation of RhoGDIβ translational inhibition as well as anchorage-independent growth (transformation) in 2-month BBN-treated UROtsa cells.

### A 6-month BBN treatment promoted *RhoGDIβ* mRNA transcription by inducting c-Jun phosphorylation

**Fig. 5:**
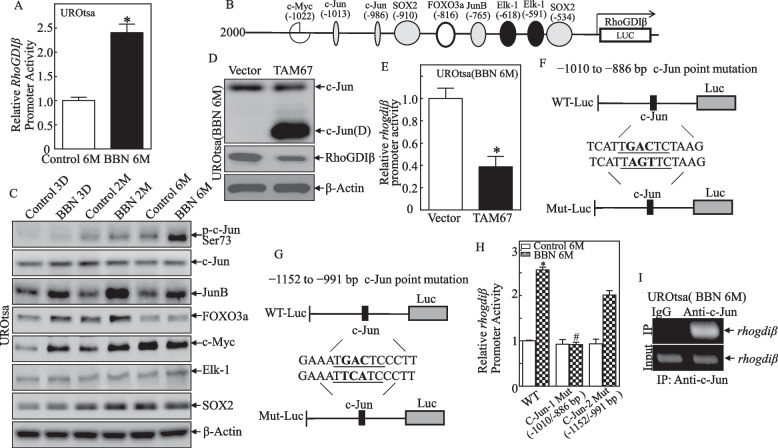
c-Jun phosphorylation mediated RhoGDIβ mRNA transcription in UROtsa cells treated with BBN for 6 months. **A** UROtsa^C6mo^ and UROtsa^BBN6mo^ cells transiently transfected with RhoGDIβ promoter-driven luciferase reporter were used to determine RhoGDIβ promoter transcriptional activity. Results are presented as RhoGDIβ promoter activity relative to vehicle control (relative RhoGDIβ promoter activity). Bars represent mean ± SD from three independent experiments. The asterisk (*) indicates a significant increase from vehicle control (*p* < 0.05). **B** Schematic of putative transcription factor consensus binding sites in the RhoGDIβ proximal promoter region predicted using bioinformatics analysis. **C** Expressions of potential transcription factors were determined by Western blot in UROtsa cells following BBN (400 μM) exposure for the indicated periods. β-Actin was used as a protein loading control. **D**, **E** TAM67 was stably transfected into UROtsa^BBN6mo^ cells, and the stable transfectants were then evaluated for c-JUN (**D**) (TAM67) and RhoGDIβ expression by Western blot (**D**) and RhoGDIβ promoter activity (**E**). **F**, **G** Schematic of two different c-Jun point mutations in RhoGDIβ promoter-driven luciferase reporter. **H** Wild-type RhoGDIβ promoter-driven luciferase reporter or its mutants at the c-Jun binding site were co-transfected with pRL-TK into UROtsa^C6mo^ and UROtsa^BBN6mo^ cells. Luciferase activity of each transfectant was evaluated, and the results were presented as relative RhoGDIβ promoter activity. The asterisk indicates significant increase from control group (*p* < 0.05). The number sign indicates significant decrease between WT and c-Jun-1 Mut in UROtsa^BBN6mo^ cells (*p* < 0.05). **I** ChIP assay using anti-c-Jun antibody to detect the interaction between c-Jun and the RhoGDIβ promoter

To elucidate mechanisms underlying RhoGDIβ upregulation in the UROtsa cells exposed to BBN for 6 months, a wild-type RhoGDIβ promoter-driven luciferase reporter was transfected into UROtsa^C6mo^ and UROtsa^BBN6mo^ cells. The transfectants were then used to determinate the effects of BBN on RhoGDIβ promoter activity. As shown in Fig. 5A, promoter activity was significantly increased in the UROtsa^BBN6mo^ cells in comparison to in the vehicle-treated control cells, indicating that 6-month BBN exposure induced RhoGDIβ transcription in UROtsa cells. Bioinformatics analysis of the RhoGDIβ promoter revealed several potential transcription factors binding sites, including AP-1, FOXO3a, c-Myc, Elk-1, and SOX2 in the promoter region (Fig. 5B). To identify specific transcription factor(s) participating in the modulation of RhoGDIβ transcription, abundance/activation of the related transcription factor proteins was determined. The results showed that 6-month BBN treatment specifically induced marked c-Jun phosphorylation at Ser73 and Jun B protein expression (Fig. 5C), whereas it did not cause any observable increases in FOXO3a, c-Myc, Elk-1, or SOX2. Given previous studies show that Jun B is a negative regulatory transcription factor and Jun B was also induced by BBN exposure at all time points (3 days, 2 months, and 6 months), we anticipated that c-Jun phosphorylation at Ser73 might be involved in transcriptional activation of the RhoGDIβ promoter in 6-month BBN-exposed UROtsa cells.

Based on this observation, a c-Jun dominant-negative mutant expression plasmid TAM67 was transfected into UROtsa^BBN6mo^ cells to determine the potential contribution of c-Jun activation to the induced changes in RhoGDIβ expression. Ectopic expression of the dominant-negative c-Jun (protein product termed c-Jun(D)) successfully blocked *RhoGDIβ* promoter activity and protein expression (Fig. 5D, E). These results reveal the important role of c-Jun activation in BBN-induced upregulation of RhoGDIβ transcription/expression in the UROtsa cells.

To clarify whether the two binding sites of c-Jun on the *RhoGDIβ* promoter region were both required for transcription of *RhoGDIβ* mRNA, point mutations of c-Jun binding sites were introduced into the *RhoGDIβ* promoter as illustrated in Fig. 5F, G, and these mutants and its parental WT reporter were transfected into the UROtsa^C6mo^ and UROtsa^BBN6mo^ cells. The results indicated that promoter transcriptional activity was completely abolished in the transfectant with a -1010/-886 luciferase mutant reporter in comparison to in the transfectant with WT-reporter or the transfectant with the -1152/-991 mutant reporter (Fig. 5H). Such an outcome indicated that the transcription factor binding site at -986 (c-Jun) was critical for *RhoGDIβ* transcription in UROtsa^BBN6mo^ cells. To provide further evidence of c-Jun specifically binding to the *RhoGDIβ* promoter region, a chromatin immunoprecipitation (ChIP) assay was performed using anti-c-Jun antibody. The result shown in Fig. 5I illustrated that c-Jun did directly bind to the putative c-Jun binding sites in the *RhoGDIβ* promoter. Taken together, these data demonstrate that 6-mo BBN treatment of the UROtsa cells induces *RhoGDIβ* mRNA transcription in a c-Jun-dependent fashion.

### PP2A/JNK axis mediated c-Jun protein phosphorylation and activation in 6-month BBN-treated UROtsa cells

**Fig. 6:**
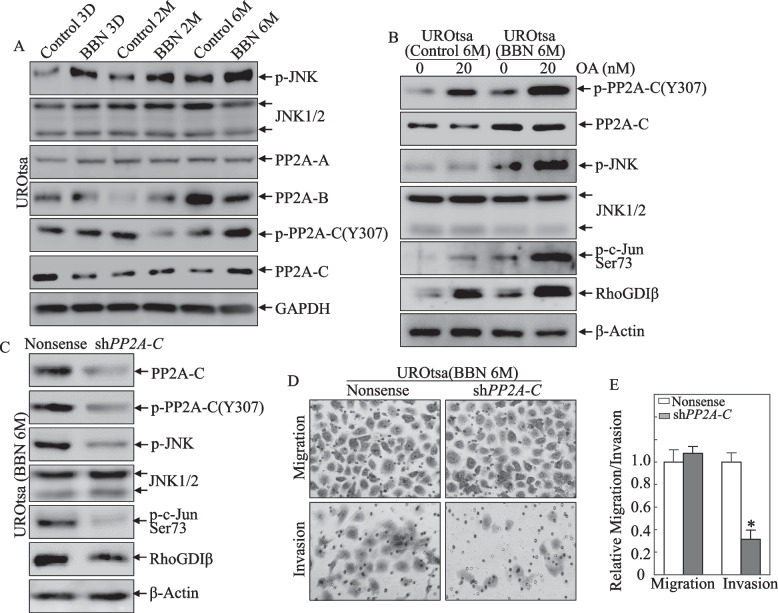
PP2A/JNK axis mediated c-Jun protein phosphorylation and its targeted gene RhoGDIβ transcription, as well as cell invasion. **A** Whole-cell lysates from indicated cells were analyzed for both total protein level and activation levels of JNK and PP2A using Western blot. GAPDH was used as a protein loading control. **B** Indicated cells were treated with okadaic acid (OA) for 6 h, and then whole-cell lysates were evaluated for protein expression by Western blot as indicated. β-Actin was used as a protein loading control. **C** Whole-cell lysates from indicated cells were analyzed for protein expression and activation by Western blot as indicated. β-Actin was used as a protein loading control.** D**, **E** UROtsa^BBN6mo^/Nonsense and UROtsa^BBN6mo^/sh*PP2A-C* transfectants were subjected to transwell invasion assay. The asterisk indicates significant difference in comparison to UROtsa^BBN6mo^/Nonsense cells

C-Jun phosphorylation is regulated by upstream MAP kinase JNK1/2 [21], and protein phosphatase 2 (PP2A) is also reported to regulate c-Jun phosphorylation [22]. To determine if JNK1/2 and/or PP2A were involved in BBN-induced c-Jun activation, total protein levels, and activation of JNK and various units of PP2A were evaluated in both UROtsa^C6mo^ cells *vs.* UROtsa^BBN6mo^ cells. The data showed that activated form JNK (phosphorylation of JNK) and inactivated form of PP2A (PP2A-C subunit at Tyr 307) was remarkably increased in the 6-month BBN-treated cells (Fig. 6A). There were no consistent effects on the other PP2A subunits, PP2A-A and PP2A-B. The results implied that alterations in PP2A phosphorylation in UROtsa cells might be involved in the regulation of JNK/c-Jun phosphorylation after 6-month of BBN exposure.

To test whether inactivation of PP2A in UROtsa^BBN6mo^ cells mediated activation of JNK/c-Jun, UROtsa^C6mo^ and UROtsa^BBN6mo^ cells were treated with specific PP2A and protein phosphatase 1 (PP1) inhibitor Okadaic acid (OA) [23] and then both activation of JNK/c-Jun and RhoGDIβ expression were evaluated. Although treatment of both cell types with OA led to elevations in PP2A-C phosphorylation at Tyr307 and enhanced activation of JNK/c-Jun and increased RhoGDIβ expression (Fig. 6B), these effects observed in UROtsa^BBN6mo^ cells were higher than those observed in UROtsa^C6mo^ cells. This indicated that inactivation of PP2A-C by increased Tyr307 phosphorylation mediated the activation of JNK/c-Jun, and thus RhoGDIβ expression, in UROtsa^BBN6mo^ cells. Furthermore, we used shRNA targeting the PP2A catalytic subunit to specifically knock down PP2A-C expression. The result showed that PP2A knockdown led to inhibition of JNK/c-Jun activation and RhoGDIβ expression in UROtsa^BBN6mo^ cells (Fig. 6C). PP2A-C knockdown also attenuated cell invasion ability, but not the cell migratory effect (Fig. 6D, E). These results strongly suggest that inhibition of PP2A activity via the mediation of its phosphorylation at Tyr-307 by 6-month of BBN treatment resulted in increased c-Jun phosphorylation at Ser73 and transactivation and in turn, leading to RhoGDIβ gene transcription and protein expression in 6-month BBN-treated UROtsa cells.

## Discussion

The UROtsa cell line was derived from the normal urothelium and immortalized with the virus SV40 large T antigen [24], which is used to study the effects of metals and other toxic substances, mostly in the long-term experiment of bladder cancer carcinogenesis. After immortalization, the cells did not acquire characteristics of neoplastic transformation, as noted by the lack of colony formation in soft agar, which could be restored after exposure to tumor promoters such as EGF. EGF has been used as a tumor promoter in many experimental systems, including UROtsa cells [25]. Therefore, we employed the EGF-induced UROtsa cell transformation model to evaluate the effect of RhoGDIβ on anchorage-independent growth following BBN treatment. A growing body of literature shows that passage number affects cell line characteristics over time [26]. Cell lines at high passage numbers experience alterations in morphology, response to stimuli, growth rates, protein expression, and transfection efficiency, compared to lower passage cells. If a comparison is made between the control groups at different time points in the three groups, it will indeed reflect the impact of long-term passage on some characteristics of UROtsa cells. For example, in this study, compared with control 3D and control 2M, the expression of RhoGDIβ (Fig. 1A) and DNA methyltransferase 3 (DNMT3a and DNMT3b; Fig. 4C) was lower, p-JNK and PP2A-B expression was higher (Fig. 6A) in control 6M. Similar abnormal gene expression was also found in long-term cultured UROtsa control cells [27]. To avoid the impact of long-term passage on the effect of BBN exposure, we set up control groups at different time points of 3 days, 2 months, and 6 months, respectively. Only the effects of BBN were explored by comparison between the respective control and BBN-treated groups at various time points.

Variable RhoGDIβ expression has been shown in a variety of human tumor types. Interestingly, the function of RhoGDIβ as a pro- or anti-tumorigenic and/or metastatic protein varies greatly among cancerous tissue types and even in some cases among histological subtypes. For instance, RhoGDIβ expression is downregulated in lung cancer [28], and Hodgkin lymphoma [29], but upregulated in ovarian [5] and stomach cancers [30]. In other tissues, such as the breast, RhoGDIβ expression has been reported to be increased in cancer [31] and that this helps to promote invasive activities of breast cancer cells [32]. Another study found a biphasic pattern of RhoGDIβ expression (increase and then decrease) in breast cancer [33]. A recent report found RhoGDIβ expression upregulated in human ovarian tumors, an outcome that correlated with histological subtype and grade [34]. In the context of human bladder cancers (BC), RhoGDIβ has been mostly thought to act as a suppressor of invasion and metastasis [35]. However, in a recent study from our laboratories, it was seen that there was over-expression of RhoGDIβ in most clinical human BC tissues ( as compared to in paired adjacent normal tissues) and it is as a key XIAP downstream effector mediating bladder cancer (BC) invasion in vitro and in vivo [36]. The novel notion that RhoGDIβ over-expression might be critical to the development and/or malignancy of BC was supported by data from studies of mice treated for ≈6 months (23 weeks) with BBN and who then developed high-invasive BC [36]. Based on those previous findings, it was hypothesized here that RhoGDIβ may play dual roles in BC development, i.e. it is expressed at low levels early resulting in its inhibition on urothelial cell growth and then highly expressed after cancer formation to promote cell invasion/metastasis.

**Fig. 7:**
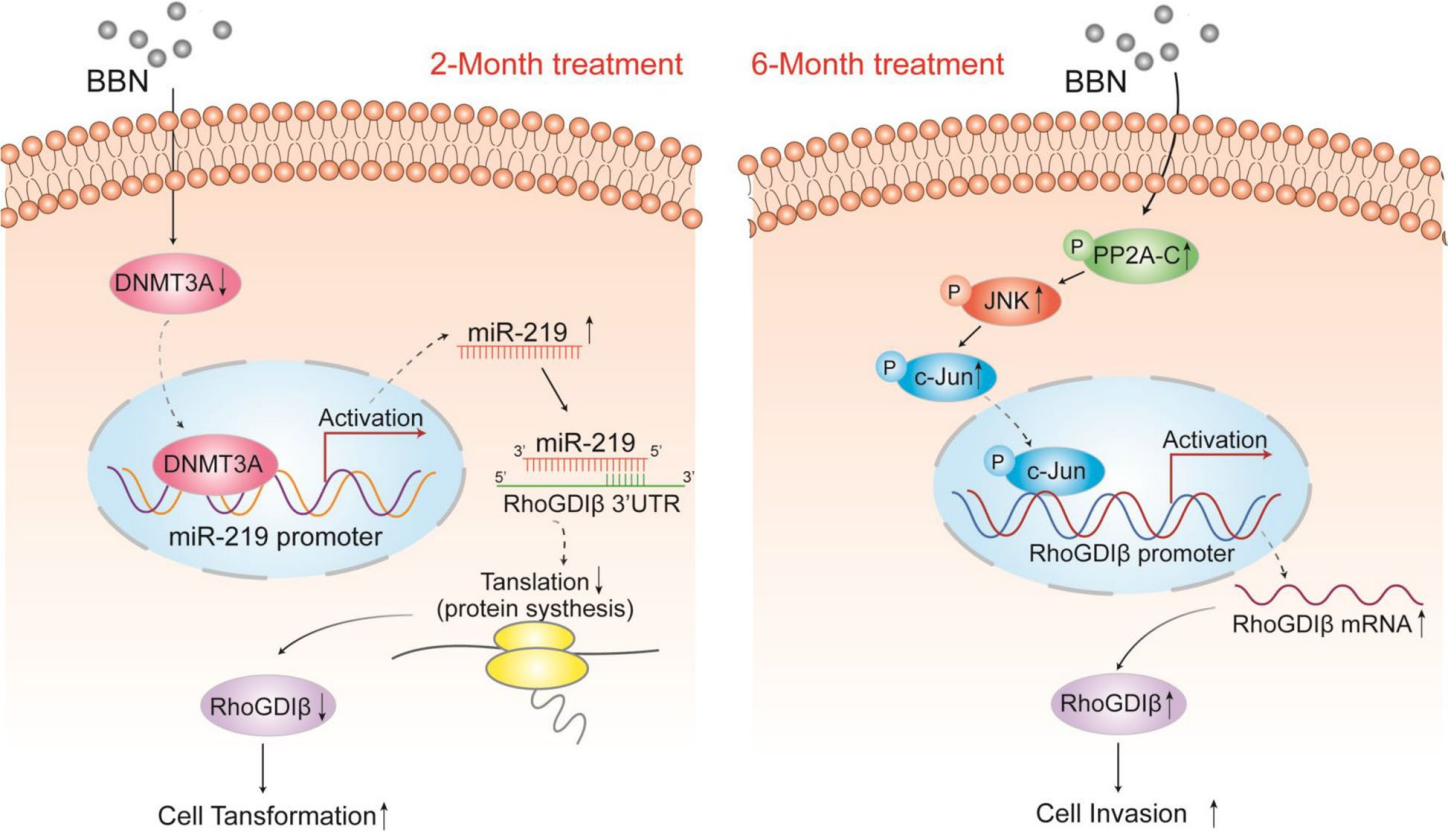
Schematic for potential mechanisms underlying RhoGDIβ mediation of bladder epithelial cell growth and malignant progression as a result of different lengths of BBN exposure

The present study derived evidence suggesting that RhoGDIβ could function as a biphasic regulator of tumor development in BC. First, it was shown that RhoGDIβ protein expression was significantly downregulated after a 2-month exposure of UROtsa cells to BBN but then over-expressed after 6-month treatment. Second, it was documented that ectopic expression of RhoGDIβ inhibited cell growth of the UROtsa cells treated with BBN for 2 months, but it then promoted the invasive ability of the cells after 6-month of treatment. Further, the analyses here revealed that a 2-month BBN exposure markedly attenuated DNMT3a protein levels in the cells, in turn reducing miR-219a promoter methylation and further increasing miR-219a transcription and its binding directly to the *RhoGDIβ* mRNA 3’-UTR, thereby impeding RhoGDIβ protein translation. In contrast, a 6-month BBN treatment promoted PP2A-C/JNK/C-Jun axis phosphorylation that then led to RhoGDIβ mRNA transcription and induction (Fig. 7).

RHOGDIs mainly regulate RHO proteins through either as negative regulators of RHO GTPases, or as chaperone protein or shuttling RHO GTPases between membranes, by which RHOGDIs regulate various cellular processes, including cell adhesion, migration, and proliferation [37]. The RHOGDIs family usually conducts cross-talk with the RAS superfamily, linking intracellular signal transduction pathways with changes in the external environment [38]. In a recent article [39], it was found that oncogenic RAS can mediate the reprogramming of cellular metabolism to support the energy and biomass demands of uncontrolled proliferation, a hallmark of cancer. Therefore, we speculate that RHOGDIs may play an important role in RAS-dependent control of cellular metabolism, which is worth exploring in the future.

Despite a large body of molecular and functional evidence supporting the alteration of miRNA expression in several physiological and pathological processes, little is known about how the miRNA gene expression itself is regulated in these processes. DNA methylation is an important epigenetic modification in the mammalian genome. The growing evidence has linked DNA methylation with some human diseases, including cancer [18]. It has been reported that DNA methylation alterations contribute to the deregulation of 12 cancer-associated miRNAs and breast cancer progression [40]. Also, methylation of a variety of miRNA genes in urine specimens could serve as a useful and noninvasive biomarker for accurate detection of bladder cancer [41]. Therefore, DNA methylation might be involved in mediating the pathologies associated with BC development. In vitro and in vivo studies showed that DNA CpG methylation specifically silences miRNA expression in human cancer cells [18]. Considering that 50% of miRNA promoters have CpG islands, miRNA promoter region methylation might be an important mechanism by which miRNA transcription is regulated. In this study, we found the upregulation of miR-219a promoter activity in 2-month BBN exposure is due to the hypomethylation of CpG islands in the miR-219 promoter. Ectopic overexpression of DNMT3a led to the inhibition of miR-219a levels and the upregulatory effects on its target gene RhoGDIβ protein expression, as well as the attenuation of cell growth in UROtsa^BBN2mo^ cells (Fig. 4). This study primarily reported that the DNMT3a-mediated epigenetic methylation is critical for miR-219 expression and its functional significance in early bladder carcinogenesis following bladder carcinogen BBN exposure.

## Conclusions

In conclusion, this study expands our knowledge about the functional role of miR-219a and elucidates a novel mechanism for regulation of miR-219a expression in the development of bladder cancer. Moreover, this study also showed that RhoGDIβ expression correlated with BC progression and invasiveness. Thus, RhoGDIβ may prove to be an important molecule in understanding the biology associated with the early BC development and potential late phase progression and metastasis of BC cells.

## Materials and methods

### Plasmids, antibodies, and reagents

Short hairpin RNAs (shRNAs) specific targeting human RhoGDIβ and protein phosphatase type 2A C subunit (PP2A-C) were purchased from Open Biosystems (Pittsburgh, PA). Generation of the c-Jun dominant-negative mutant (TAM67) was described in our previous studies [42, 43]. The GFP-RhoGDIβ expression vector [44] and its scramble control were kind gifts from Dr. Martin A. Schwartz (Robert M. Berne Cardiovascular Research Center, University of Virginia, Charlottesville, Virginia, USA). The human *RhoGDIβ* mRNA 3’-UTR luciferase reporter was constructed in a pMIR-Report luciferase reporter. The human miR-219a promoter (−770 to +362) was cloned into the pGL3-basic luciferase reporter. The miR-219a inhibitor was purchased from Invitrogen (Waltham, MA, USA). Plasmids were prepared using the Plasmid Preparation/Extraction Maxi kit (QIAGEN, Valencia, CA, USA).

Antibodies specific against Phospho (p)-S6 Ribosomal Ser235/236(4858S), p-c-Jun Ser73 (3270S), c-JUN (9165S), c-Jun(D) (5000S), Elk-1 (9182S), FOXO3a (2497S), SOX2 (23064S), PP2A-B (2290S), PP2A-A (2041S), and JNK1/2(9258S) were all purchased from Cell Signaling Technology (Beverly, MA, USA). Antibodies specific against RhoGDIβ/Ly-GDI (sc-11359), DNMT3a (sc-373905), JunB (sc-46), c-Myc (sc-40), and GAPDH (sc-25778) were all purchased from Santa Cruz Biotechnology (Santa Cruz, CA, USA). Antibodies specific against p-PP2A (pY307) (1155-1) and PP2A-C (1512-1) were purchased from Epitomics (Burlingame, CA, USA). Antibody against Phospho-JNK1/2(Thr183, Tyr185) was obtained from Invitrogen. Antibody against β-Actin (ab8224) was obtained from Abcam Corporation (Cambridge, MA, USA).

The carcinogen *N-butyl-N-*(4-hydroxybutyl)-nitrosamine (BBN) (#B0938) was purchased from TCI AMERICAN (Cambridge, MA, USA). Proteasome inhibitor MG132 (#S2619) was bought from Selleckchem (Houston, TX, USA); protein synthesis inhibitor cycloheximide (CHX) (#sc-3508) was purchased from Santa Cruz Biotechnology. Okadaic acid was purchased from Santa Cruz Biotechnology. The dual-luciferase assay substrate was obtained from Promega (Madison, WI, USA).

### Cell culture and transfections

The SV40-immortalized human urothelial cell line UROtsa was a kind gift from Dr. Scott Garrett (Department of Pathology School of Medicine and Health Sciences, University of North Dakota, USA) [24] and was used in our previous publication [25]. These cells were maintained at 37°C in a 5% CO_2_ incubator with RPMI medium 1640 medium supplemented with 10% fetal bovine serum (FBS; 26140079), a mixture solution of 2 mM L-glutamine, 100 IU penicillin, and 100 μg/ml streptomycin (CORNING, 30-009-CI, USA). All cell lines were subjected to DNA tests and authenticated every 6–12 months by verifying viability, recovery, growth, morphology, and chemical response as well as testing STR loci and sex using a PowerPlex® 16 HS System provided by Genetica DNA Laboratories (Cincinnati, OH, USA). Using the carcinogen BBN that targets urothelial cells [36, 45], this study evaluated the RhoGDIβ protein abundance in UROtsa cells treated with BBN (400 µM) varying periods of time.

Transfections were carried out with specific plasmid constructs using PolyJet™ DNA in vitro Transfection Reagent (SignaGen Laboratories, Gaithersburg, MD, USA) according to the manufacturer’s instructions. Stable transfection selection of GFP-RhoGDIβ, shRhoGDIβ, TAM67, and miR-219a inhibitor in UROtsa cells was performed with puromycin (0.2-0.3 μg/ml) or G418 (500-1000 μg/ml), depending on the antibiotic resistance plasmid transfected; surviving stable transfectants were pooled as stable mass culture.

### Treatment of UROtsa cells with BBN in vitro

UROtsa is monolayer adherent cells and the cells were treated with either vehicle control (0.1% DMSO) or 400 μM BBN till 95% confluence. The BBN-treated cells were passaged and 1/6 of the cells were retained to be treated with either 400 μM BBN or vehicle (0.1% DMSO) for approximately 3–4 days to reach a ~95% cell density. The culture cells were continued to be treated in this manner for up to 6 months and the cells were then collected for further analyses.

### Western blot

UROtsa cells and their transfectants with or without BBN treatment were extracted with cell lysis buffer (10 mmol/L Tris-HCl [pH 7.4], 1% SDS, and 1 mmol/L Na_3_VO_4_) supplemented with protease inhibitor (#PIA32965, Thermo Scientific, Waltham, MA, USA). After clearing of debris by centrifugation, protein levels in the resulting supernatant were determined using a NanoDrop 2000 spectrophotometer (Thermo Scientific, Waltham, MA, USA). Based on protocols used in an earlier study [46, 47], aliquots of the whole-cell extracts were then subjected to SDS-PAGE and then electrotransferred to polyvinylidene fluoride (PVDF) membranes (Bio-Rad, Hercules, CA, USA). A given protein band specifically bound to its targeting primary antibody was ultimately detected on the membrane using an alkaline phosphatase-linked secondary antibody. The membranes then underwent ECF substrate (#RPN5785, GE Healthcare, Chicago, IL, USA) and then were scanned in a Typhoon FLA 7000 (GE Healthcare, Chicago, IL, USA) biomolecular imager. The acquired images were cropped and leveled using the official supporting image analysis software TotalLab TL100. It was specifically described in the legends to the figures/supplementary files if some protein bands shown in the figures were not run on the same gel/blot membrane or the same batch of protein samples. The uncropped gel images were presented in Additional file 2.

### Quantitative real-time PCR

Total RNA was extracted with TRIzol reagent (Invitrogen) according to the manufacturer’s protocols. cDNAs were then synthesized using a Thermo-Script RT-PCR system (Invitrogen). mRNA amounts were measured by quantitative real-time PCR. The primers used here for human *RhoGDIβ* were 5’-ACC CGG CTC ACC CTG GTT TGT-3’ (Forward) and 5’-ACC CCA GTC CTG TAG GTG TGC TG-3’ (Reverse). Total microRNA was extracted using the miRNeasy Mini Kit (Qiagen). Analysis of miR-34a, -449, -383, -203a, -143, -7, and -219 expression was conducted using the miScript PCR Starter Kit and miScript PCR kit II RT Kit (Qiagen) following manufacturer protocols. U6 was always used as the endogenous normalizer. Initial activation was performed at 95℃ for 15 min, followed by 40 cycles of denaturation at 95℃ for 15 s, annealing at 55℃ for 30 s, and extension at 70℃ for 30 s. Cycle threshold (C_t_) values were determined, and the relative expression of microRNAs calculated using values of 2^-△△Ct^, as described earlier [48, 49].

### Luciferase reporter assay

Cells were transfected with the indicated luciferase reporter in combination with the pRL-TK vector (Promega) as an internal control. Luciferase activity was determined with a microplate luminometer. All experiments were performed in triplicate. All results were expressed as the mean±SD.

### Soft-agar assay

A total of 10^4^ cells/protocol were mixed with epidermal growth factor (EGF; final concentration = 20 ng/ml) or vehicle control (1‰ DMSO) in 2% FBS-Basal Medium Eagle (BME) containing 0.33% agar. The cells were then seeded over a basal layer of 0.5% agar in 2% FBS/BME in each well of the 6-well plate. The plates were then incubated in 5% CO_2_ at 37℃ for 4 weeks. Colonies were then identified using a CKX41 light microscope (Olympus, Tokyo, Japan); only colonies containing ≥ 32 cells were counted. The results are presented as mean ± SD obtained from three independent experiments.

### Cell invasion assay

A cell invasion assay was performed using the BD BioCoat™ Tumor Invasion System (BD Falcon, NY, USA) according to the manufacturer’s instruction. In brief, cells (3×10^4^) were simultaneously seeded (in triplicate) onto chamber inserts coated with Matrigel™ matrix (invasion) or uncoated (migration) in 500 μL of serum-free RPMI 1640. The inserts were then placed into wells containing 700 µL RPMI 1640 supplemented with 10% FBS. The cells were incubated for 24 h, and then cells on both the inside and outside of the chamber were fixed with 3.7% formalin for 5 min, washed twice, treated with 100% methanol for 20 min, washed twice again, and then stained with Giemsa (1:20 in PBS) for 30 min in the dark. The number of cells that had migrated (attached to the other side of the insert) was counted using a CKX41 microscope, a total of three random fields was examined in each case, each at 200× magnification. The number of migrated and invasive cells per image was then determined using Image J software. Data are presented as percentage invasion through the BD Matrigel™ matrix and membrane relative to the migration of cells through the uncoated membrane.

### DNA extraction, bisulfite DNA modification, and methylation-specific PCR

CpG islands (dinucleotide-rich regions) were predicted using MethPrimer 2.0 (http://www.urogene.org/tool.html) for the upstream region of precursor miR-219a (pre-miR-219a). Genomic DNA from UROtsa^C2mo^ and UROtsa^BBN2mo^ cells was extracted using a DNeasy Blood and Tissue Kit (# 69504, Qiagen). Sodium bisulfite modification of DNA and subsequent purification was performed according to the manufacturer instructions for bisulfite conversion of unmethylated cytosines in DNA (EpiTect Bisulfite kit; #59104, Qiagen).

The bisulfite-treated genomic DNA was then subjected to an optimized methylation-specific PCR protocol, i.e. 20 µl reactions containing 10 ng template, 10 µl 2× EpiTect Master Mix (Qiagen) and 0.4 µM of a given set of methylation primers: (MF, GTT GTA GTC GGT TTG GGG TC; and MR, CCA ATC CTT ATA TAA ACG CCA) or 0.4 µM each unmethylation primers (UF, GTA GTT GGT TTG GGG TTG GA; and UR, TAA CCA ATC CTT ATA TAA ACA CCA). Touchdown PCR was then performed as follows: 95℃ for 10min followed by five cycles of 94℃ for 30 s, 70℃ for 30 s, 72℃ for 30 s; five cycles of 94℃ for 30 s, 65℃ for 30 s, 72℃ for 30 s; and 30 cycles of 94℃ for 30 s, 60℃ for 30 s, 72℃ for 30 s; Final extension was performed at 72℃ for 7 min. All products were then separated over 2% high-resolution agarose gels and visualized by ethidium bromide staining. All PCR products were run in duplicate with an EpiTect PCR Control DNA Set (#59695, Qiagen).

### Chromatin immunoprecipitation (ChIP) assay

ChIP was performed using an EZ-CHIP kit (Millipore Technologies, Burlington, MA, USA), as described in our previous publication [48]. In brief, UROtsa^BBN6M^ cells genomic DNA and the proteins were cross-linked with 1% formaldehyde. The cross-linked cells were pelleted, resuspended in cell lysis buffer, and sonicated to generate 200–500 bp chromatin DNA fragments. After centrifugation, the supernatants were diluted 10-fold and then incubated overnight with either anti-c-Jun antibody or the control (i.e. nonspecific rabbit IgG) at 4°C. Any immune complex that had formed was then captured using columns containing protein A/G-agarose saturated with salmon sperm DNA, then eluted with the elution buffer. The complexes were then separated by heating overnight at 65°C, and the liberated DNA was then purified by PCR. To specifically amplify the region containing the putative responsive elements on the human RhoGDIβ promoter, PCR was performed with the primers: 5’-CAT TCA CCC GAG GCG GAC TA-3’ (Forward), 5’-CGG TCC AGC CAA TCA GAG GT-3’ (Reverse). The resulting PCR products were separated over 2% agarose gels, stained with ethidium bromide, and UV-light-based images were then captured/analyzed in an SP Image system (Alpha Innotech Corporation, San Leandron, CA, USA).

### Statistical analysis

All data are reported as mean ± SD unless indicated elsewise. A Student’s *t*-test was used to determine significant differences between treatment groups. In all cases, a *p*-value < 0.05 was considered significant. All data were analyzed using SPSS software (IBM, NY, USA).

### Supplementary Information

**Additional file 1: Fig. S1.** Transwell invasion assay of UROtsa^BBN2mo^(Vector) and UROtsa^BBN2mo^(GFP-RhoGDIβ) cells. **Fig. S2.** [Soft agar assay of UROtsa^C6mo^, UROtsa^BBN6mo^(Nonsense) and UROtsa^BBN6mo^(shRhoGDIβ #2) cells with/without EGF.

**Additional file 2.** Uncropped gel images.

## Data Availability

The datasets supporting the conclusions of this article are included within the article and its additional files.

## Abbreviations

BC: Bladder cancer
CHX: Cycloheximide
EGF: Epidermal growth factor
FBS: Fetal bovine serum
OA: Okadaic acid
RT-PCR: Reverse transcription-polymerase chain reaction
shRNA: Short hairpin RNA
3’-UTR: 3’-Untranslated regions
