# Supplementary material for: Differential functions of RhoGDIβ in malignant transformation and progression of urothelial cell following N-butyl-N-(4-hydmoxybutyl) nitrosamine exposure
Source: BMC Biol. 2023 Aug 28;21:181. doi: 10.1186/s12915-023-01683-2 (PMC10463823; doi:10.1186/s12915-023-01683-2)
Supplement: Supplementary file 1 — Additional file 1: Fig. S1. Transwell invasion assay of UROtsaBBN2mo(Vector) and UROtsaBBN2mo(GFP-RhoGDIβ) cells. Fig. S2. [Soft agar assay of UROtsaC6mo, UROtsaBBN6mo(Nonsense) and UROtsaBBN6mo(shRhoGDIβ #2) cells with/without EGF. [file 12915_2023_1683_MOESM1_ESM.pdf]

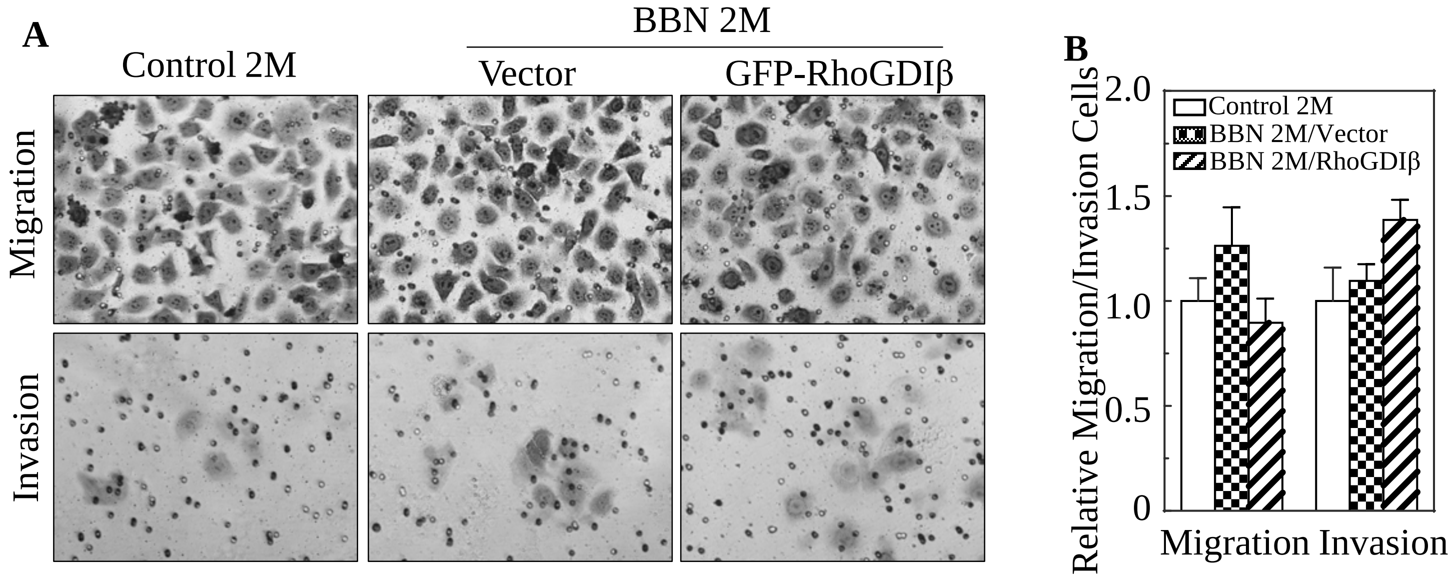

(**A, B**) UROtsa^{BBN2mo}(Vector) and UROtsa^{BBN2mo}(GFP-RhoGDIβ) cells were subjected to transwell invasion assay.

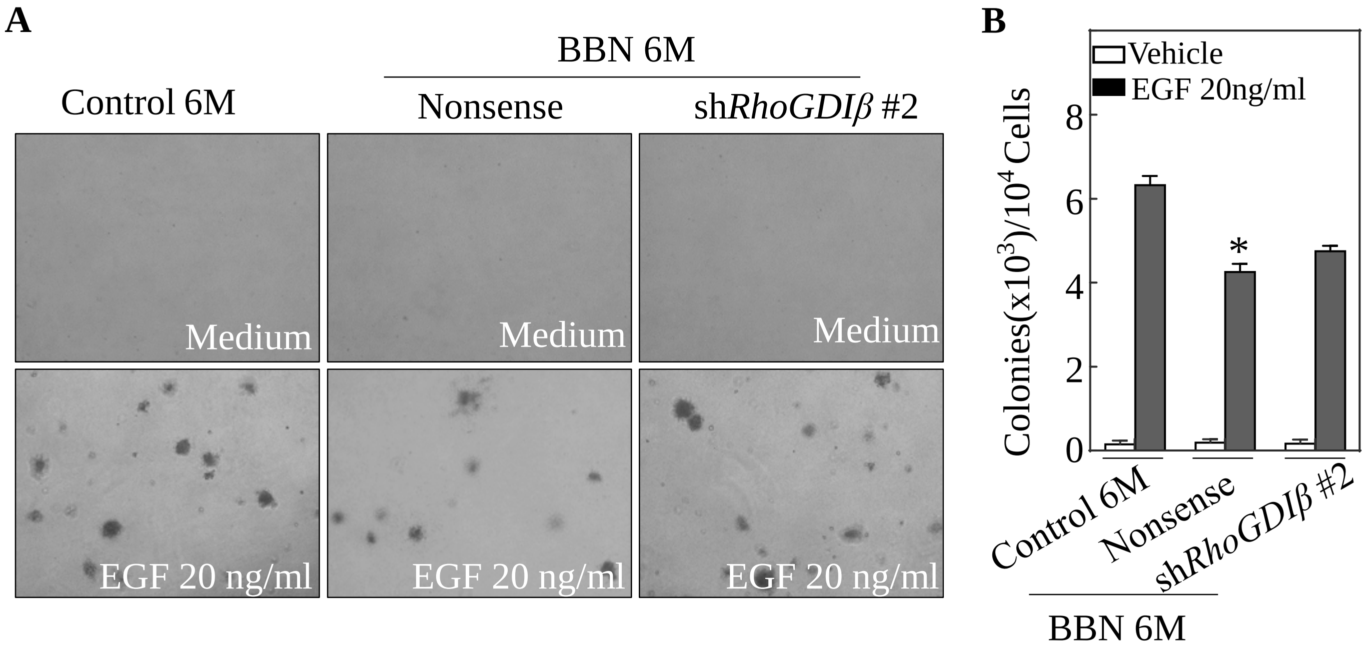

(**A, B**) UROtsa^{C6mo}, UROtsa^{BBN6mo}(Nonsense) and UROtsa^{BBN6mo}(sh*RhoGDIβ* #2) cells were tested in a soft agar assay in the presence or absence of EGF (20 ng/ml). Representative images of colonies of indicated cells are shown; results are in colonies/10^4 cells seeded. Bars represent Mean $\pm$ SD of three independent experiments. * Significant difference *vs.* control group ($p < 0.05$).
