# Supplementary material for: Differential functions of RhoGDIβ in malignant transformation and progression of urothelial cell following N-butyl-N-(4-hydmoxybutyl) nitrosamine exposure
Source: BMC Biol. 2023 Aug 28;21:181. doi: 10.1186/s12915-023-01683-2 (PMC10463823; doi:10.1186/s12915-023-01683-2)
Supplement: Supplementary file 2 — Additional file 2. Uncropped gel images. [file 12915_2023_1683_MOESM2_ESM.pptx]

## Slide 1
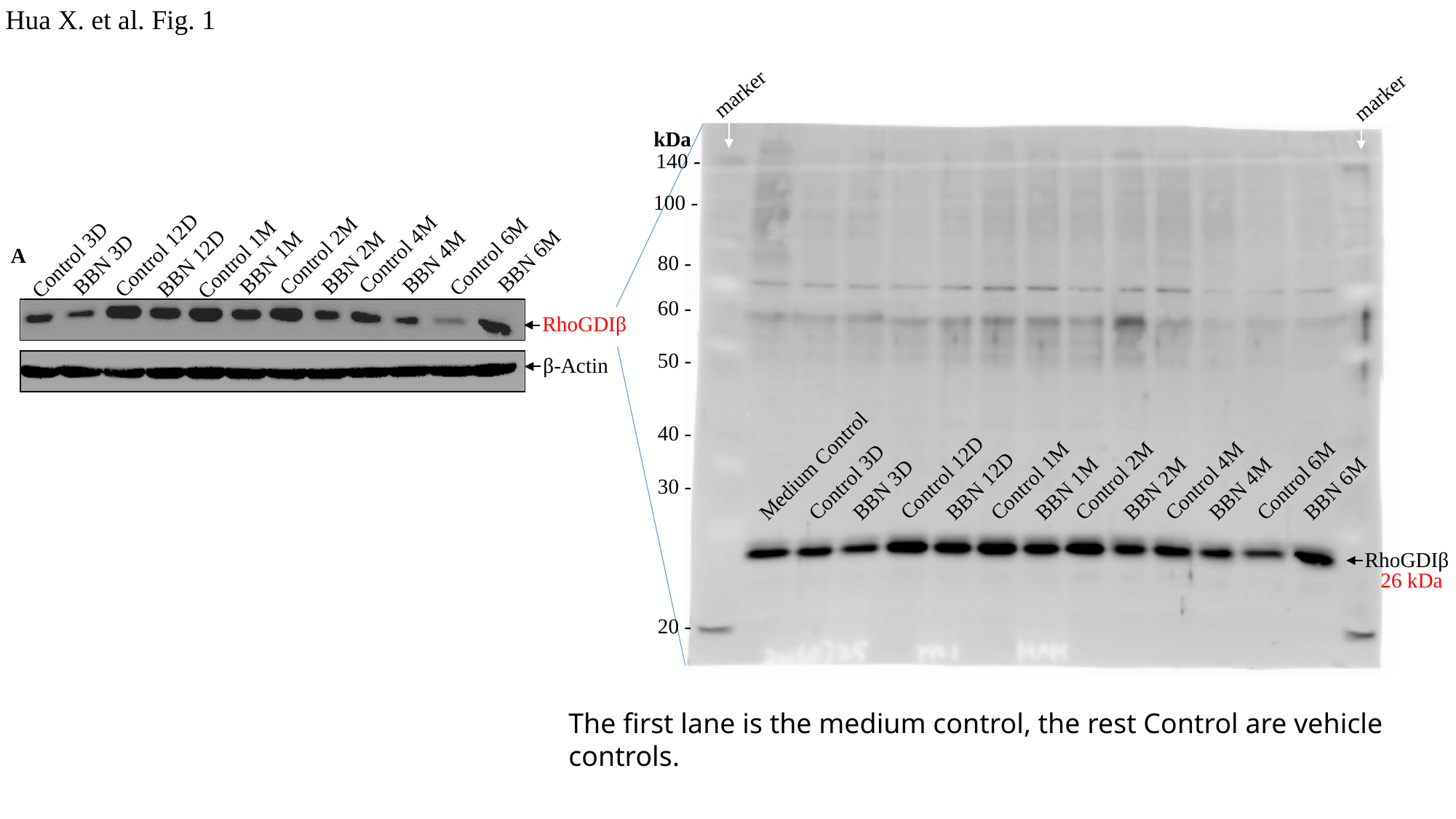

Hua X. et al. Fig. 1
marker
marker
kDa
140 -
100 -
Control 12D
Control 6M
Control 2M
Control 1M
Control 4M
A
BBN 3D
BBN 6M
BBN 2M
Control 3D
BBN 4M
BBN 12D
BBN 1M
80 -
60 -
RhoGDIβ
β-Actin
50 -
40 -
Medium Control
Control 12D
Control 6M
Control 1M
Control 2M
Control 4M
Control 3D
BBN 3D
BBN 12D
BBN 2M
BBN 6M
BBN 4M
BBN 1M
30 -
RhoGDIβ
26 kDa
20 -
The first lane is the medium control, the rest Control are vehicle controls.

## Slide 2
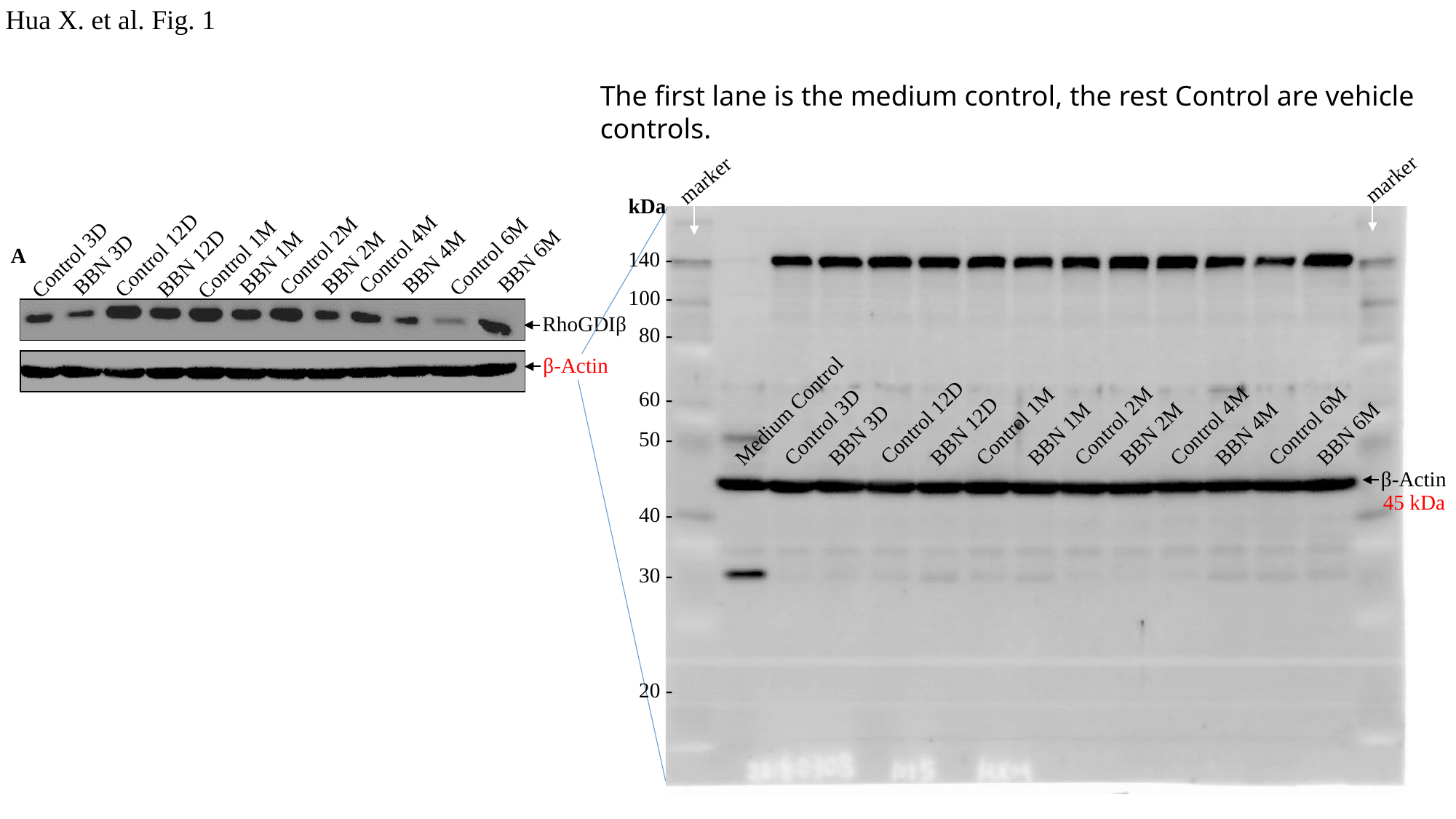

Hua X. et al. Fig. 1
The first lane is the medium control, the rest Control are vehicle controls.
marker
marker
kDa
Control 12D
Control 6M
Control 2M
Control 1M
Control 4M
A
BBN 3D
BBN 6M
BBN 2M
Control 3D
BBN 4M
BBN 12D
BBN 1M
140 -
100 -
RhoGDIβ
80 -
β-Actin
60 -
Medium Control
Control 12D
Control 6M
Control 1M
Control 2M
Control 4M
Control 3D
BBN 3D
BBN 12D
BBN 2M
BBN 6M
BBN 4M
BBN 1M
50 -
β-Actin
45 kDa
40 -
30 -
20 -

## Slide 3
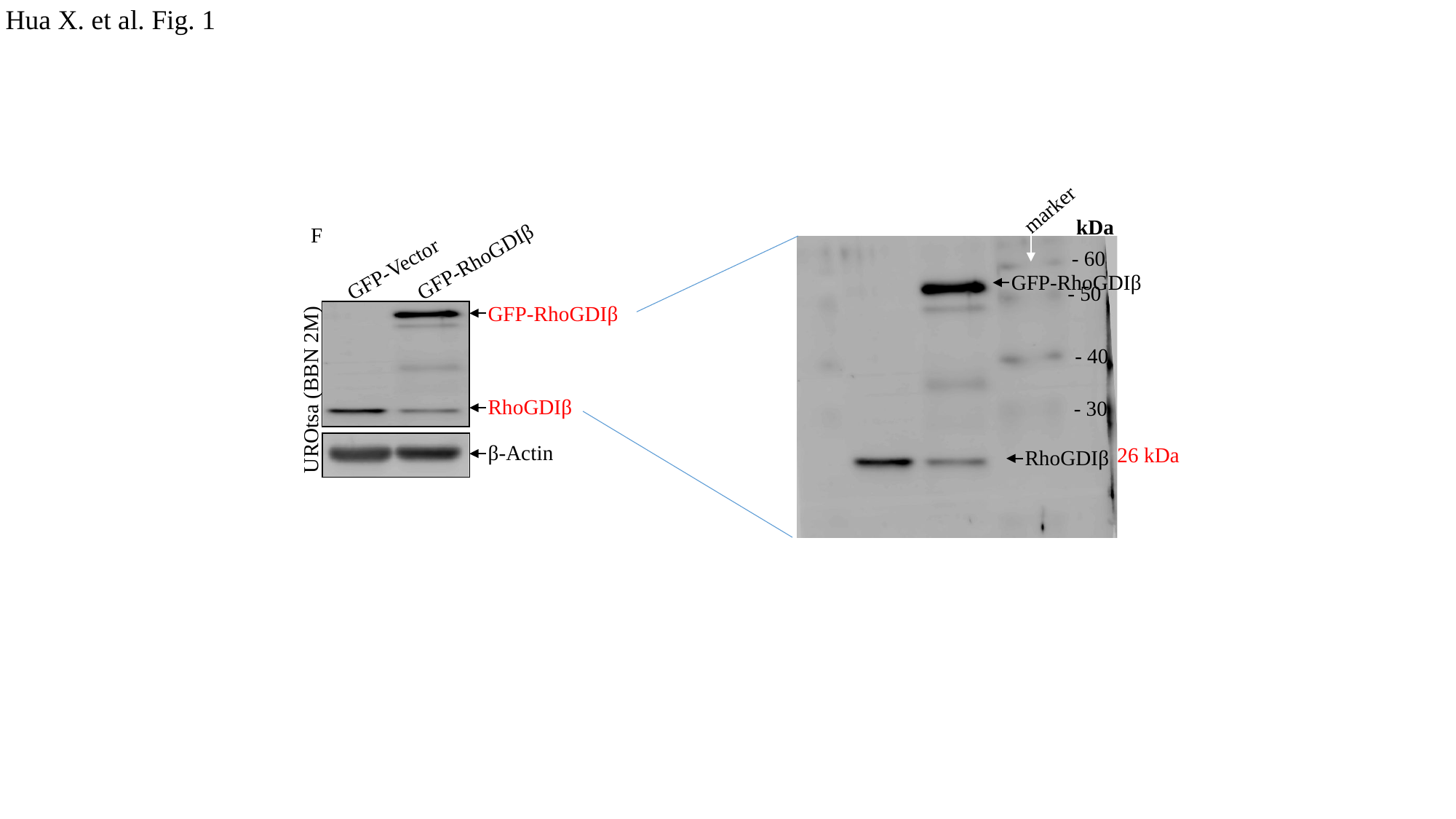

Hua X. et al. Fig. 1
marker
kDa
F
GFP-RhoGDIβ
- 60
GFP-Vector
GFP-RhoGDIβ
- 50
GFP-RhoGDIβ
- 40
UROtsa (BBN 2M)
RhoGDIβ
- 30
β-Actin
RhoGDIβ
26 kDa

## Slide 4
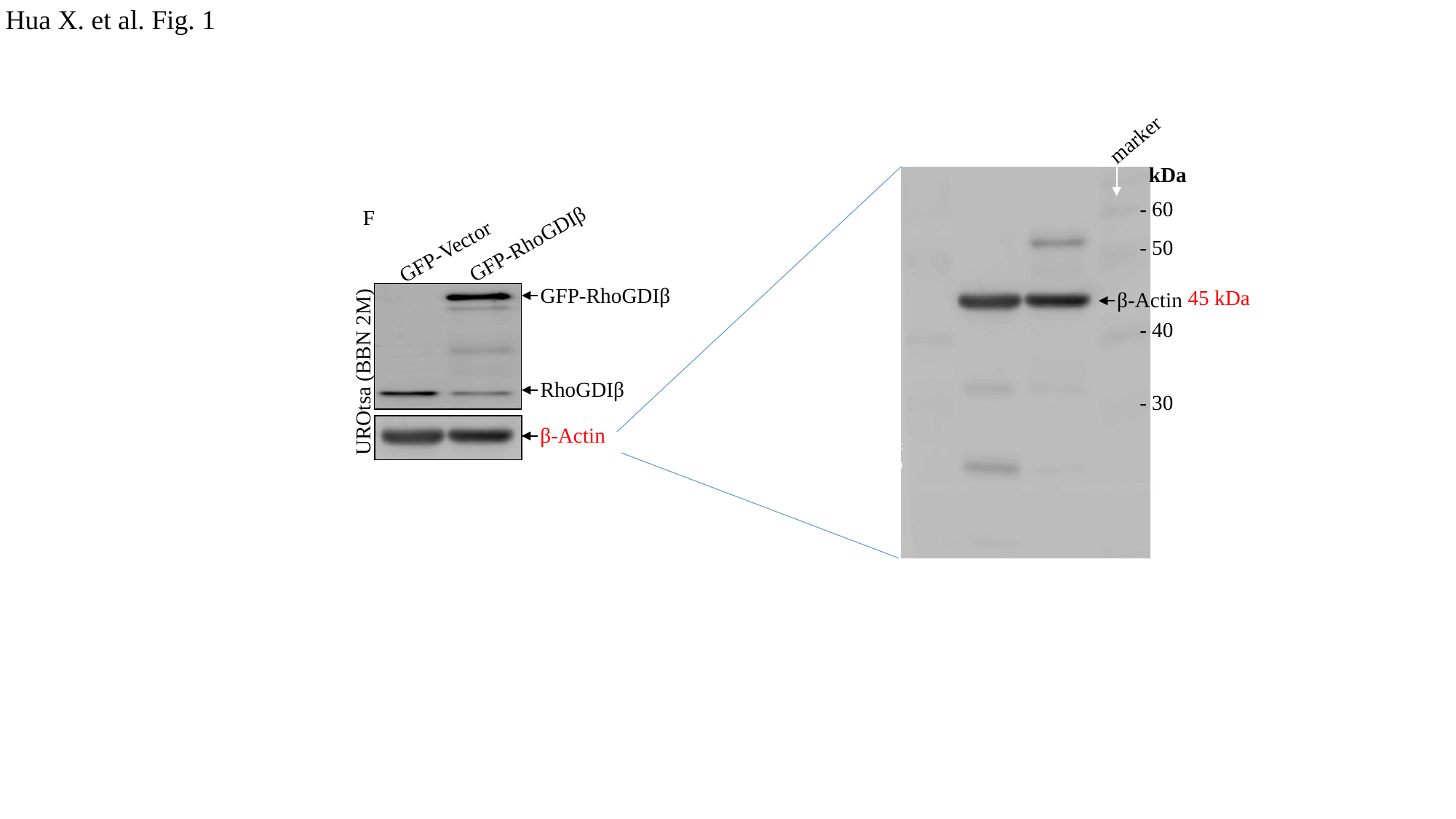

Hua X. et al. Fig. 1
marker
kDa
- 60
F
GFP-RhoGDIβ
GFP-Vector
- 50
GFP-RhoGDIβ
β-Actin
45 kDa
- 40
UROtsa (BBN 2M)
RhoGDIβ
- 30
β-Actin

## Slide 5
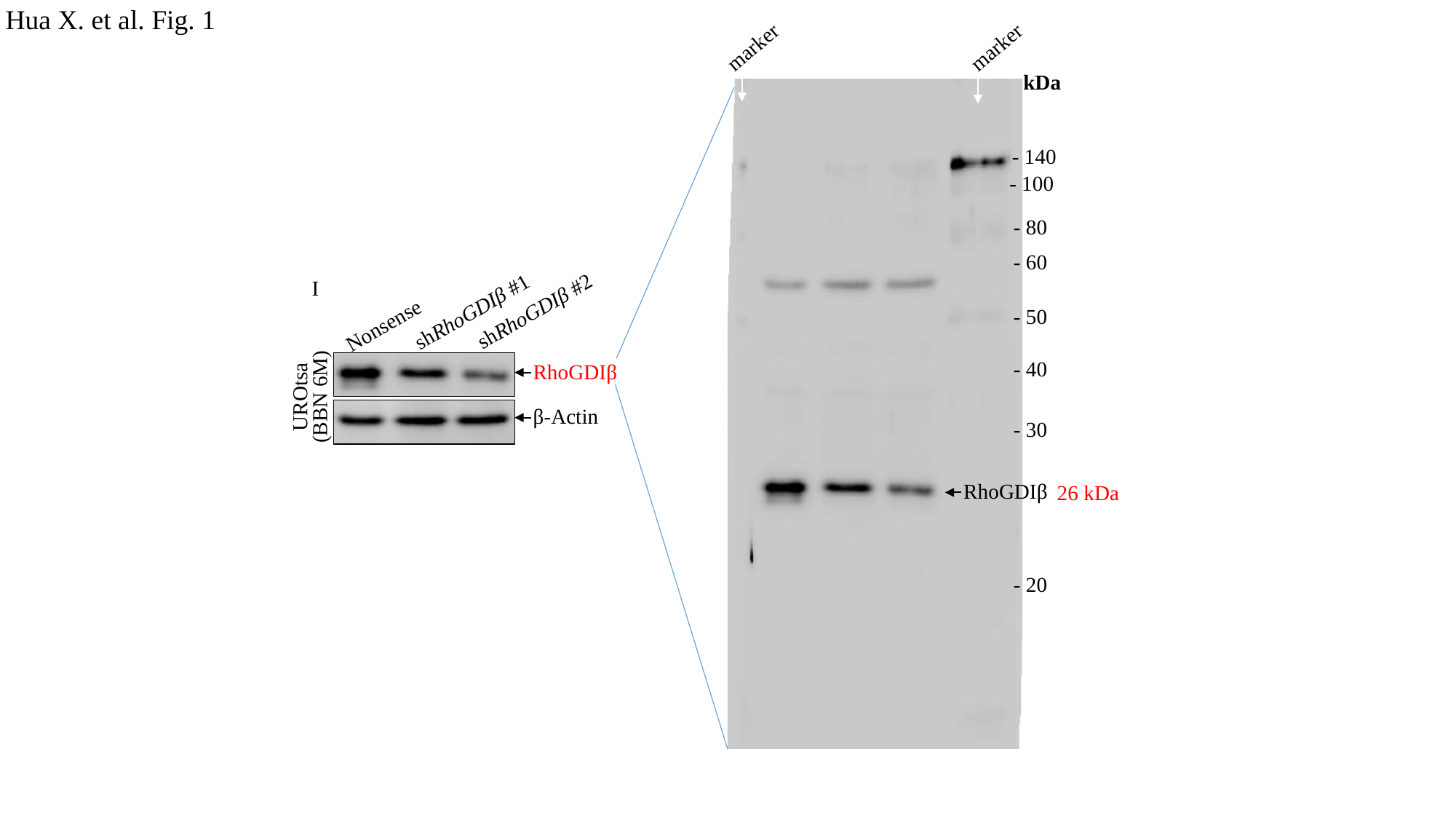

Hua X. et al. Fig. 1
marker
marker
kDa
- 140
- 100
- 80
- 60
I
shRhoGDIβ #1
shRhoGDIβ #2
- 50
Nonsense
RhoGDIβ
- 40
UROtsa
(BBN 6M)
β-Actin
- 30
RhoGDIβ
26 kDa
- 20

## Slide 6
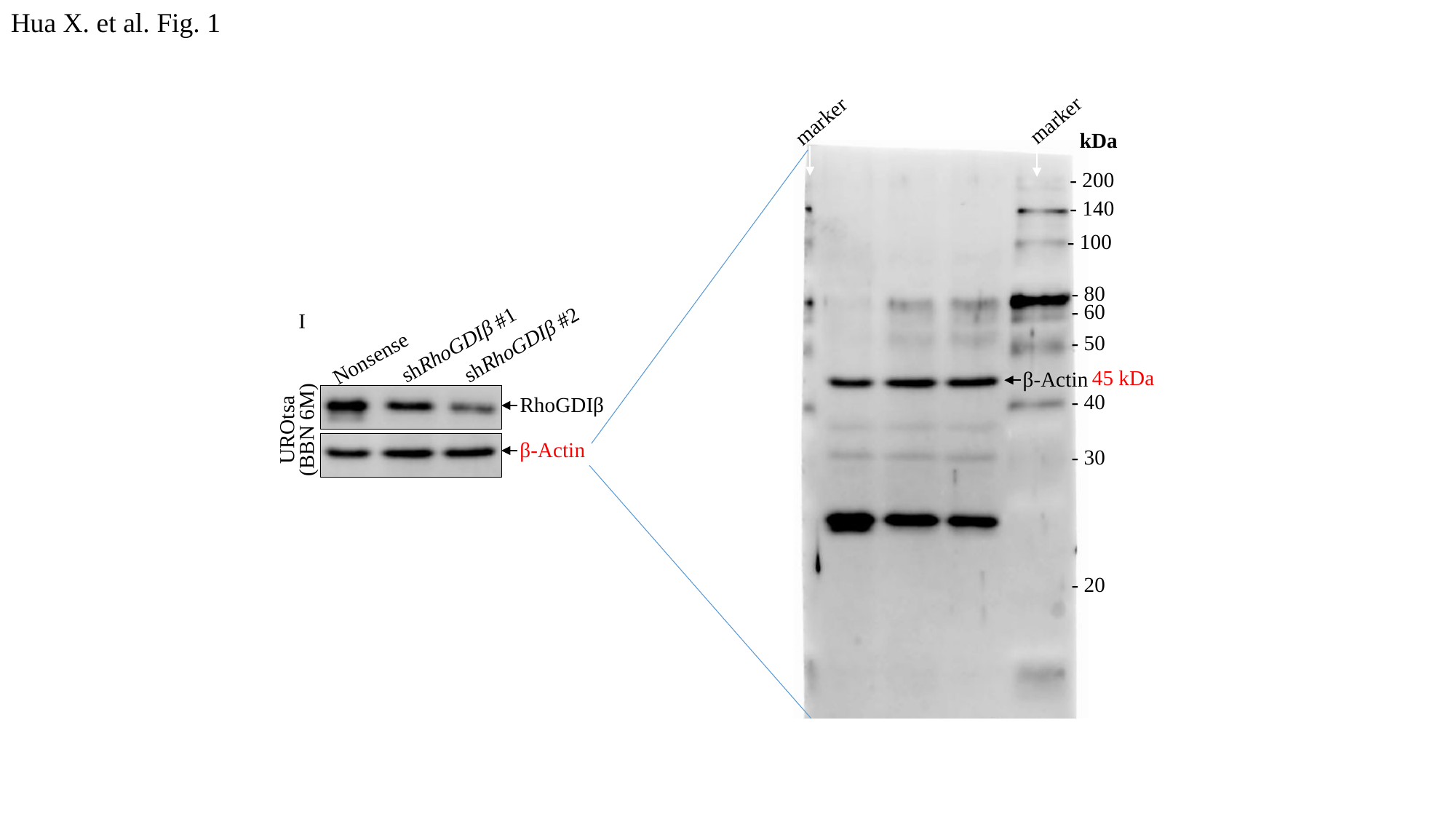

Hua X. et al. Fig. 1
marker
marker
kDa
- 200
- 140
- 100
- 80
- 60
I
shRhoGDIβ #1
shRhoGDIβ #2
- 50
Nonsense
β-Actin
45 kDa
RhoGDIβ
- 40
UROtsa
(BBN 6M)
β-Actin
- 30
- 20

## Slide 7
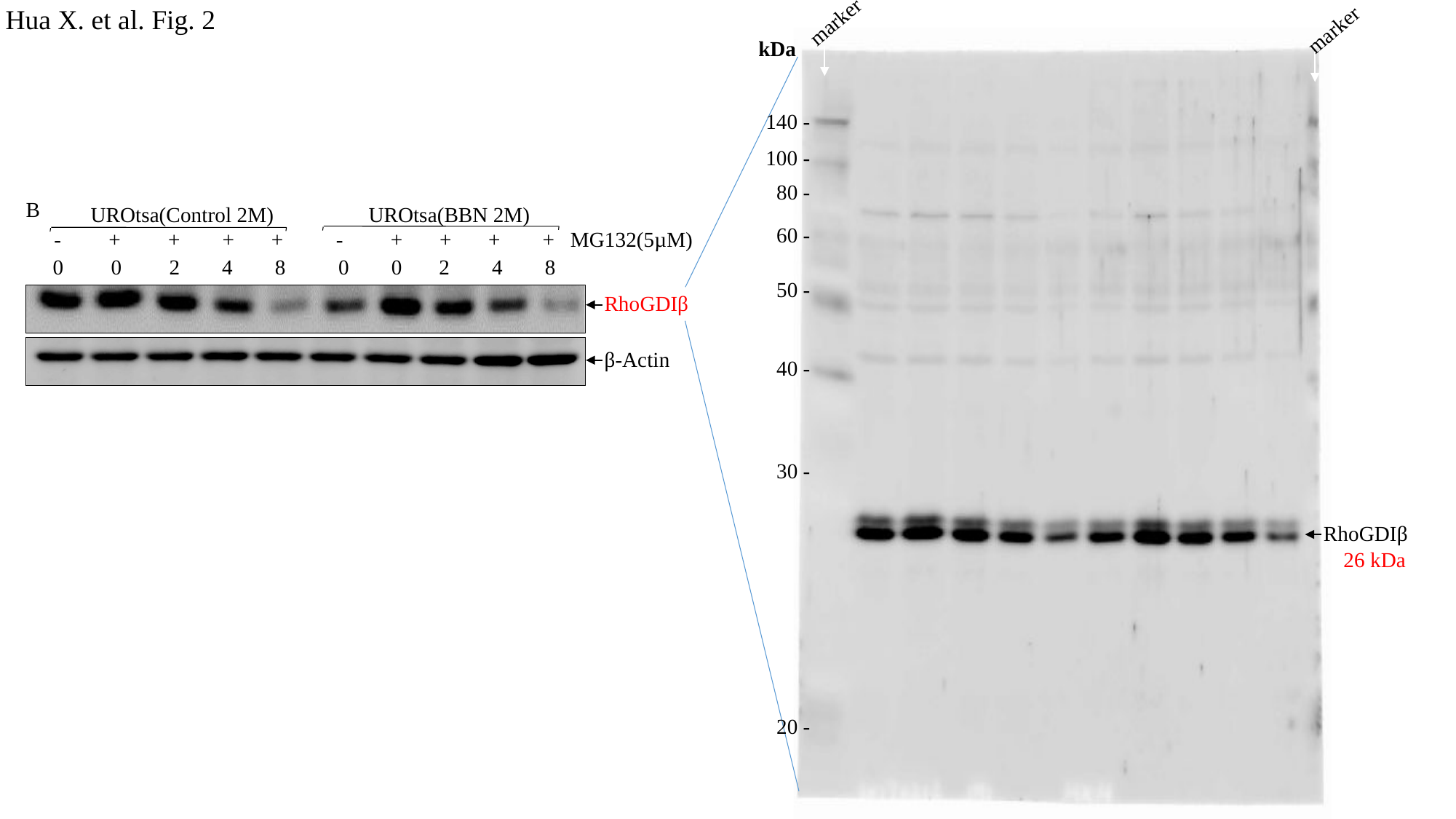

Hua X. et al. Fig. 2
marker
marker
kDa
140 -
100 -
80 -
B
UROtsa(Control 2M) UROtsa(BBN 2M)
 - + + + + - + + + + MG132(5µM)
60 -
0 0 2 4 8 0 0 2 4 8 CHX(50µg/ml)
50 -
RhoGDIβ
β-Actin
40 -
30 -
RhoGDIβ
26 kDa
20 -

## Slide 8
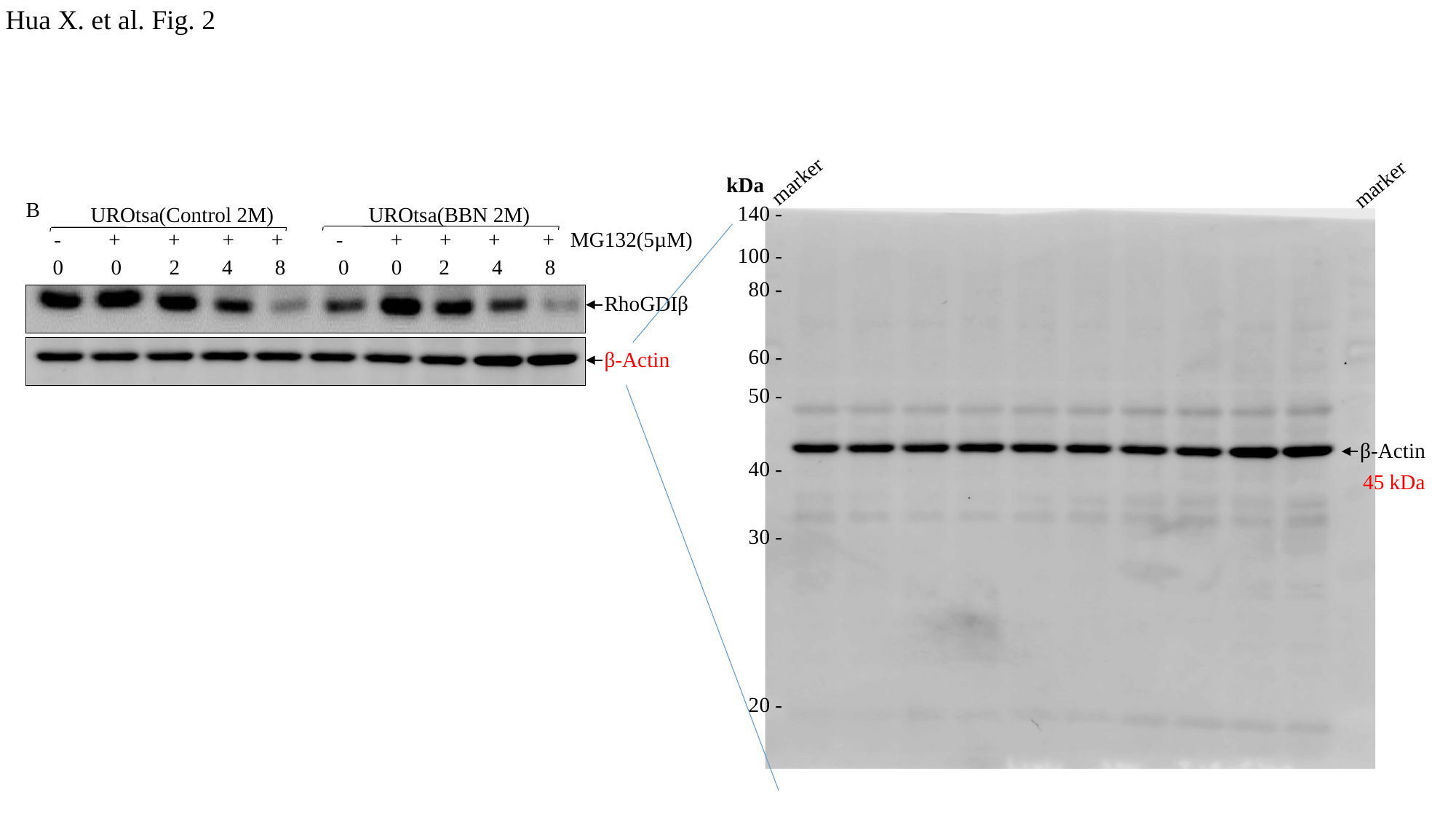

Hua X. et al. Fig. 2
marker
marker
kDa
B
UROtsa(Control 2M) UROtsa(BBN 2M)
140 -
 - + + + + - + + + + MG132(5µM)
100 -
0 0 2 4 8 0 0 2 4 8 CHX(50µg/ml)
80 -
RhoGDIβ
β-Actin
60 -
50 -
β-Actin
40 -
45 kDa
30 -
20 -

## Slide 9
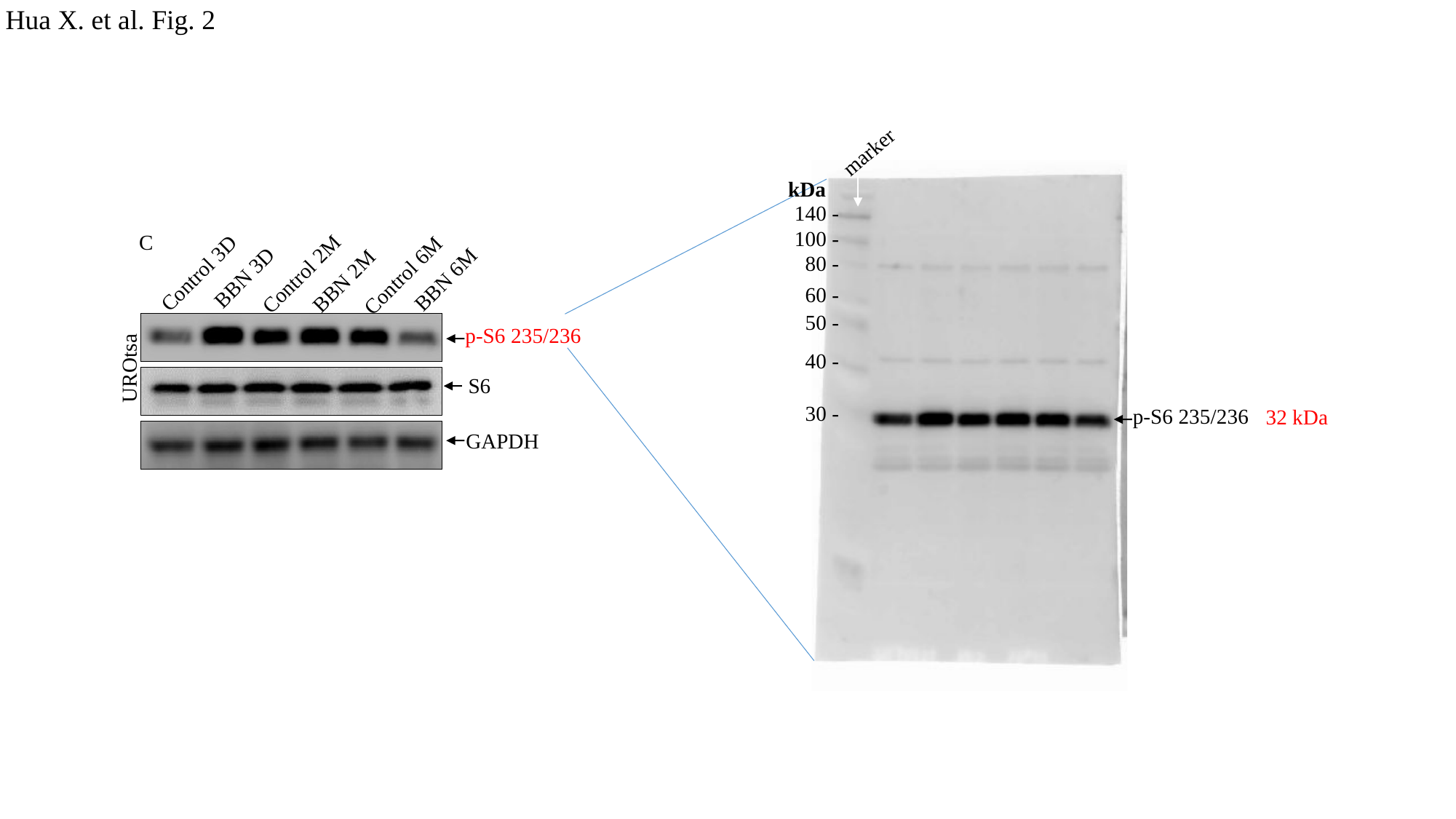

Hua X. et al. Fig. 2
marker
kDa
140 -
C
100 -
80 -
Control 2M
Control 3D
Control 6M
BBN 6M
BBN 3D
BBN 2M
60 -
50 -
p-S6 235/236
UROtsa
40 -
S6
p-S6 235/236
30 -
32 kDa
GAPDH

## Slide 10
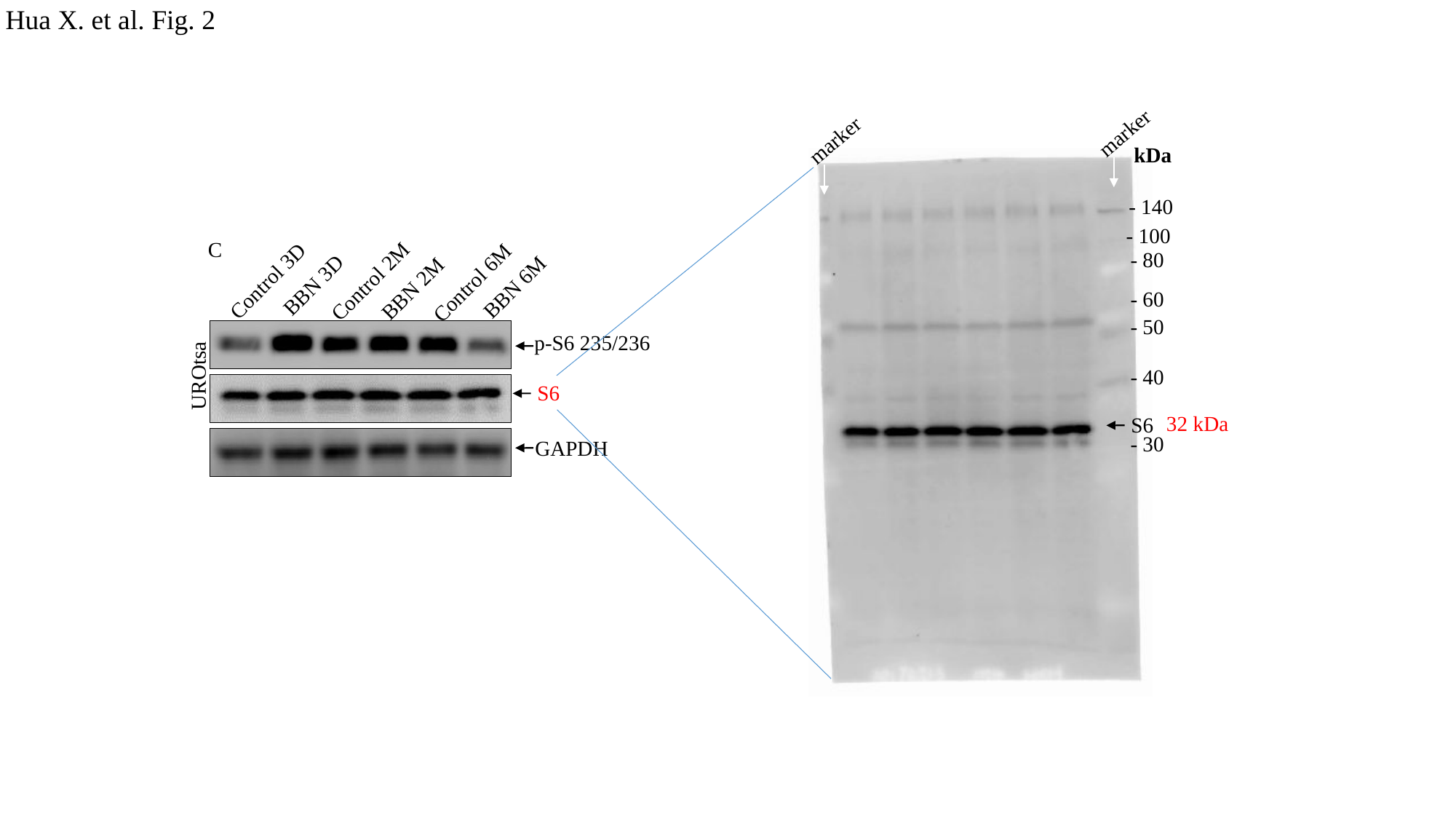

Hua X. et al. Fig. 2
marker
marker
kDa
- 140
- 100
C
- 80
Control 2M
Control 3D
Control 6M
BBN 6M
BBN 3D
BBN 2M
- 60
- 50
p-S6 235/236
UROtsa
- 40
S6
S6
32 kDa
GAPDH
- 30

## Slide 11
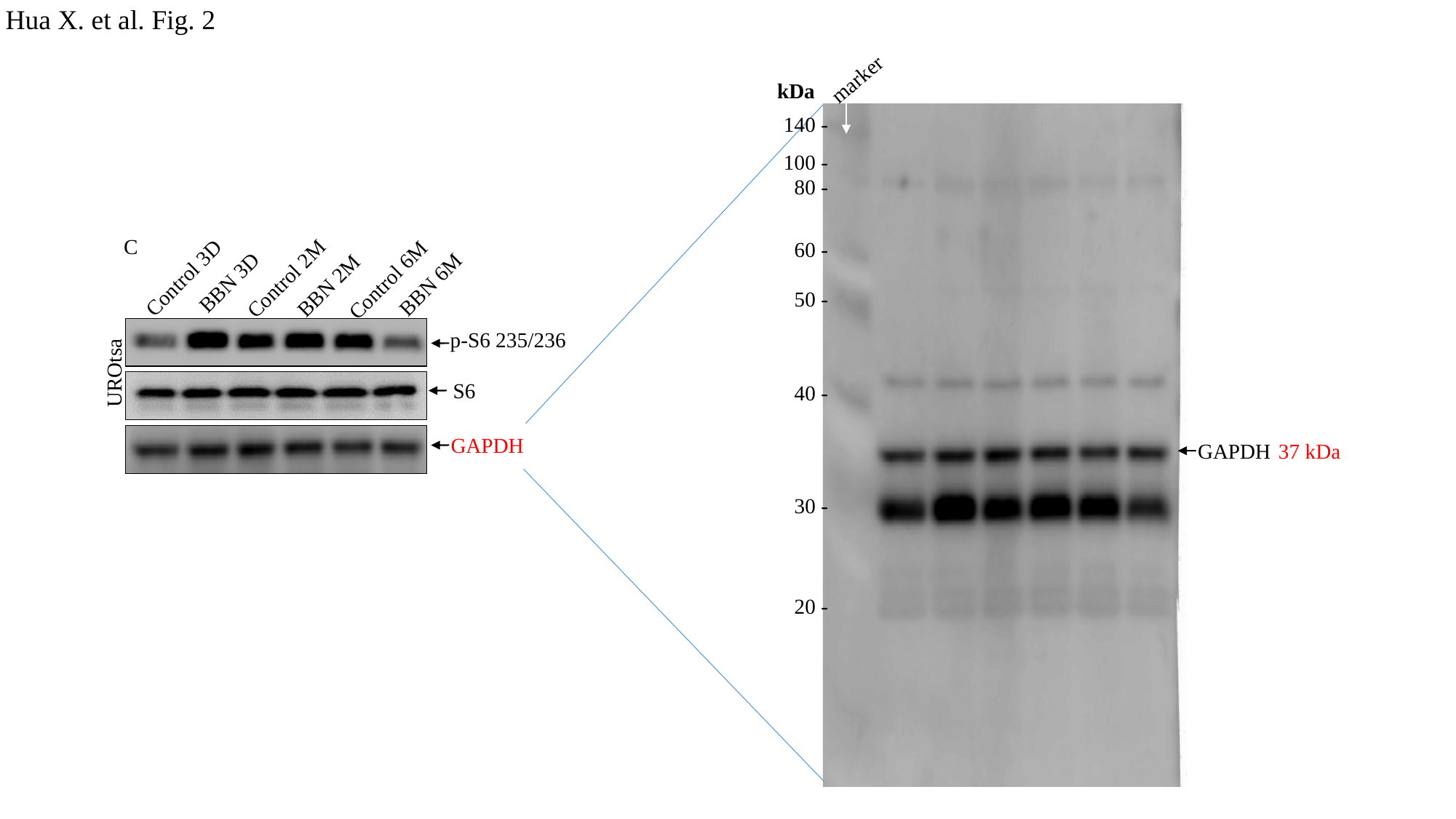

Hua X. et al. Fig. 2
marker
kDa
140 -
100 -
80 -
C
60 -
Control 2M
Control 3D
Control 6M
BBN 6M
BBN 3D
BBN 2M
50 -
p-S6 235/236
UROtsa
S6
40 -
GAPDH
GAPDH
37 kDa
30 -
20 -

## Slide 12
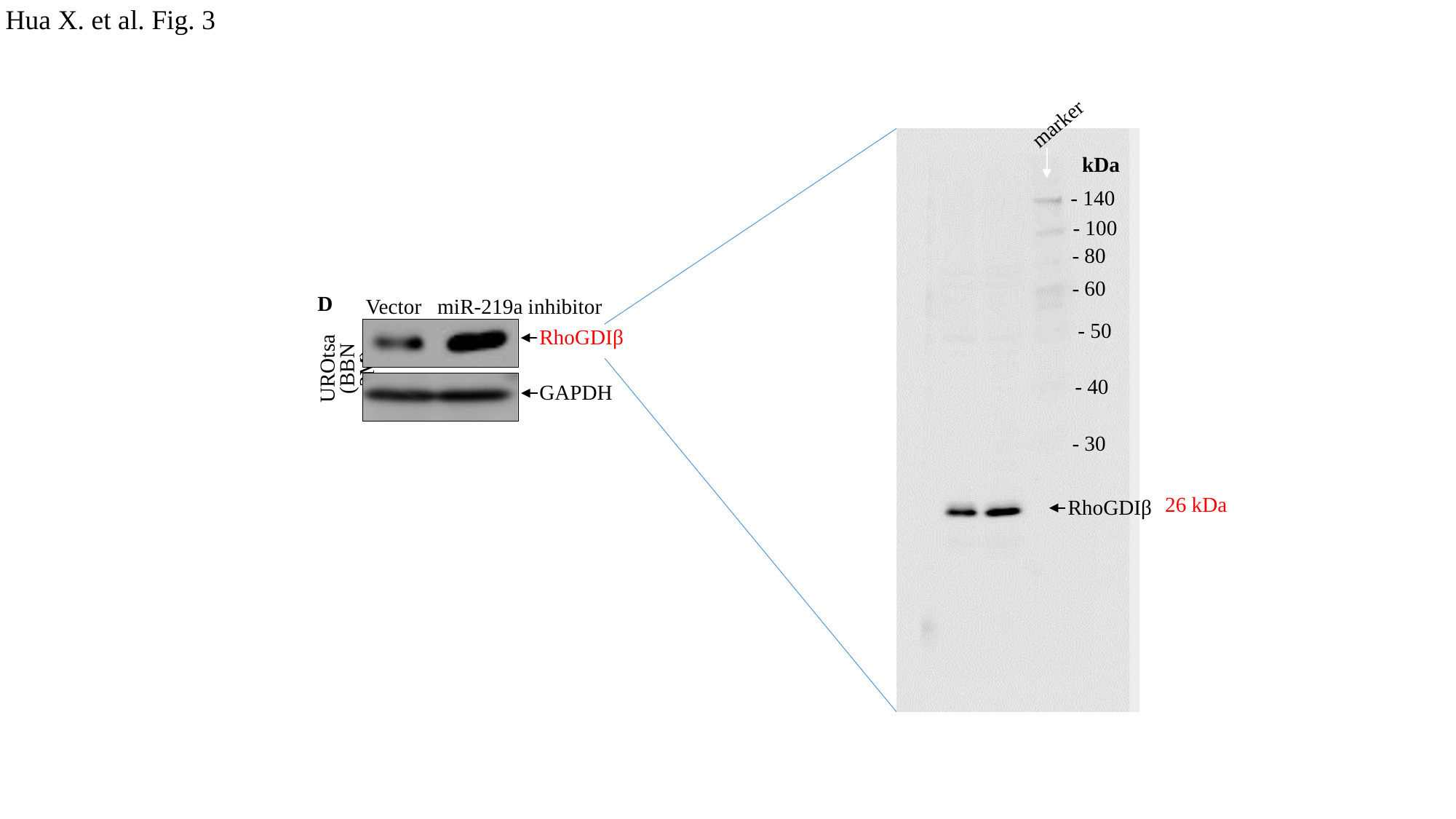

Hua X. et al. Fig. 3
marker
kDa
- 140
- 100
- 80
- 60
D
 Vector miR-219a inhibitor
RhoGDIβ
- 50
UROtsa
(BBN 2M)
GAPDH
- 40
- 30
RhoGDIβ
26 kDa

## Slide 13
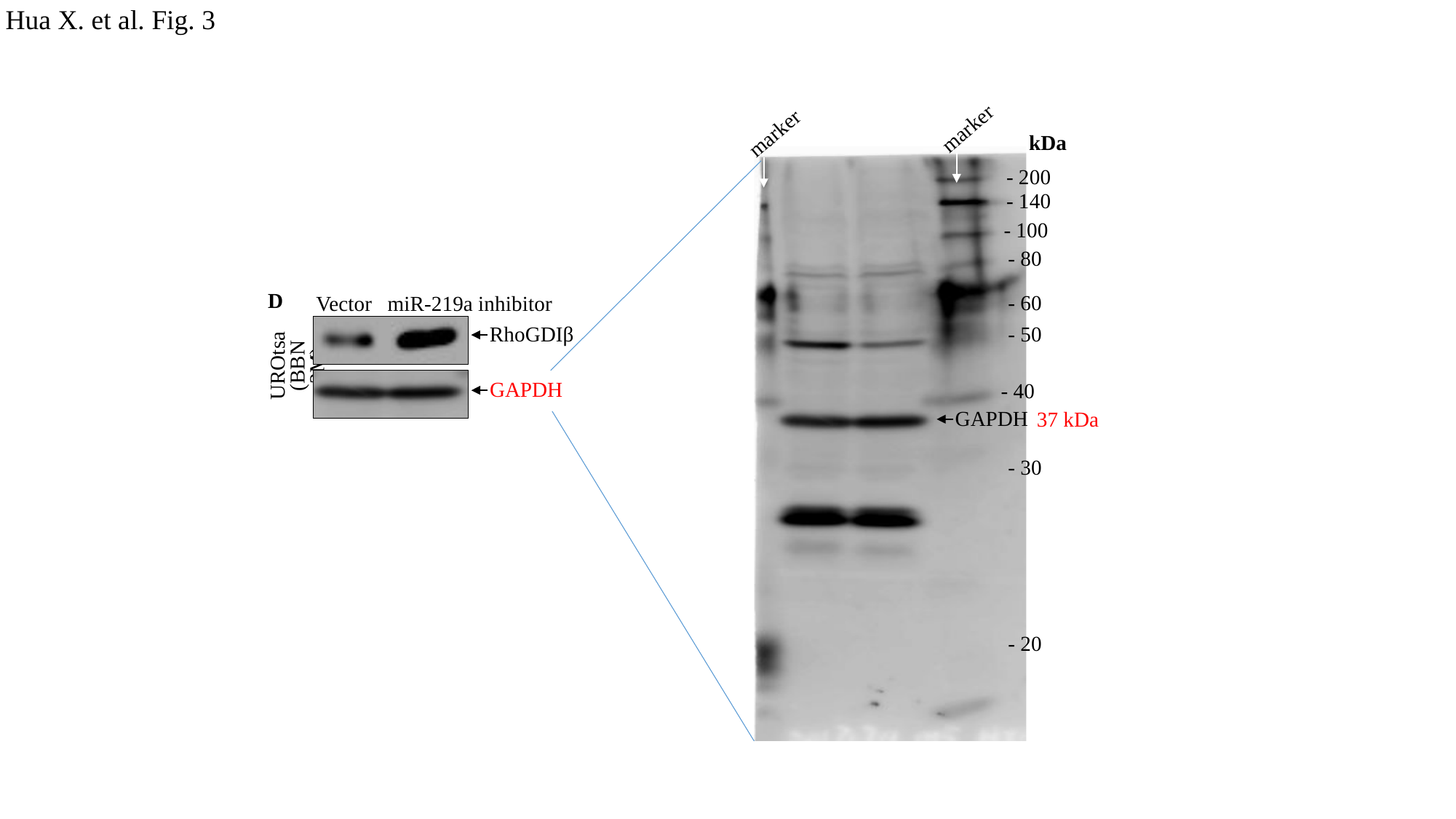

Hua X. et al. Fig. 3
marker
marker
kDa
- 200
- 140
- 100
- 80
D
 Vector miR-219a inhibitor
- 60
RhoGDIβ
- 50
UROtsa
(BBN 2M)
GAPDH
- 40
GAPDH
37 kDa
- 30
- 20

## Slide 14
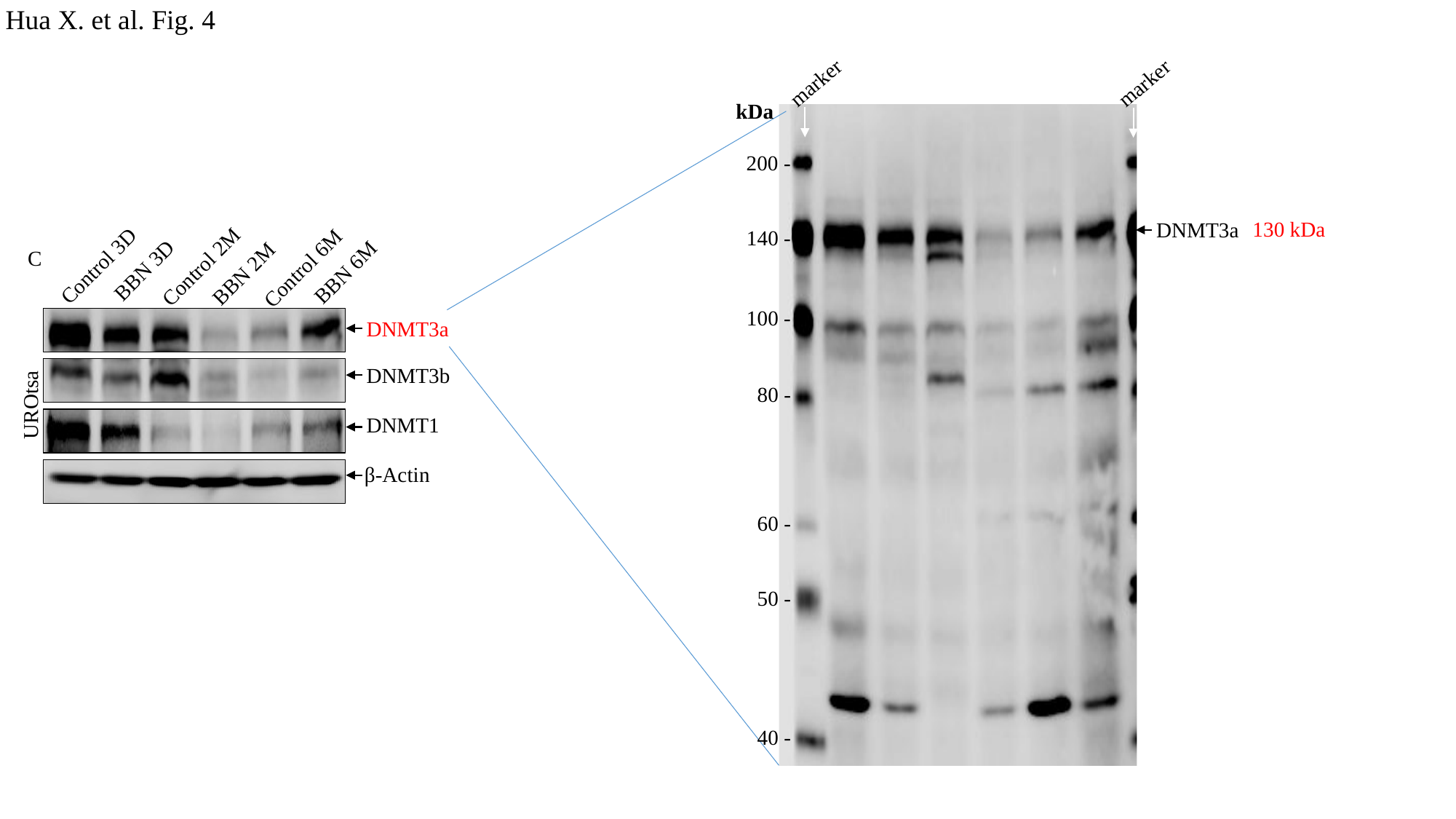

Hua X. et al. Fig. 4
marker
marker
kDa
200 -
DNMT3a
130 kDa
140 -
C
Control 2M
Control 3D
Control 6M
BBN 6M
BBN 3D
BBN 2M
100 -
DNMT3a
DNMT3b
UROtsa
80 -
DNMT1
β-Actin
60 -
50 -
40 -

## Slide 15
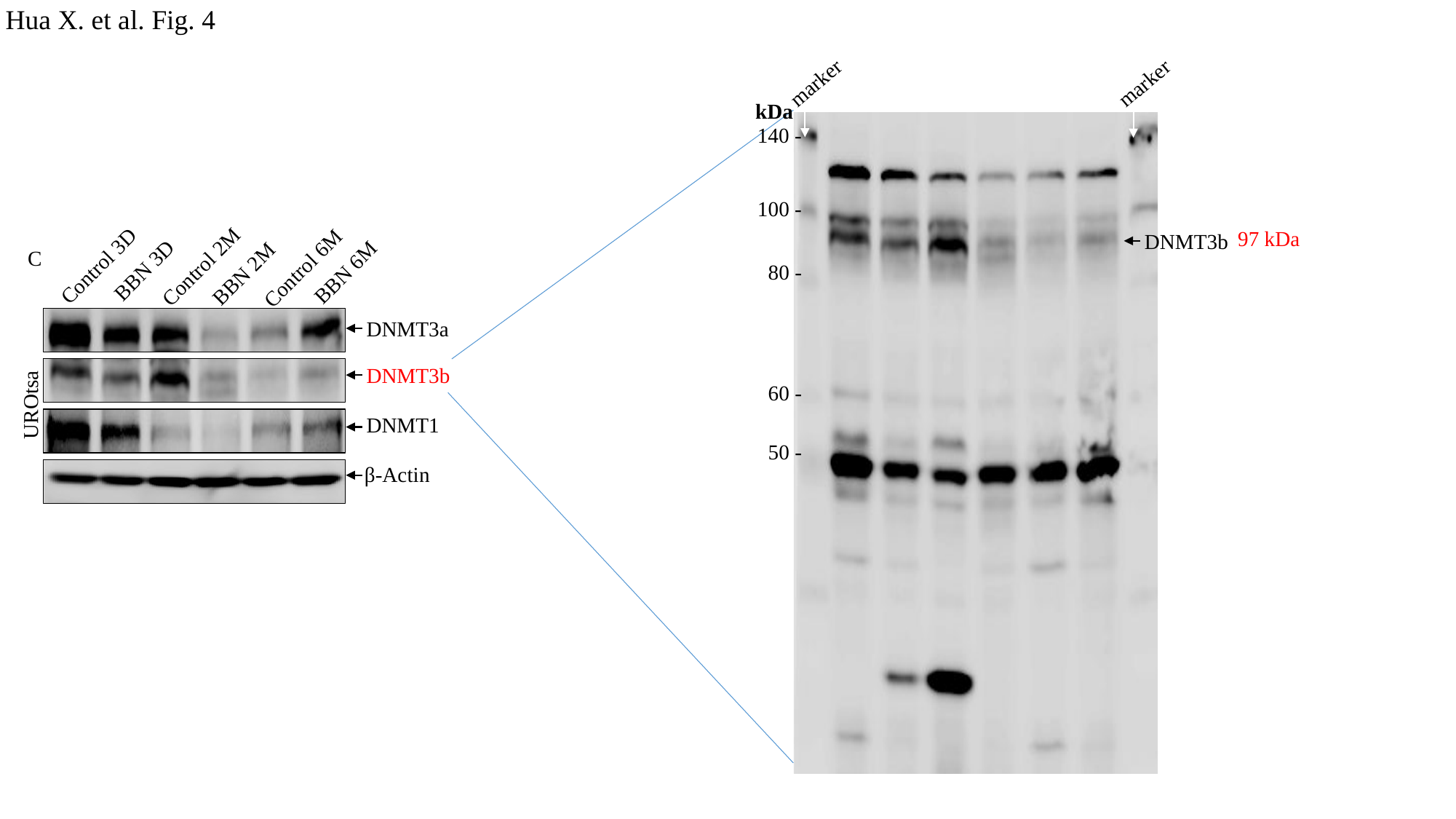

Hua X. et al. Fig. 4
marker
marker
kDa
140 -
100 -
DNMT3b
97 kDa
C
Control 2M
Control 3D
Control 6M
BBN 6M
BBN 3D
BBN 2M
80 -
DNMT3a
DNMT3b
60 -
UROtsa
DNMT1
50 -
β-Actin

## Slide 16
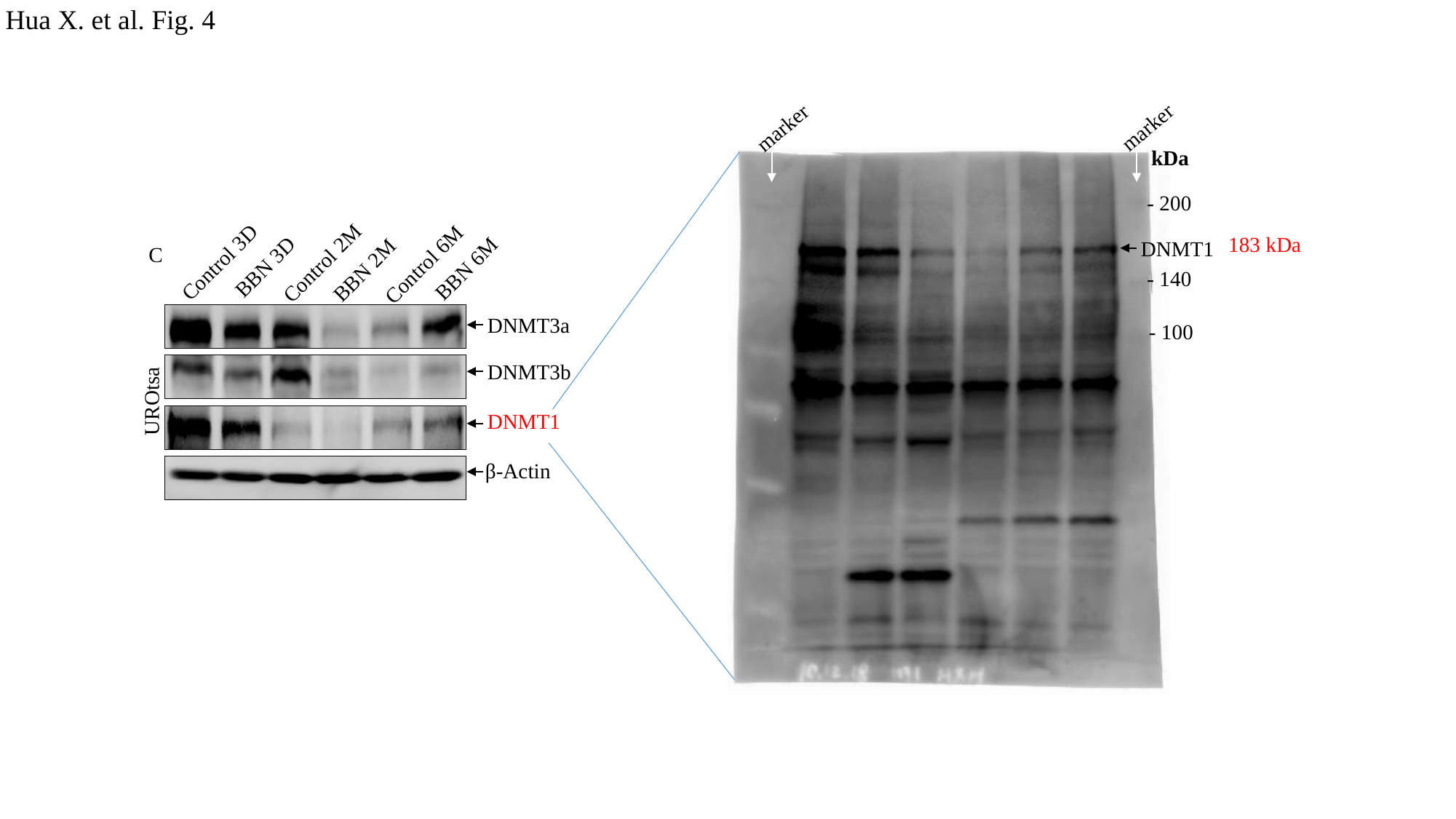

Hua X. et al. Fig. 4
marker
marker
kDa
- 200
DNMT1
183 kDa
C
Control 2M
Control 3D
Control 6M
BBN 6M
BBN 3D
BBN 2M
- 140
DNMT3a
- 100
DNMT3b
UROtsa
DNMT1
β-Actin

## Slide 17
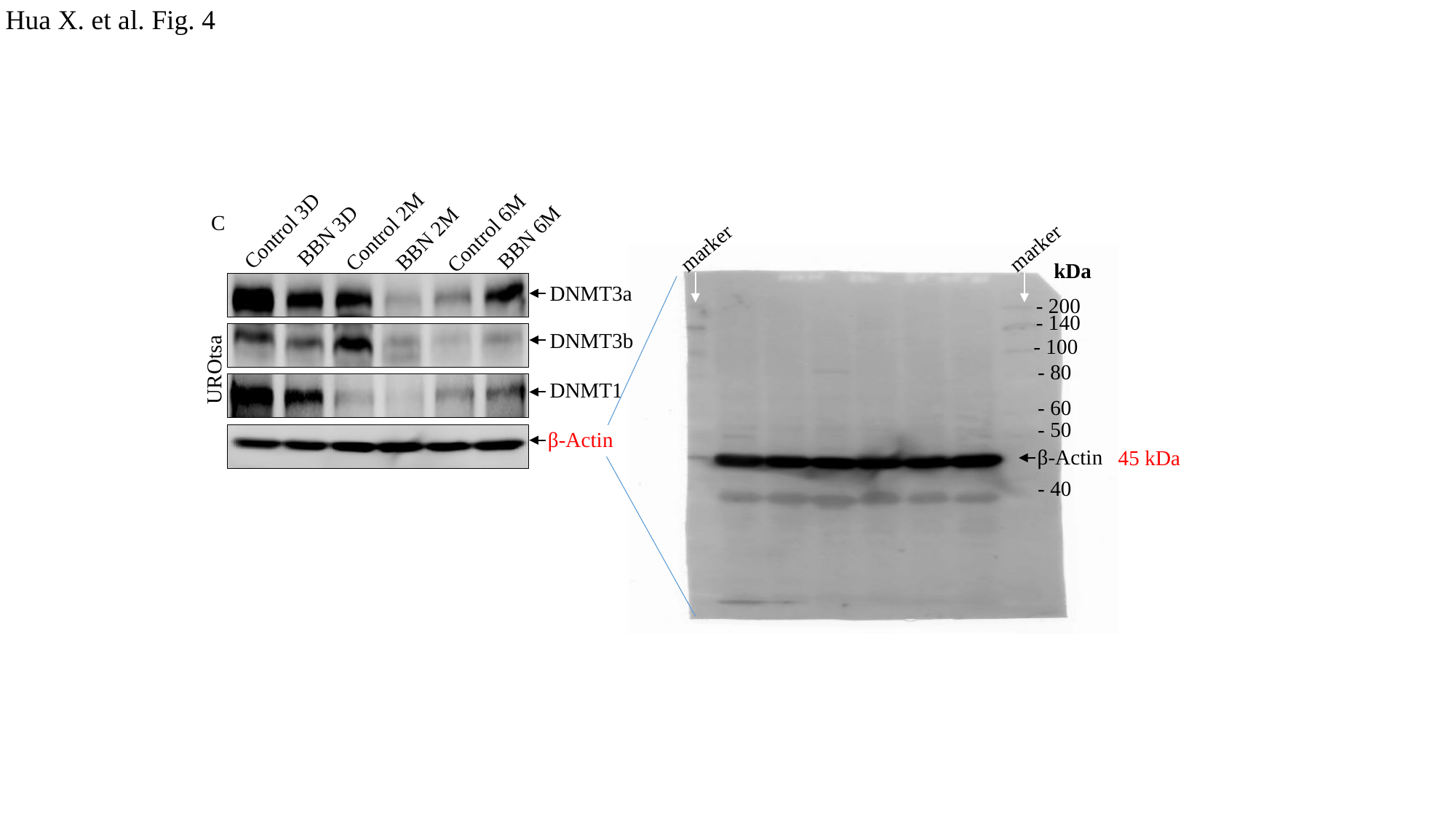

Hua X. et al. Fig. 4
C
Control 2M
Control 3D
Control 6M
BBN 6M
BBN 3D
BBN 2M
marker
marker
kDa
DNMT3a
- 200
- 140
DNMT3b
- 100
UROtsa
- 80
DNMT1
- 60
- 50
β-Actin
β-Actin
45 kDa
- 40

## Slide 18
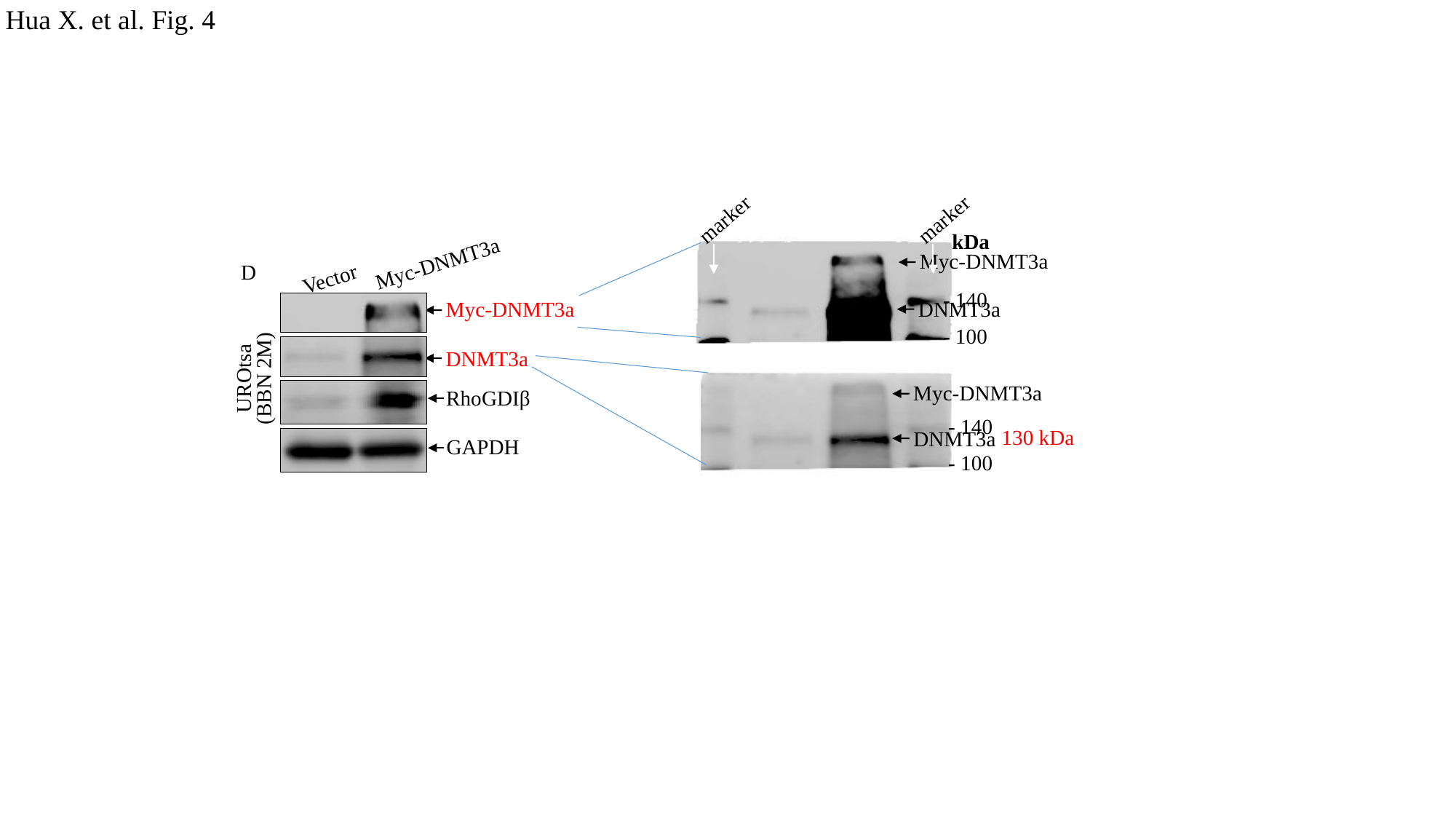

Hua X. et al. Fig. 4
marker
marker
kDa
Myc-DNMT3a
Myc-DNMT3a
D
 Vector
- 140
Myc-DNMT3a
DNMT3a
DNMT3a
- 100
UROtsa
(BBN 2M)
Myc-DNMT3a
RhoGDIβ
- 140
DNMT3a
130 kDa
GAPDH
- 100

## Slide 19
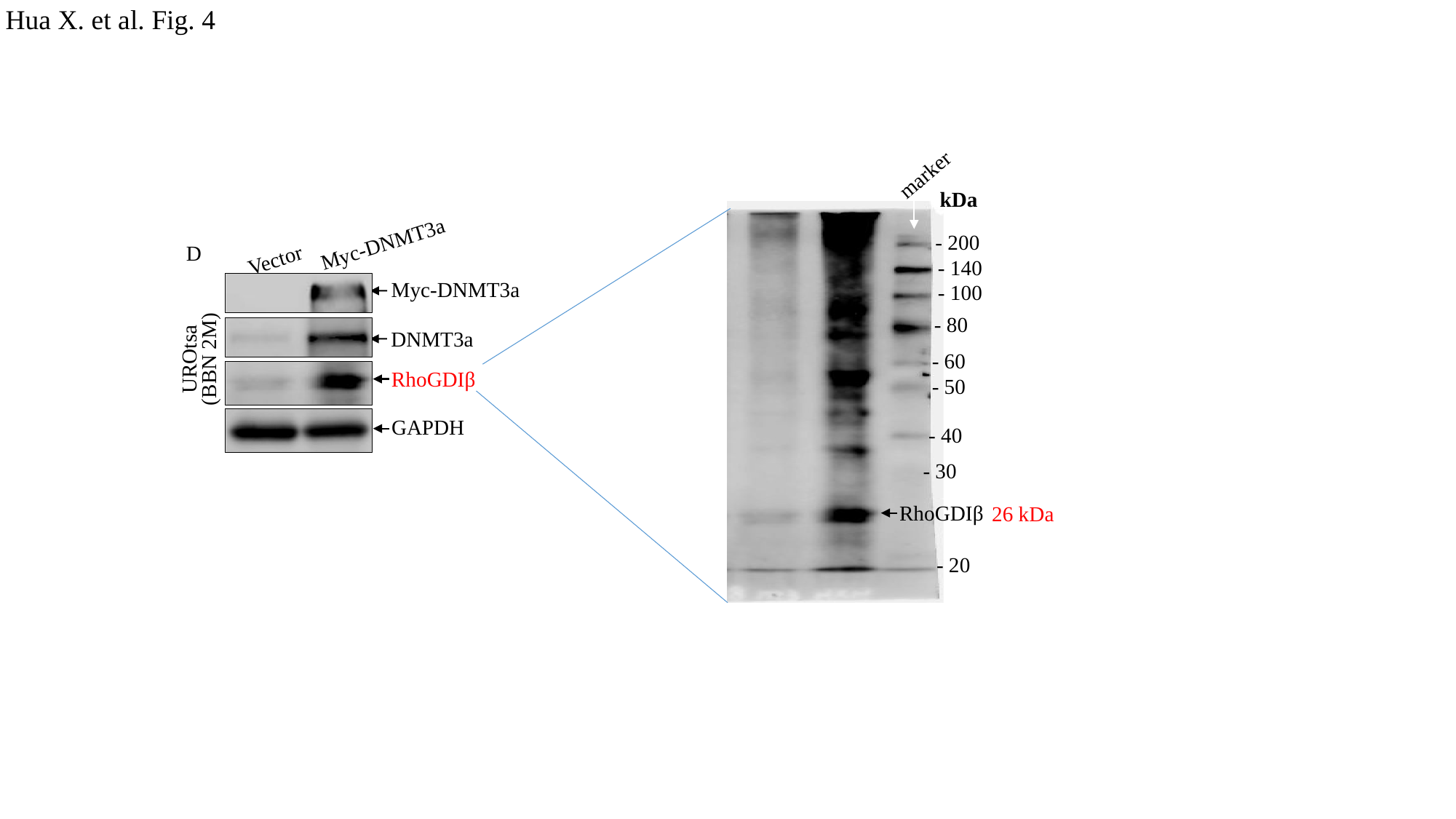

Hua X. et al. Fig. 4
marker
kDa
Myc-DNMT3a
- 200
D
 Vector
- 140
Myc-DNMT3a
DNMT3a
- 100
- 80
UROtsa
(BBN 2M)
- 60
RhoGDIβ
- 50
GAPDH
- 40
- 30
RhoGDIβ
26 kDa
- 20

## Slide 20
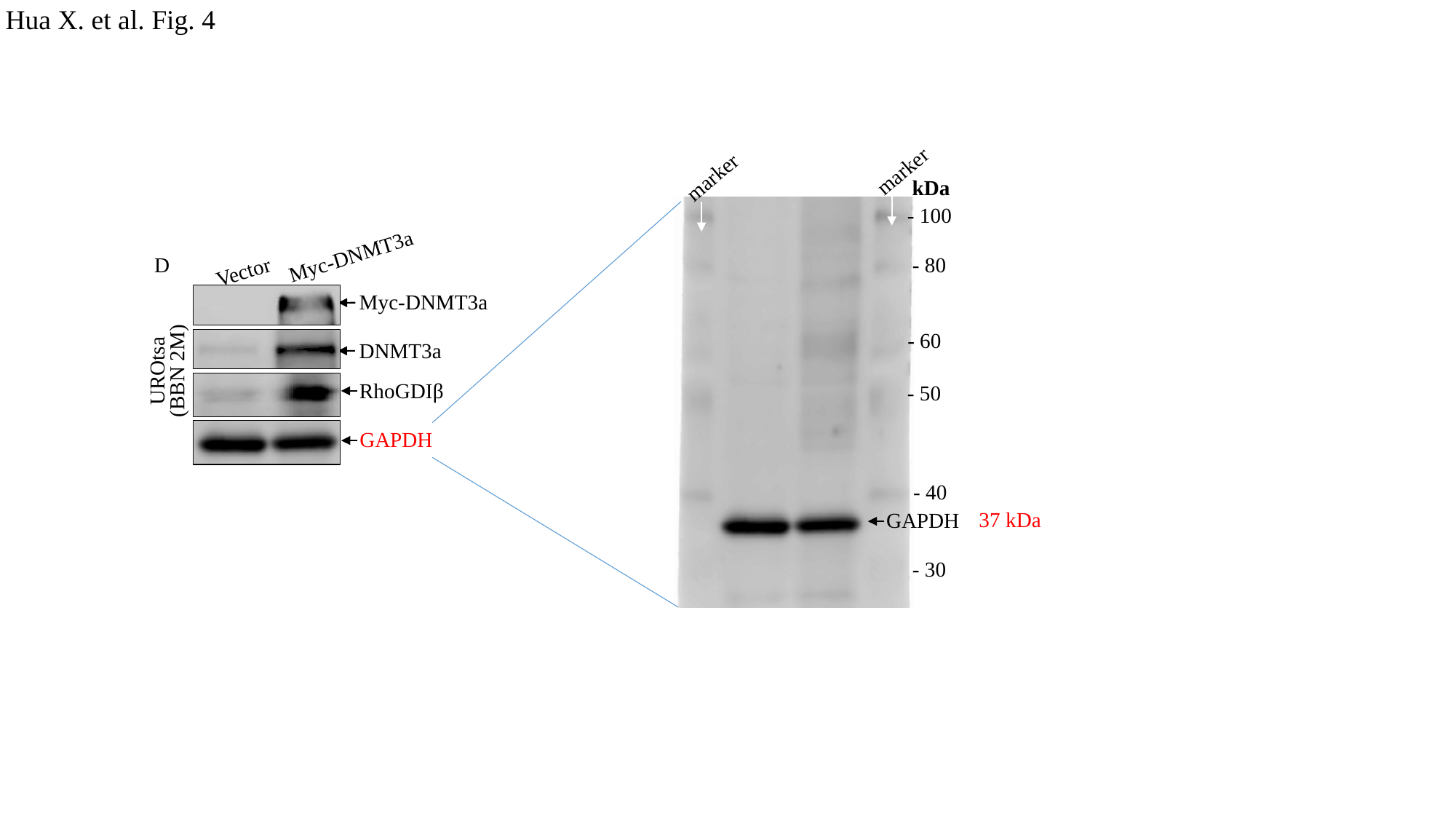

Hua X. et al. Fig. 4
marker
marker
kDa
- 100
Myc-DNMT3a
D
- 80
 Vector
Myc-DNMT3a
DNMT3a
- 60
UROtsa
(BBN 2M)
RhoGDIβ
- 50
GAPDH
- 40
GAPDH
37 kDa
- 30

## Slide 21
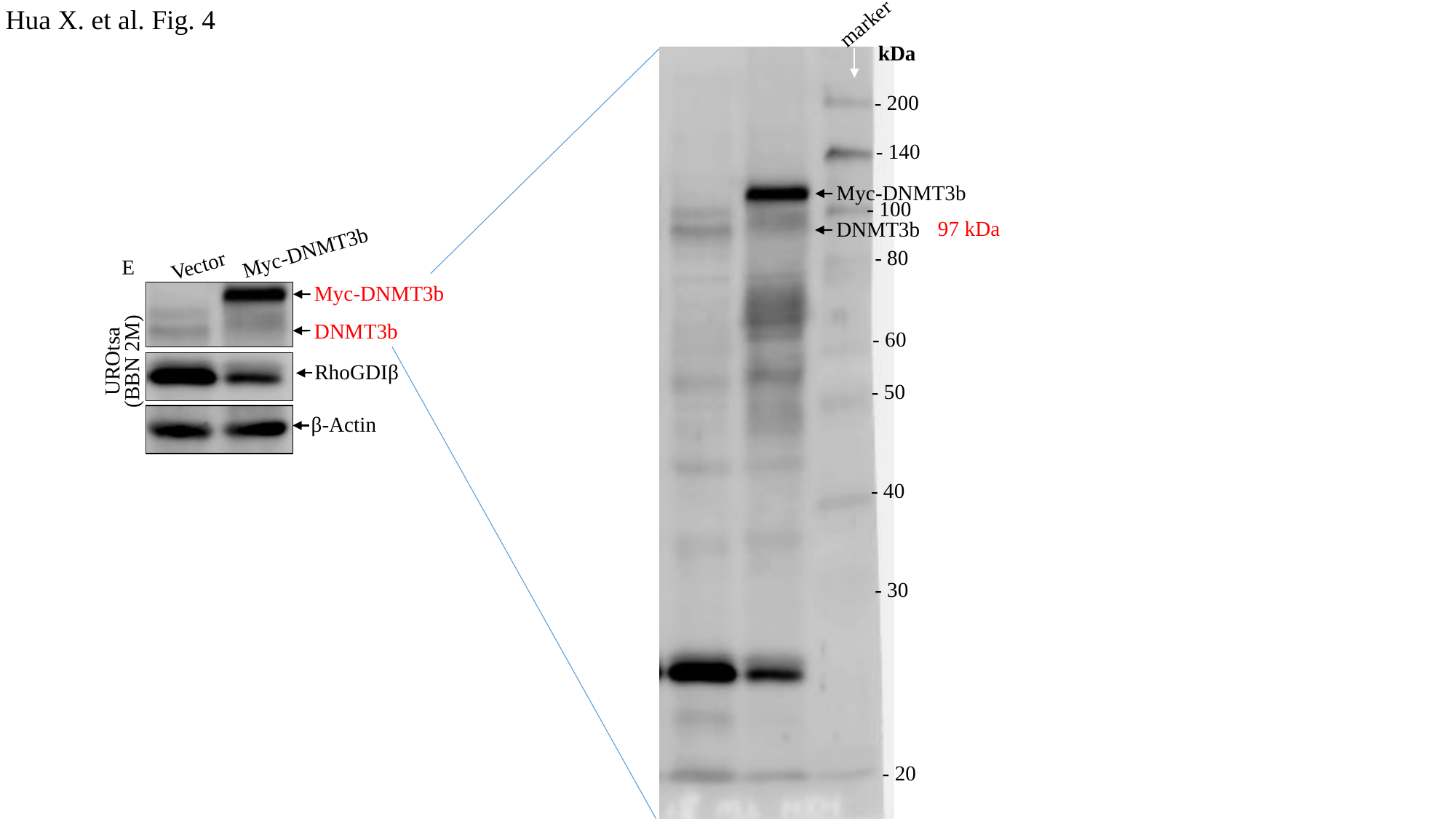

Hua X. et al. Fig. 4
marker
kDa
- 200
- 140
Myc-DNMT3b
DNMT3b
- 100
97 kDa
Myc-DNMT3b
- 80
 Vector
E
Myc-DNMT3b
DNMT3b
- 60
UROtsa
(BBN 2M)
RhoGDIβ
- 50
β-Actin
- 40
- 30
- 20

## Slide 22
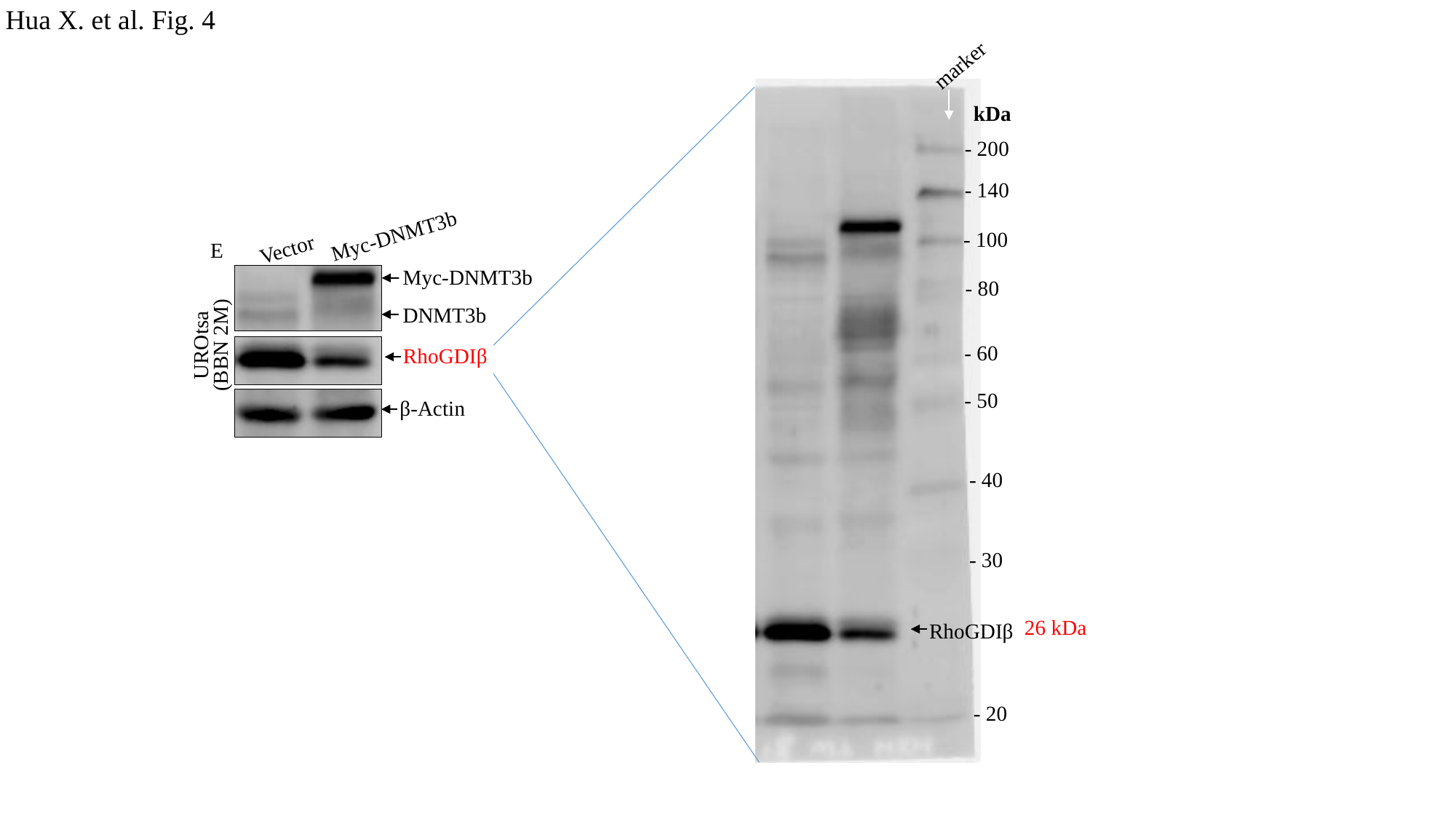

Hua X. et al. Fig. 4
marker
kDa
- 200
- 140
Myc-DNMT3b
- 100
 Vector
E
Myc-DNMT3b
DNMT3b
- 80
UROtsa
(BBN 2M)
RhoGDIβ
- 60
- 50
β-Actin
- 40
- 30
RhoGDIβ
26 kDa
- 20

## Slide 23
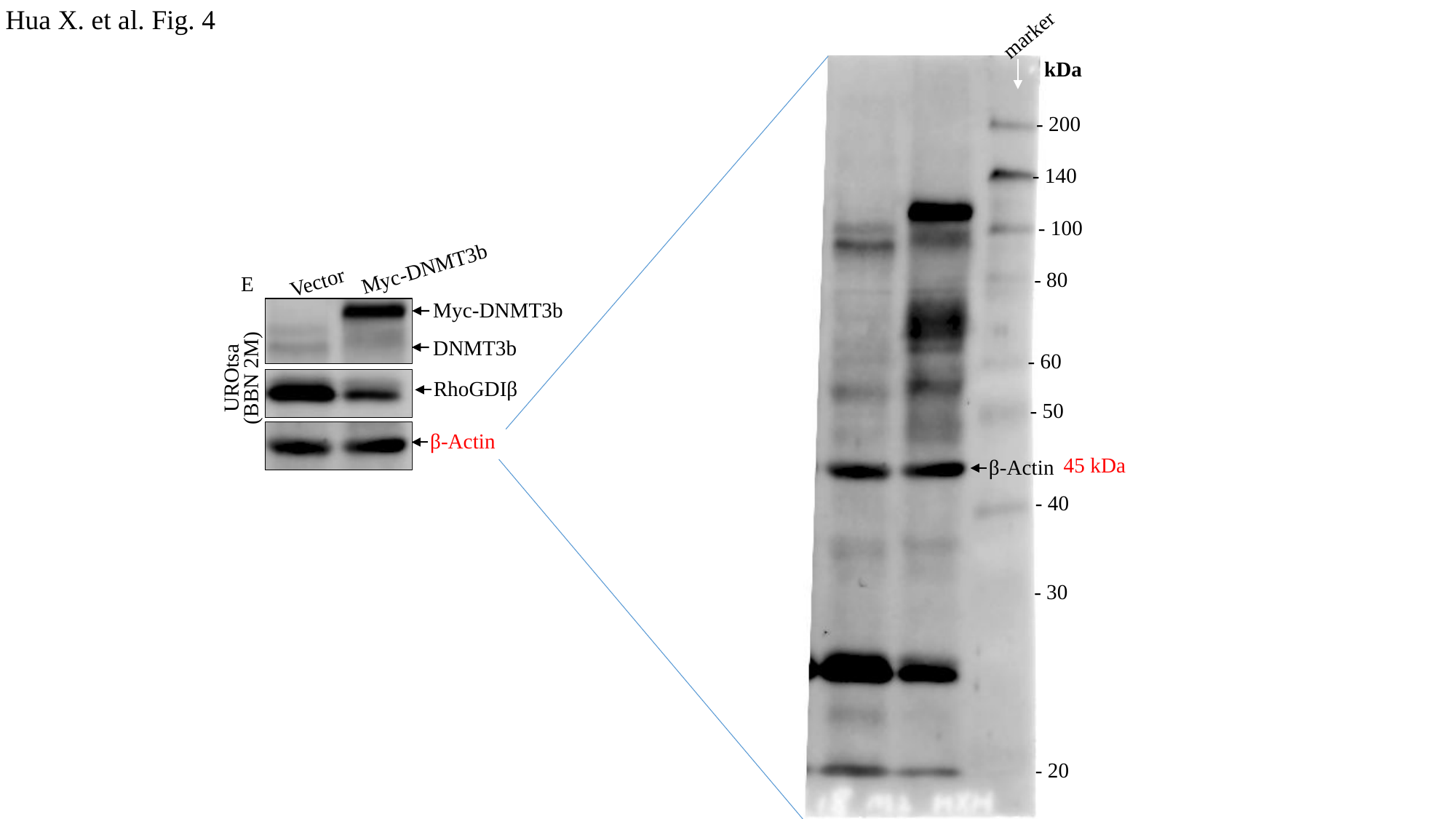

Hua X. et al. Fig. 4
marker
kDa
- 200
- 140
- 100
Myc-DNMT3b
 Vector
E
- 80
Myc-DNMT3b
DNMT3b
- 60
UROtsa
(BBN 2M)
RhoGDIβ
- 50
β-Actin
β-Actin
45 kDa
- 40
- 30
- 20

## Slide 24
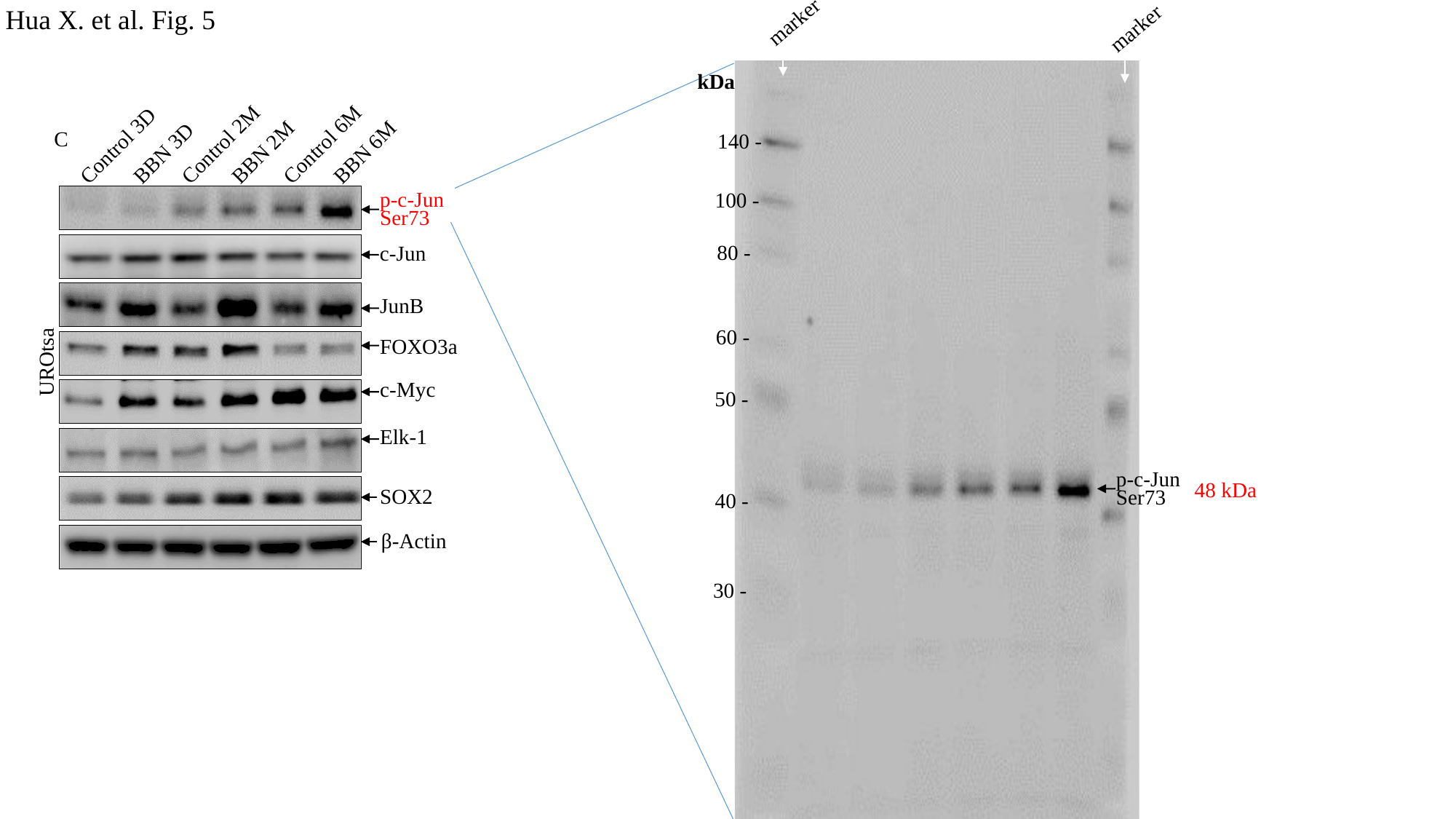

Hua X. et al. Fig. 5
marker
marker
kDa
Control 6M
C
Control 2M
Control 3D
BBN 3D
BBN 2M
140 -
BBN 6M
100 -
p-c-Jun Ser73
c-Jun
80 -
JunB
60 -
FOXO3a
UROtsa
c-Myc
50 -
Elk-1
p-c-Jun Ser73
48 kDa
SOX2
40 -
β-Actin
30 -

## Slide 25
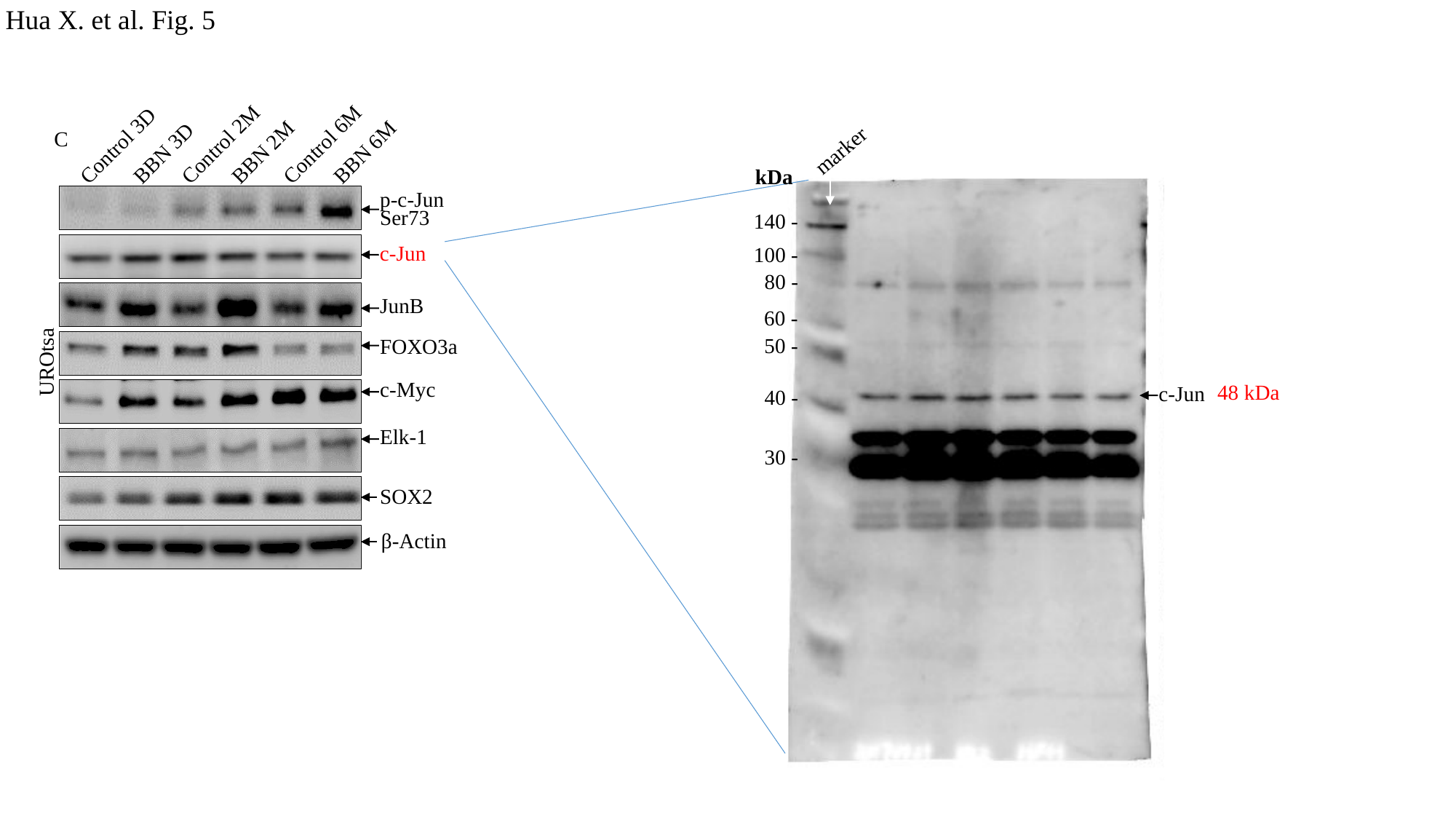

Hua X. et al. Fig. 5
Control 6M
C
Control 2M
Control 3D
BBN 3D
BBN 2M
BBN 6M
marker
kDa
p-c-Jun Ser73
140 -
c-Jun
100 -
80 -
JunB
60 -
FOXO3a
50 -
UROtsa
c-Myc
c-Jun
48 kDa
40 -
Elk-1
30 -
SOX2
β-Actin

## Slide 26
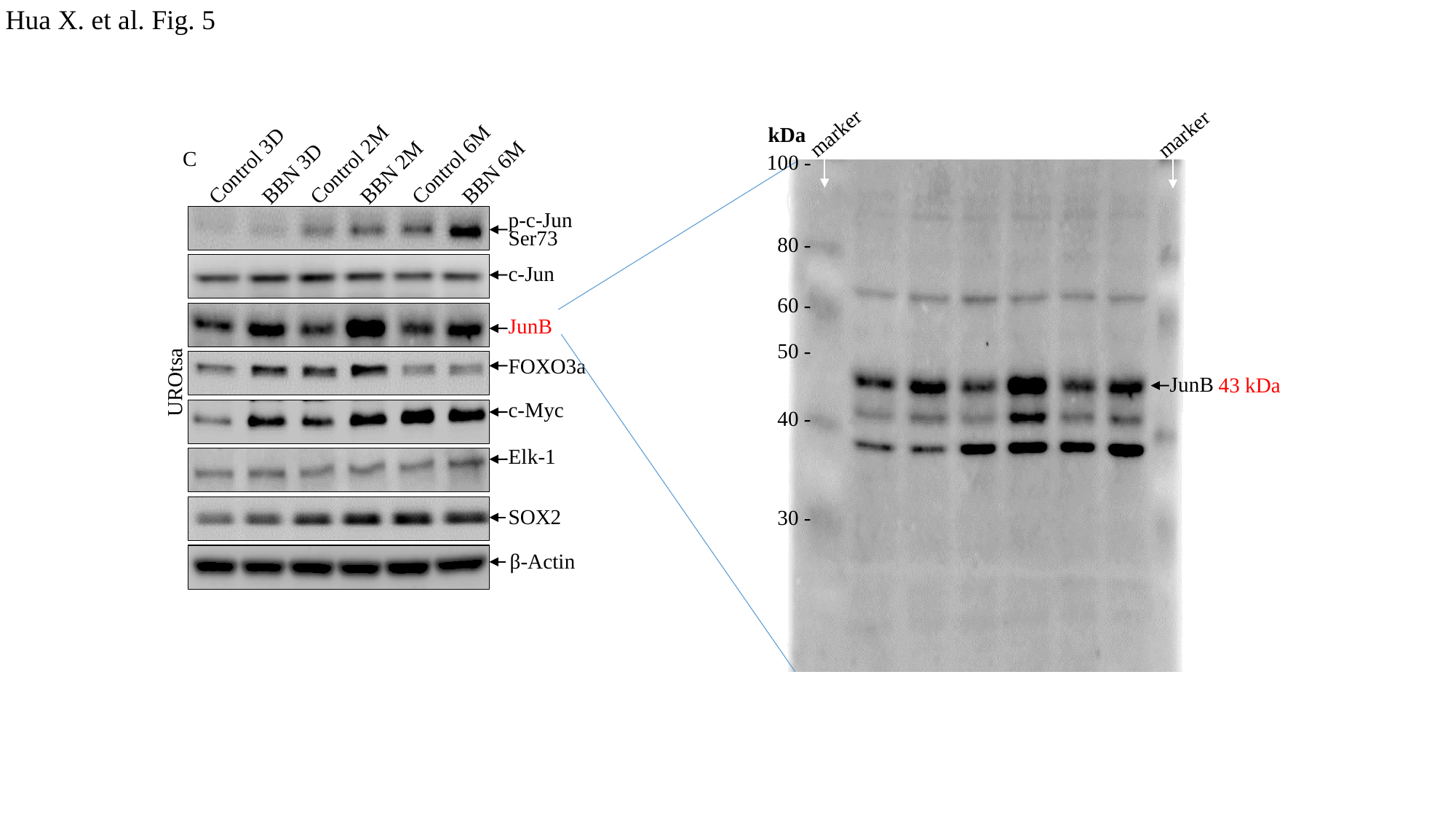

Hua X. et al. Fig. 5
marker
marker
kDa
Control 6M
C
Control 2M
Control 3D
BBN 3D
BBN 2M
BBN 6M
100 -
p-c-Jun Ser73
80 -
c-Jun
60 -
JunB
50 -
FOXO3a
UROtsa
JunB
43 kDa
c-Myc
40 -
Elk-1
SOX2
30 -
β-Actin

## Slide 27
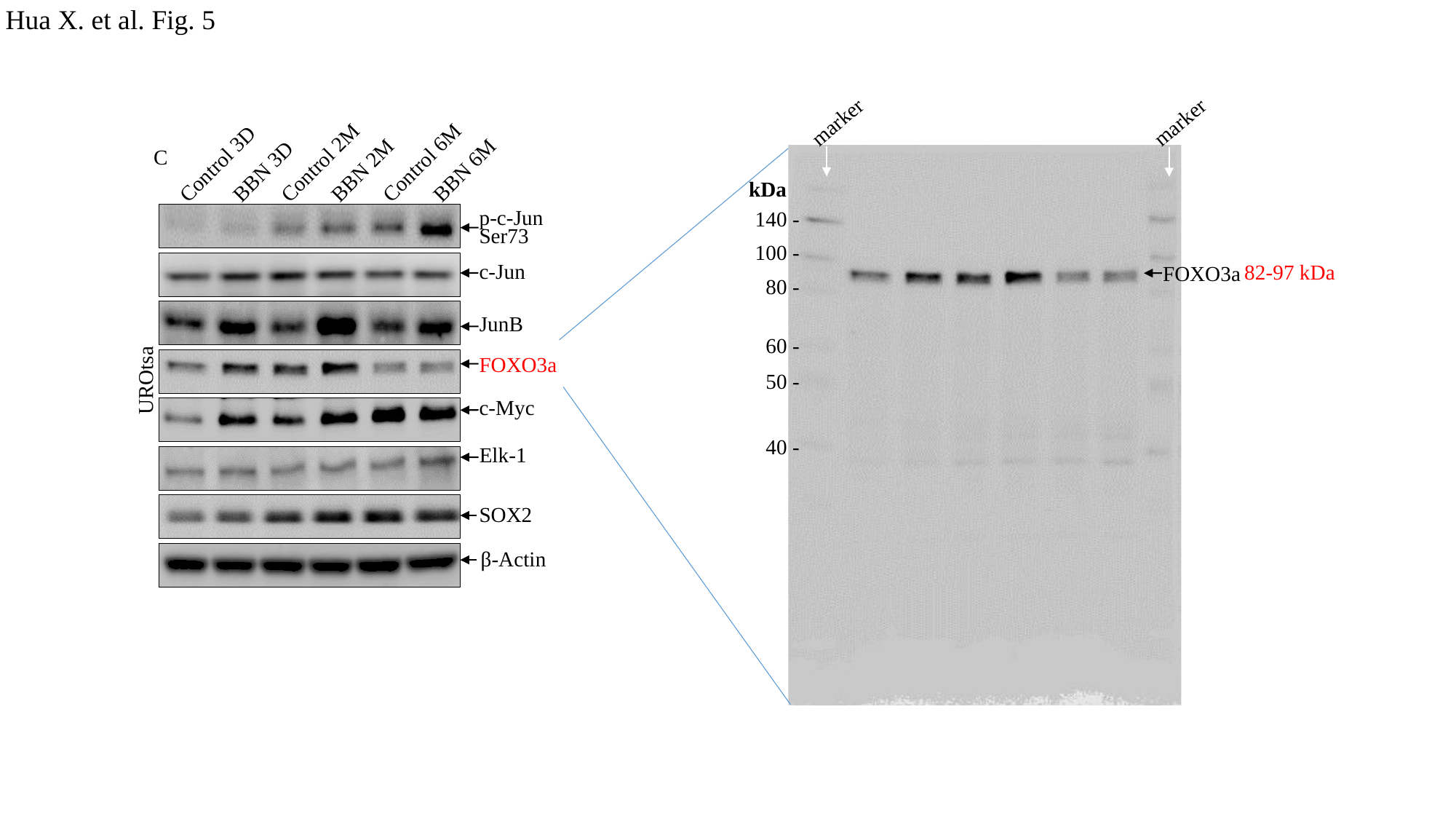

Hua X. et al. Fig. 5
marker
marker
Control 6M
C
Control 2M
Control 3D
BBN 3D
BBN 2M
BBN 6M
kDa
p-c-Jun Ser73
140 -
100 -
c-Jun
FOXO3a
82-97 kDa
80 -
JunB
60 -
FOXO3a
UROtsa
50 -
c-Myc
40 -
Elk-1
SOX2
β-Actin

## Slide 28
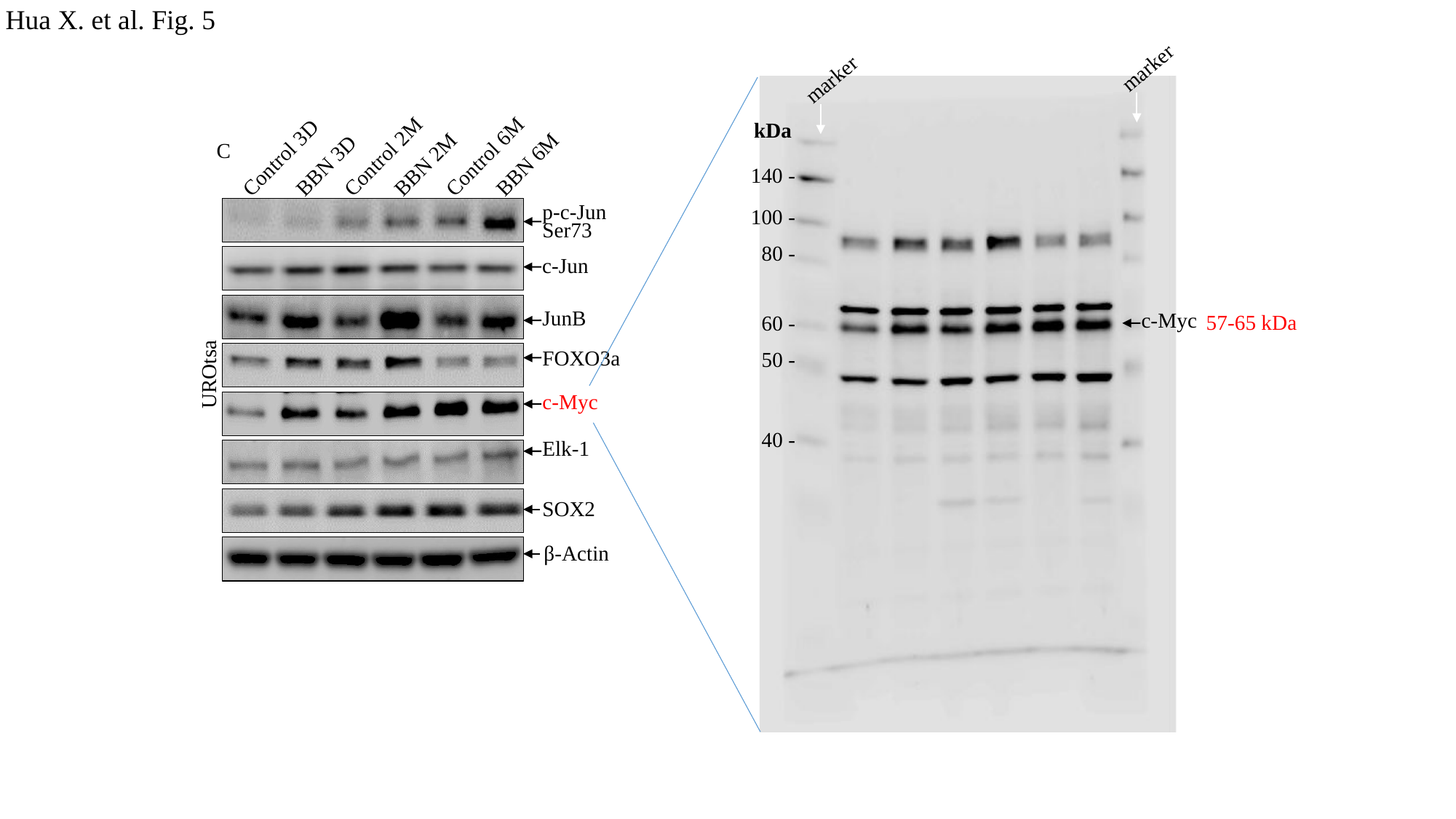

Hua X. et al. Fig. 5
marker
marker
kDa
Control 6M
C
Control 2M
Control 3D
BBN 3D
BBN 2M
BBN 6M
140 -
p-c-Jun Ser73
100 -
80 -
c-Jun
JunB
c-Myc
57-65 kDa
60 -
FOXO3a
50 -
UROtsa
c-Myc
40 -
Elk-1
SOX2
β-Actin

## Slide 29
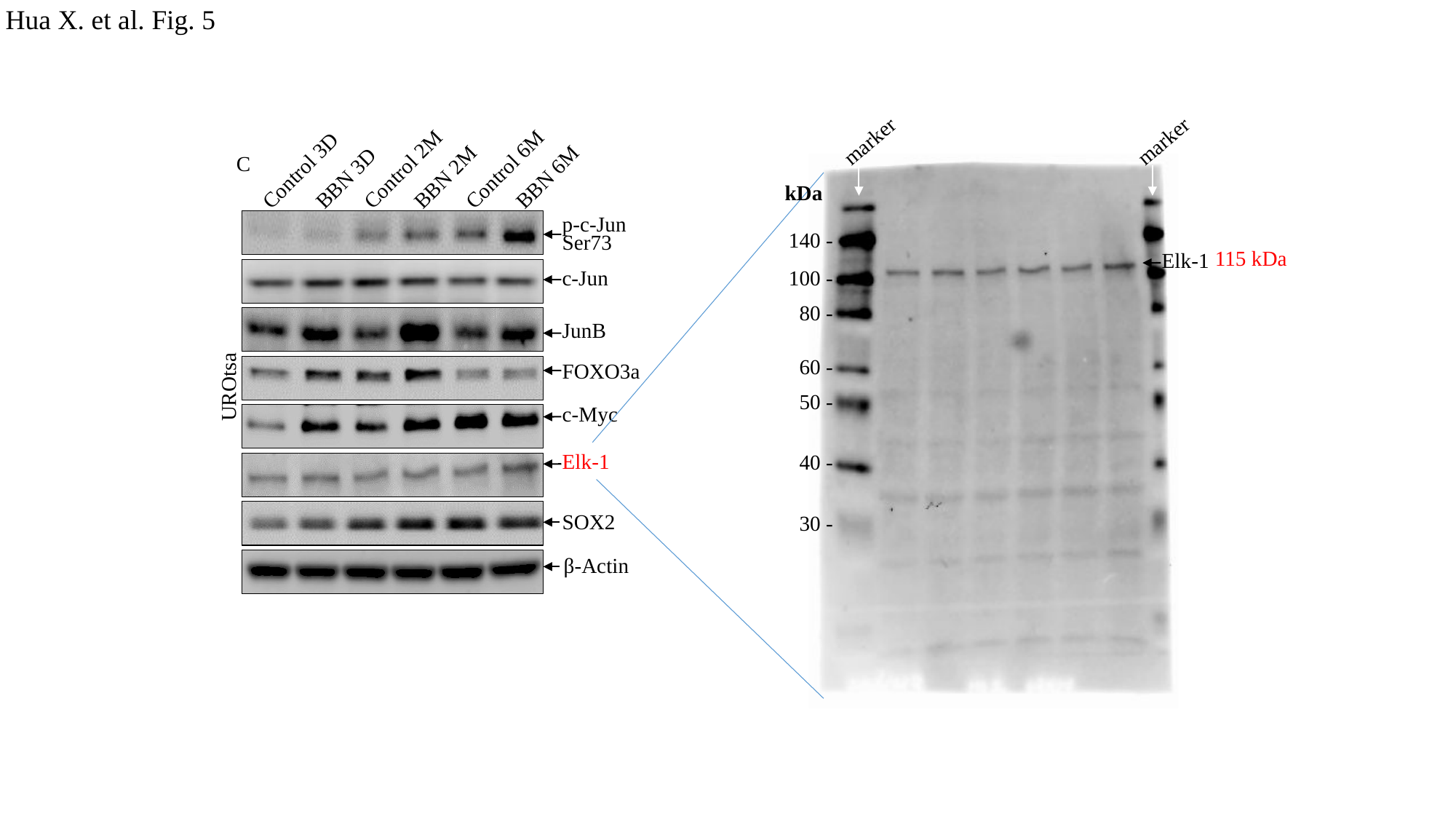

Hua X. et al. Fig. 5
marker
marker
Control 6M
C
Control 2M
Control 3D
BBN 3D
BBN 2M
BBN 6M
kDa
p-c-Jun Ser73
140 -
Elk-1
115 kDa
c-Jun
100 -
80 -
JunB
FOXO3a
60 -
UROtsa
50 -
c-Myc
Elk-1
40 -
SOX2
30 -
β-Actin

## Slide 30
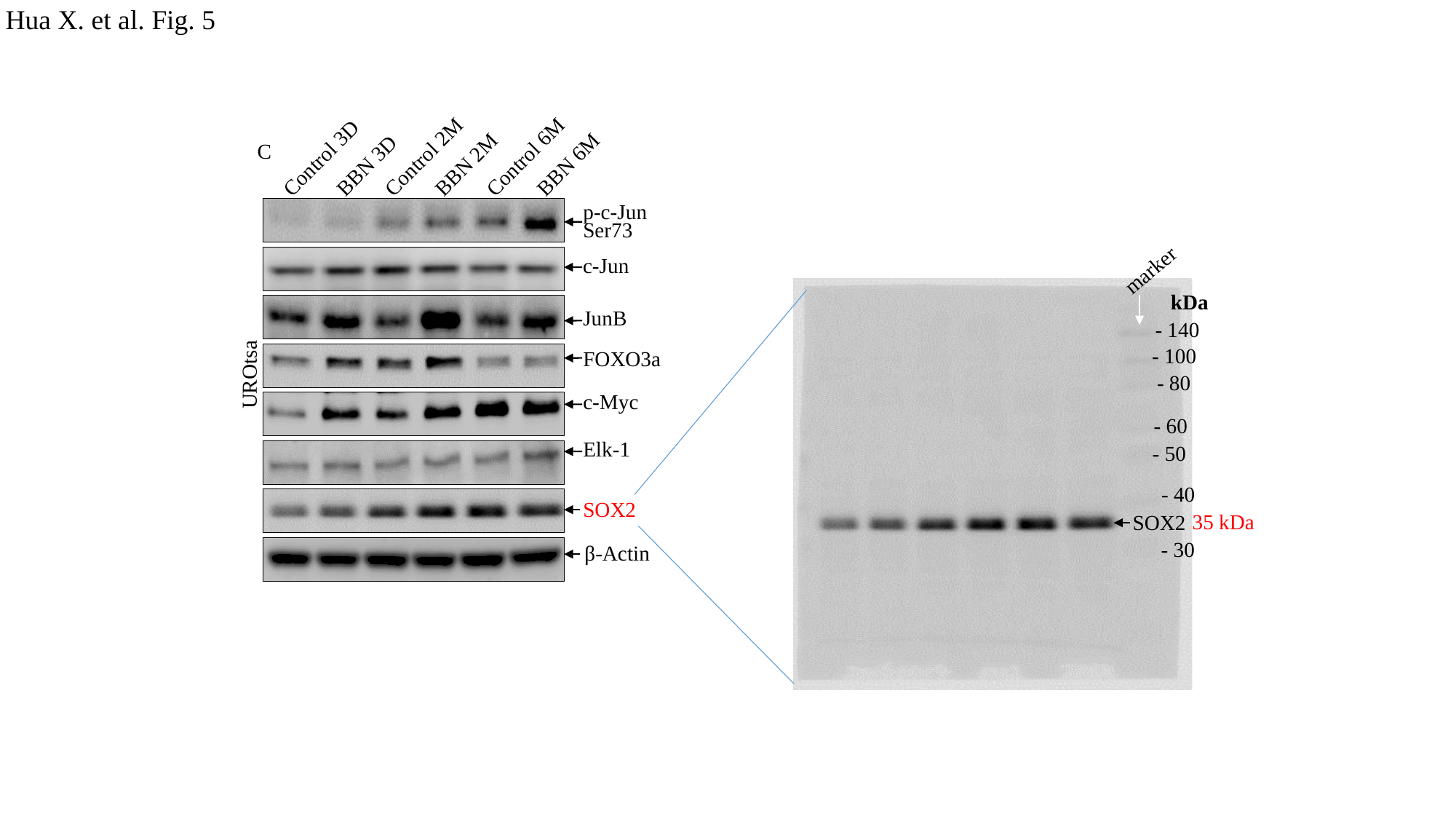

Hua X. et al. Fig. 5
Control 6M
C
Control 2M
Control 3D
BBN 3D
BBN 2M
BBN 6M
p-c-Jun Ser73
c-Jun
marker
kDa
JunB
- 140
FOXO3a
- 100
UROtsa
- 80
c-Myc
- 60
Elk-1
- 50
- 40
SOX2
SOX2
35 kDa
β-Actin
- 30

## Slide 31
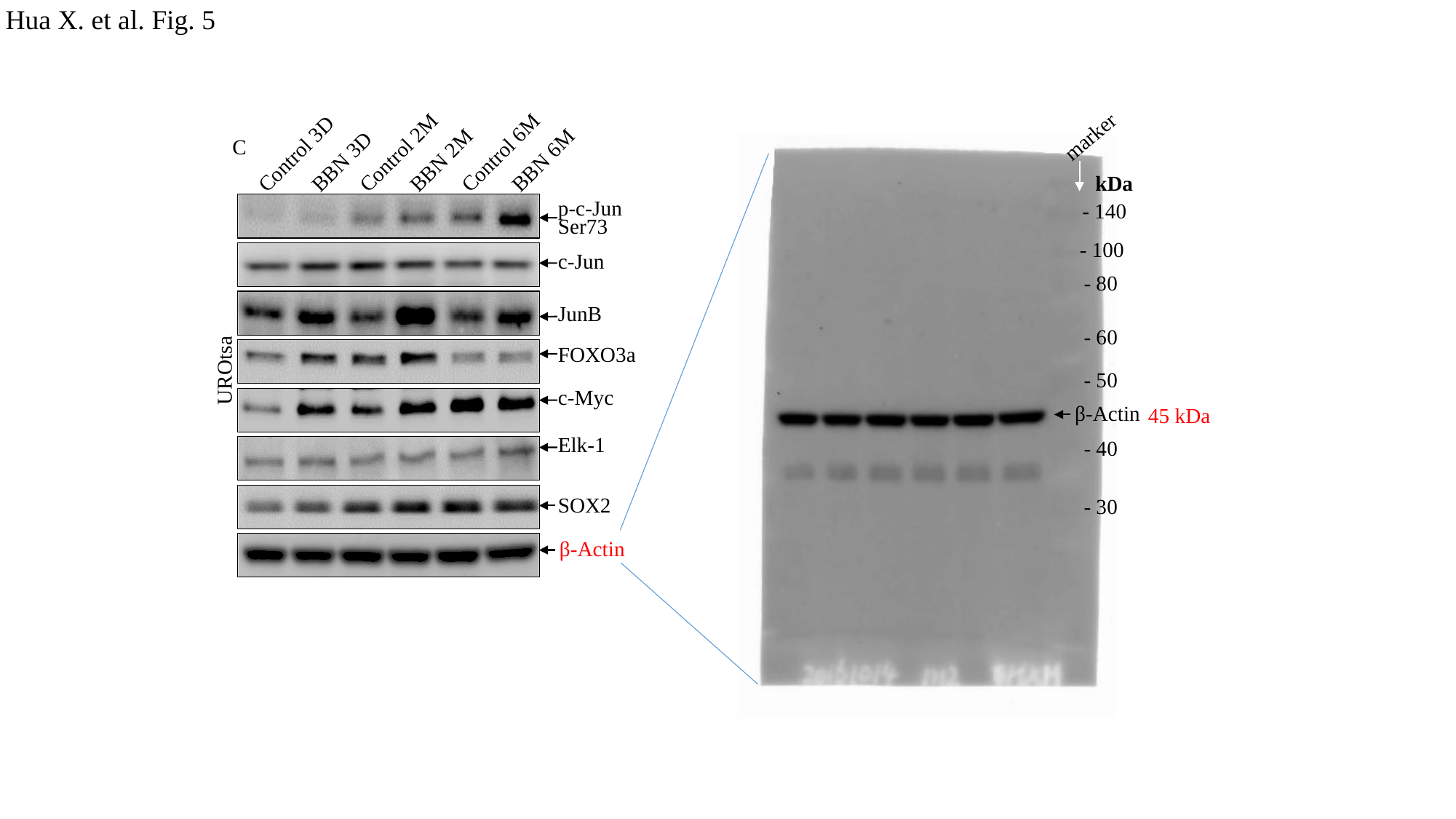

Hua X. et al. Fig. 5
marker
Control 6M
C
Control 2M
Control 3D
BBN 3D
BBN 2M
BBN 6M
kDa
p-c-Jun Ser73
- 140
- 100
c-Jun
- 80
JunB
- 60
FOXO3a
UROtsa
- 50
c-Myc
β-Actin
45 kDa
Elk-1
- 40
SOX2
- 30
β-Actin

## Slide 32
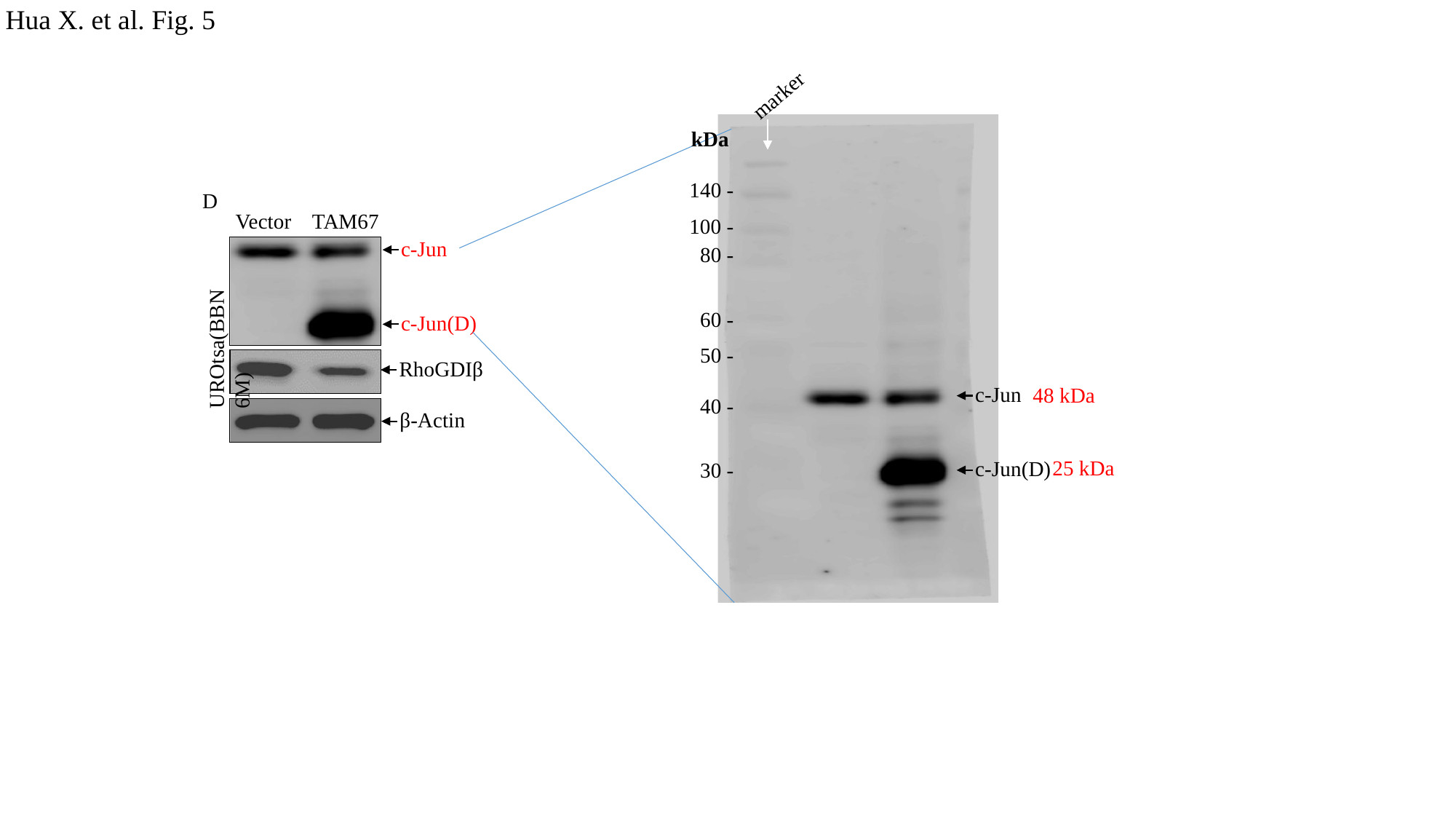

Hua X. et al. Fig. 5
marker
kDa
140 -
D
Vector TAM67
100 -
c-Jun
80 -
c-Jun(D)
60 -
UROtsa(BBN 6M)
50 -
RhoGDIβ
c-Jun
48 kDa
40 -
β-Actin
c-Jun(D)
25 kDa
30 -

## Slide 33
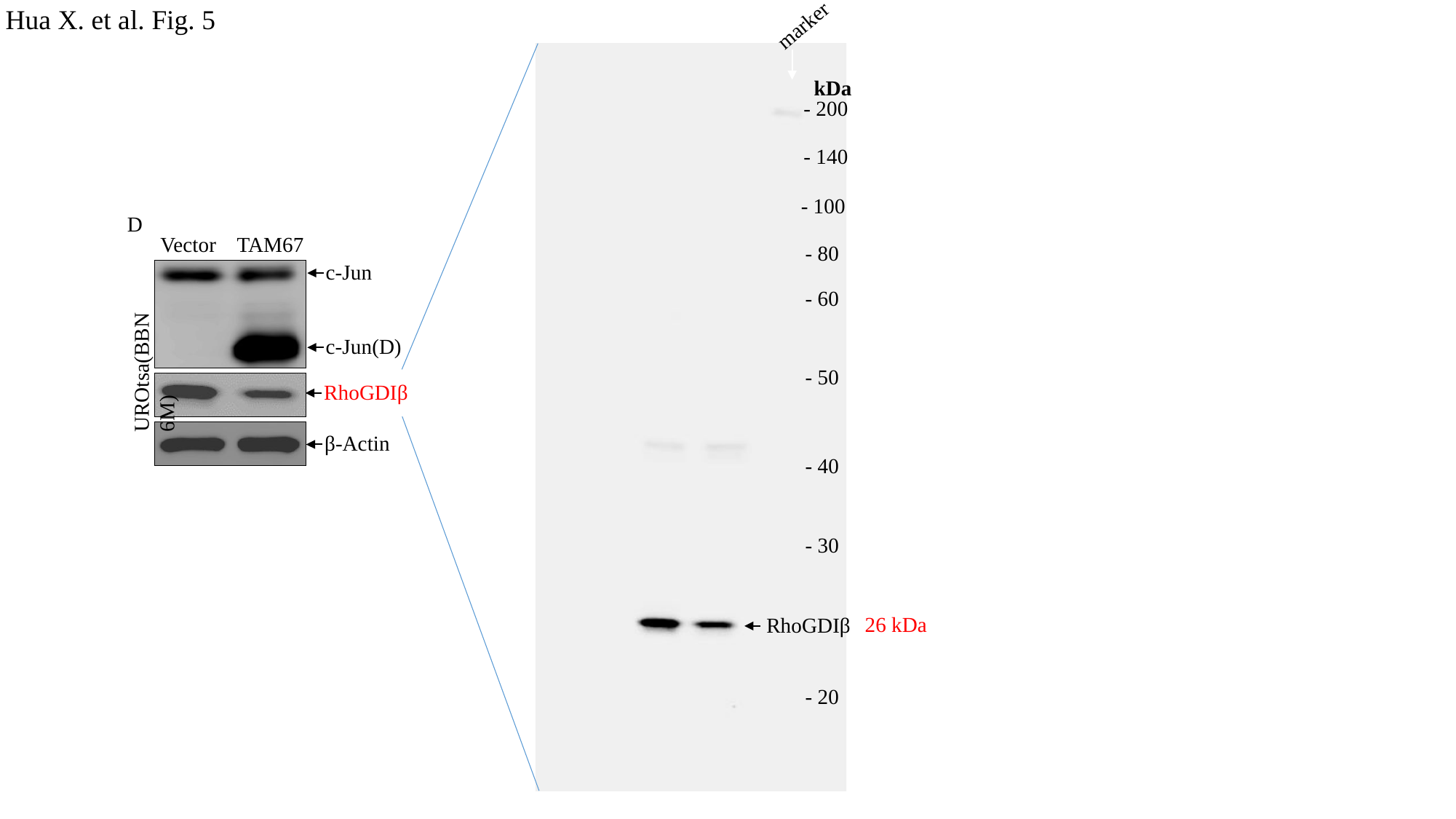

Hua X. et al. Fig. 5
marker
kDa
- 200
- 140
- 100
D
Vector TAM67
- 80
c-Jun
- 60
c-Jun(D)
UROtsa(BBN 6M)
- 50
RhoGDIβ
β-Actin
- 40
- 30
RhoGDIβ
26 kDa
- 20

## Slide 34
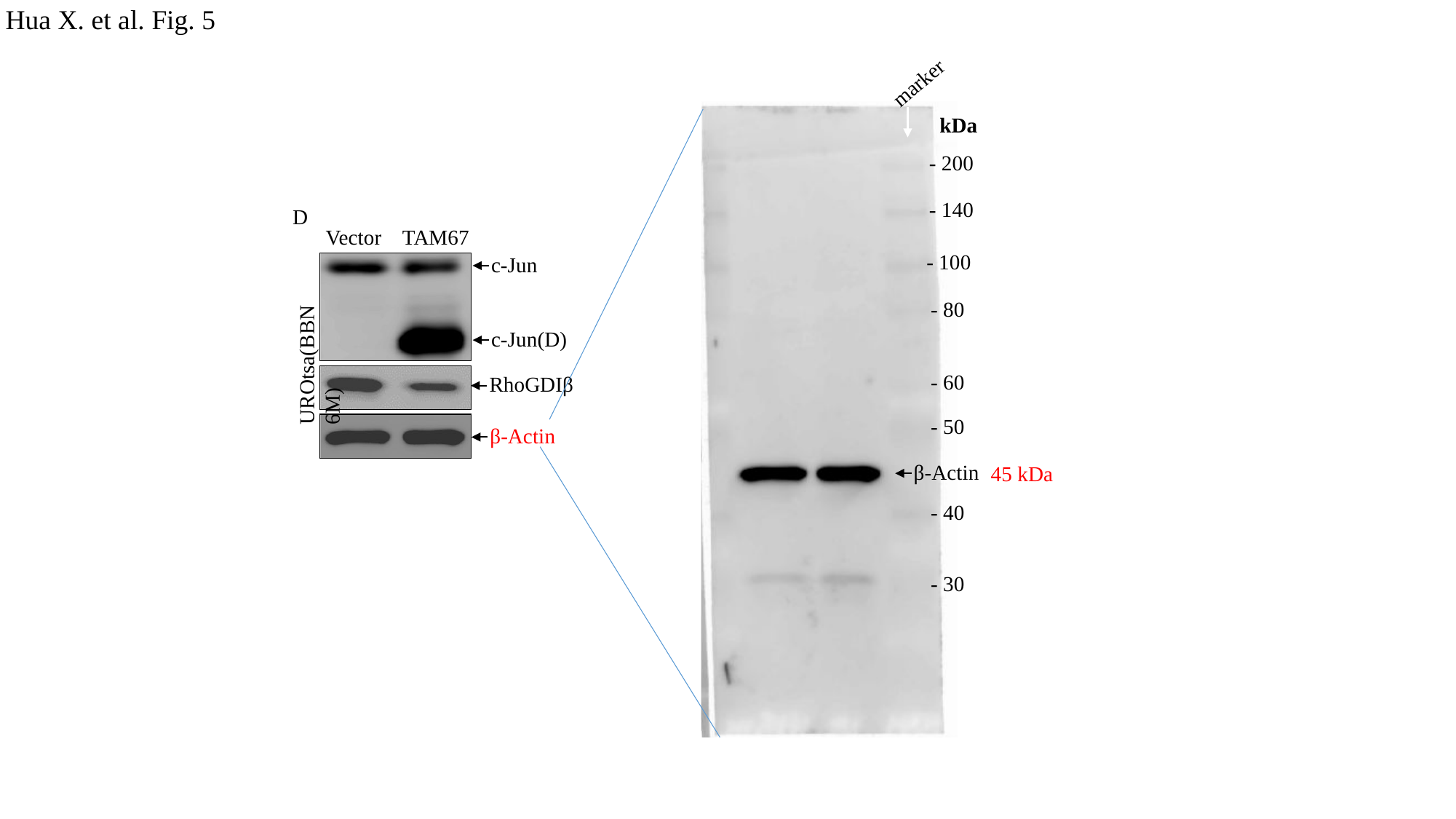

Hua X. et al. Fig. 5
marker
kDa
- 200
- 140
D
Vector TAM67
c-Jun
- 100
- 80
c-Jun(D)
UROtsa(BBN 6M)
RhoGDIβ
- 60
- 50
β-Actin
β-Actin
45 kDa
- 40
- 30

## Slide 35
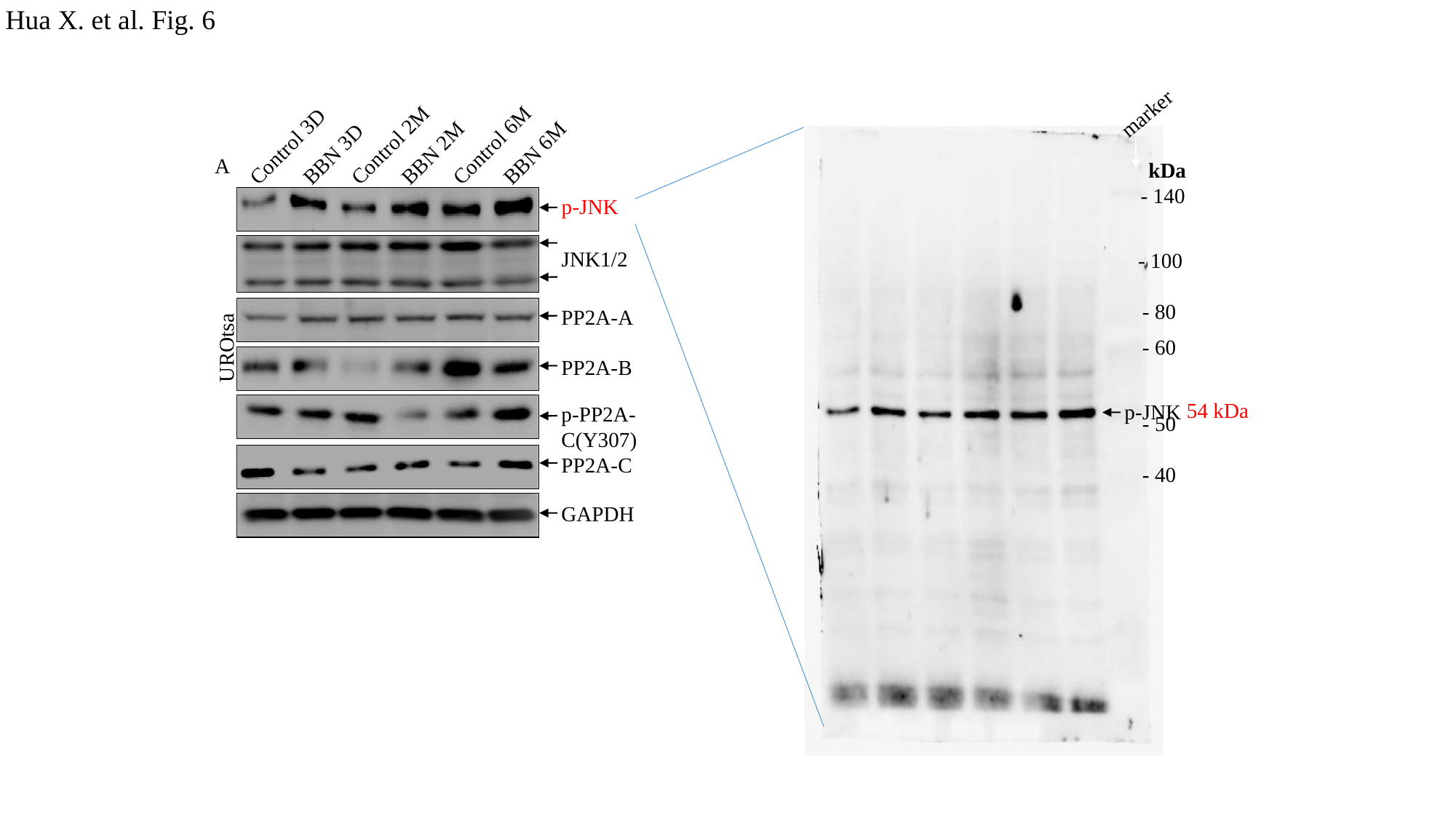

Hua X. et al. Fig. 6
marker
Control 6M
Control 2M
Control 3D
BBN 3D
BBN 2M
BBN 6M
A
kDa
- 140
p-JNK
JNK1/2
- 100
PP2A-A
- 80
UROtsa
- 60
PP2A-B
p-JNK
p-PP2A-C(Y307)
54 kDa
- 50
PP2A-C
- 40
GAPDH

## Slide 36
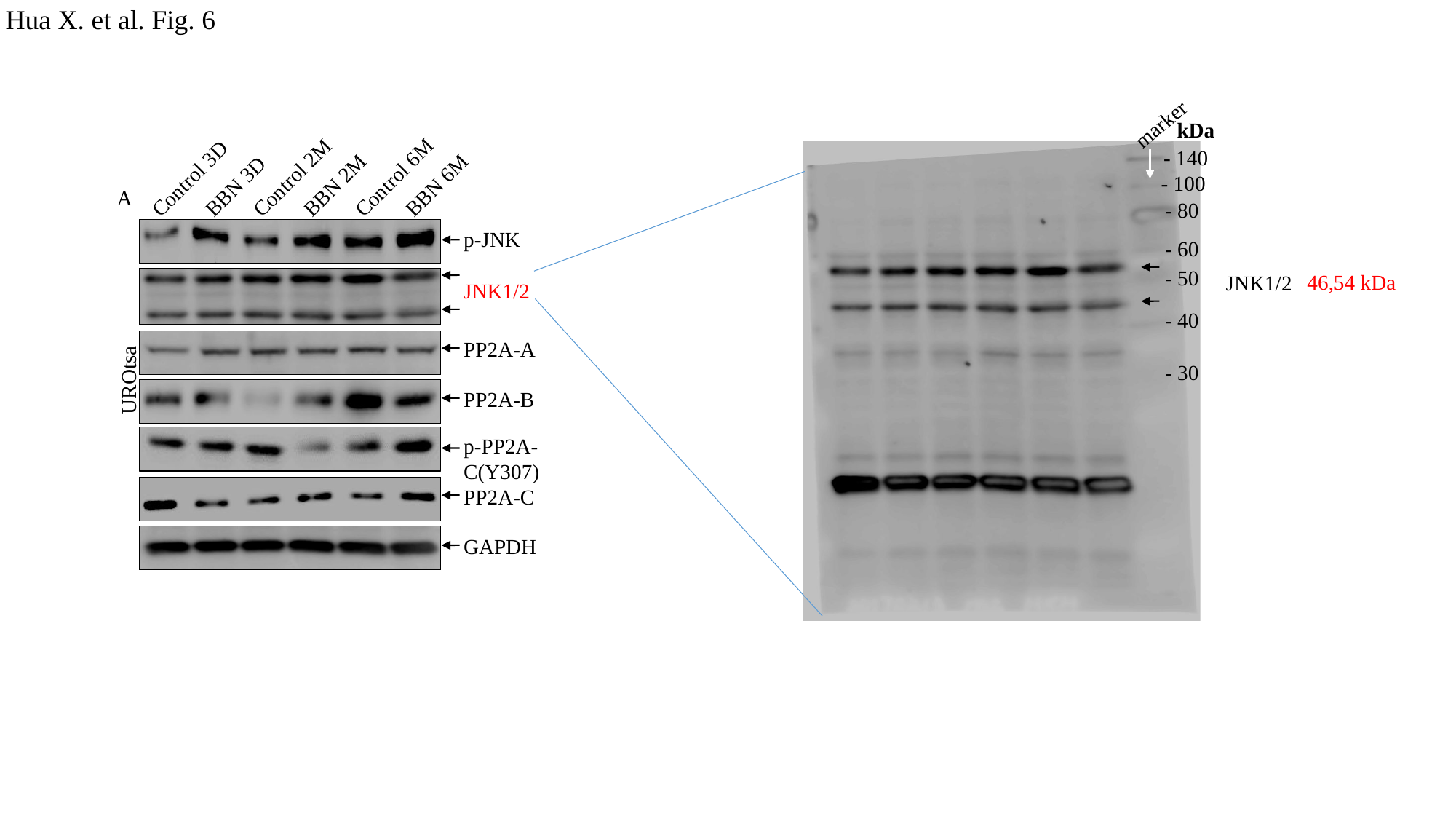

Hua X. et al. Fig. 6
marker
kDa
- 140
Control 6M
Control 2M
Control 3D
BBN 3D
BBN 2M
BBN 6M
- 100
A
- 80
p-JNK
- 60
JNK1/2
- 50
46,54 kDa
JNK1/2
- 40
PP2A-A
UROtsa
- 30
PP2A-B
p-PP2A-C(Y307)
PP2A-C
GAPDH

## Slide 37
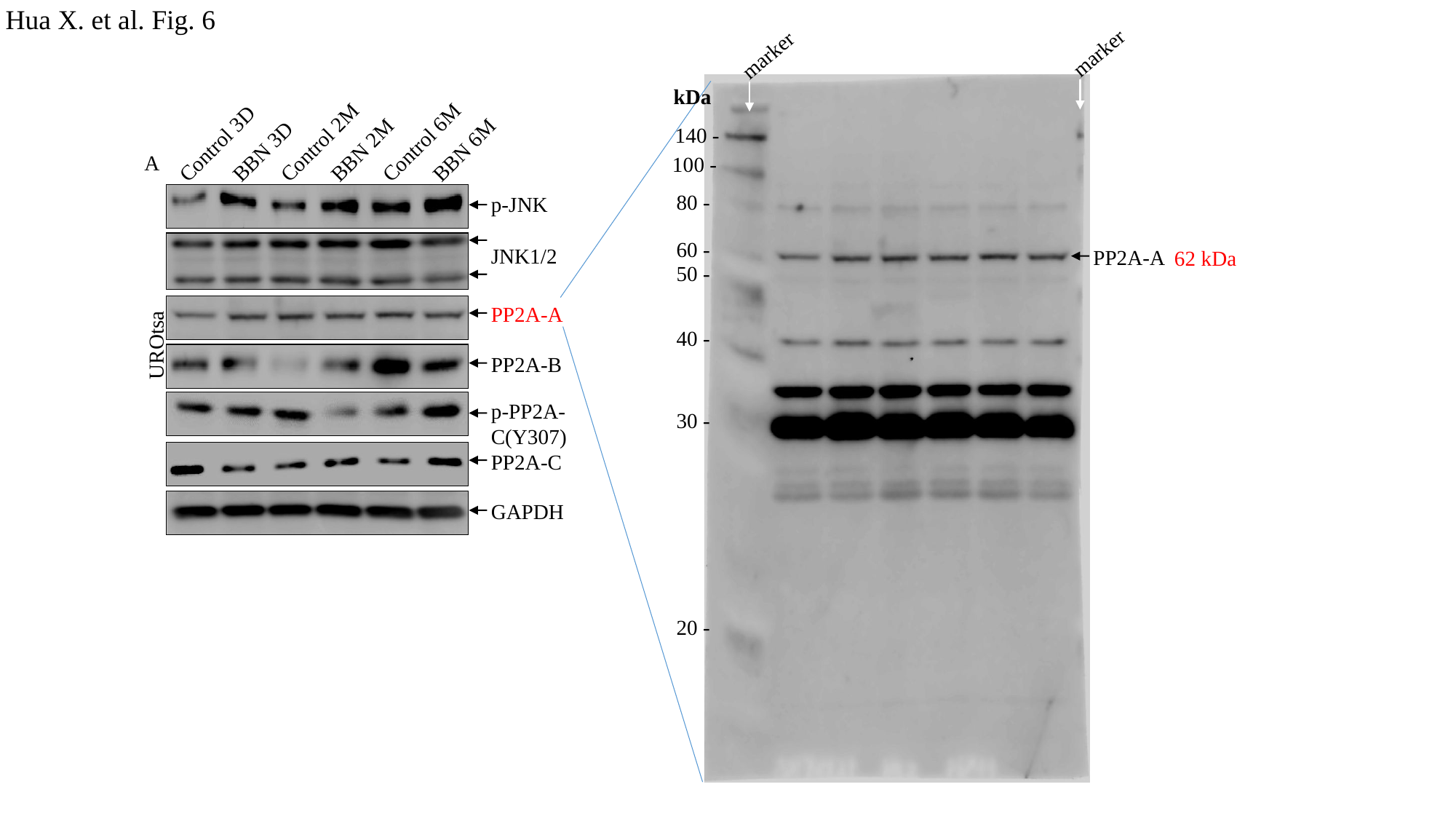

Hua X. et al. Fig. 6
marker
marker
kDa
Control 6M
Control 2M
140 -
Control 3D
BBN 3D
BBN 2M
BBN 6M
A
100 -
p-JNK
80 -
JNK1/2
60 -
PP2A-A
62 kDa
50 -
PP2A-A
UROtsa
40 -
PP2A-B
p-PP2A-C(Y307)
30 -
PP2A-C
GAPDH
20 -

## Slide 38
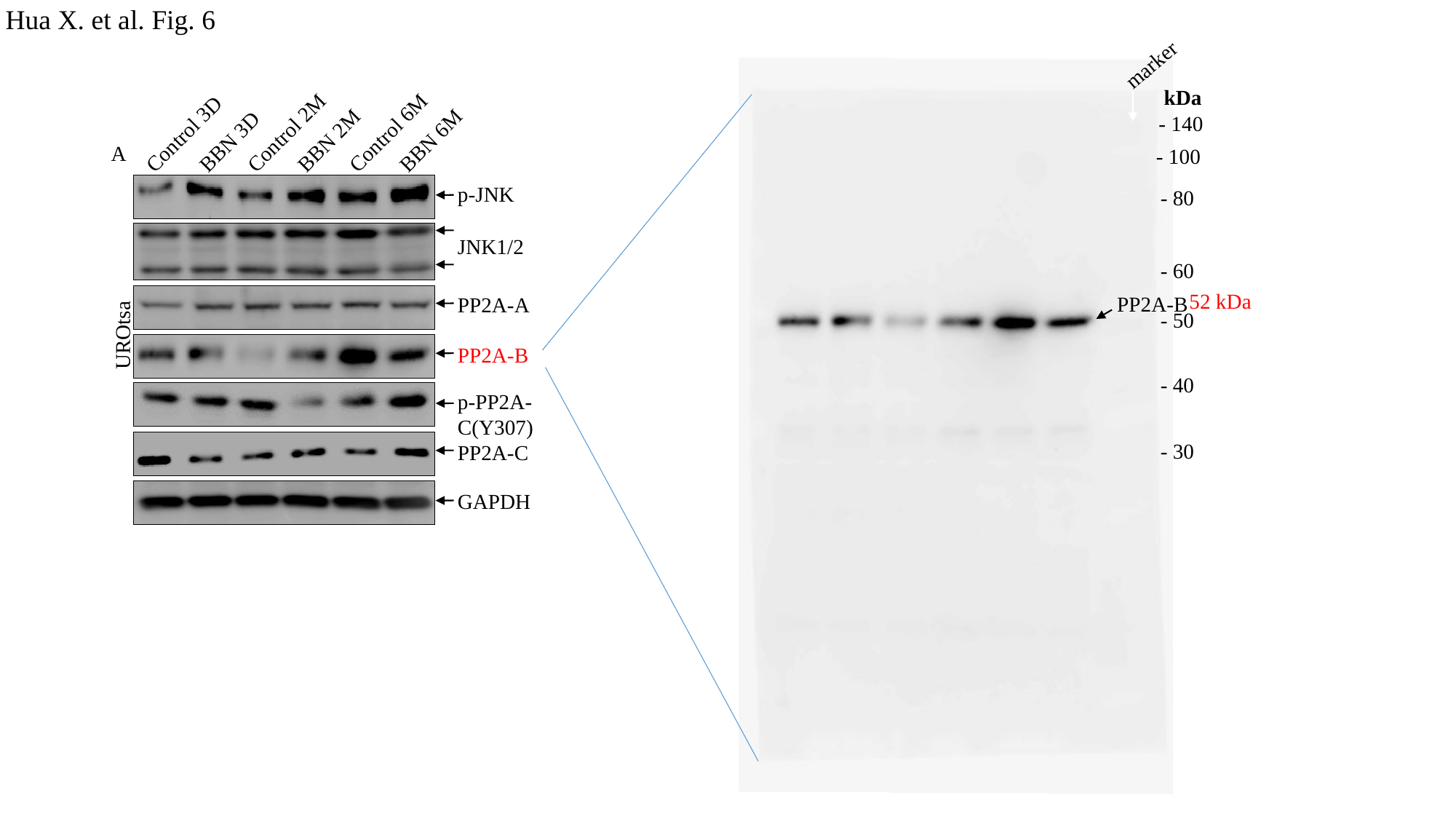

Hua X. et al. Fig. 6
marker
kDa
Control 6M
Control 2M
- 140
Control 3D
BBN 3D
BBN 2M
BBN 6M
A
- 100
p-JNK
- 80
JNK1/2
- 60
PP2A-B
PP2A-A
52 kDa
- 50
UROtsa
PP2A-B
- 40
p-PP2A-C(Y307)
PP2A-C
- 30
GAPDH

## Slide 39
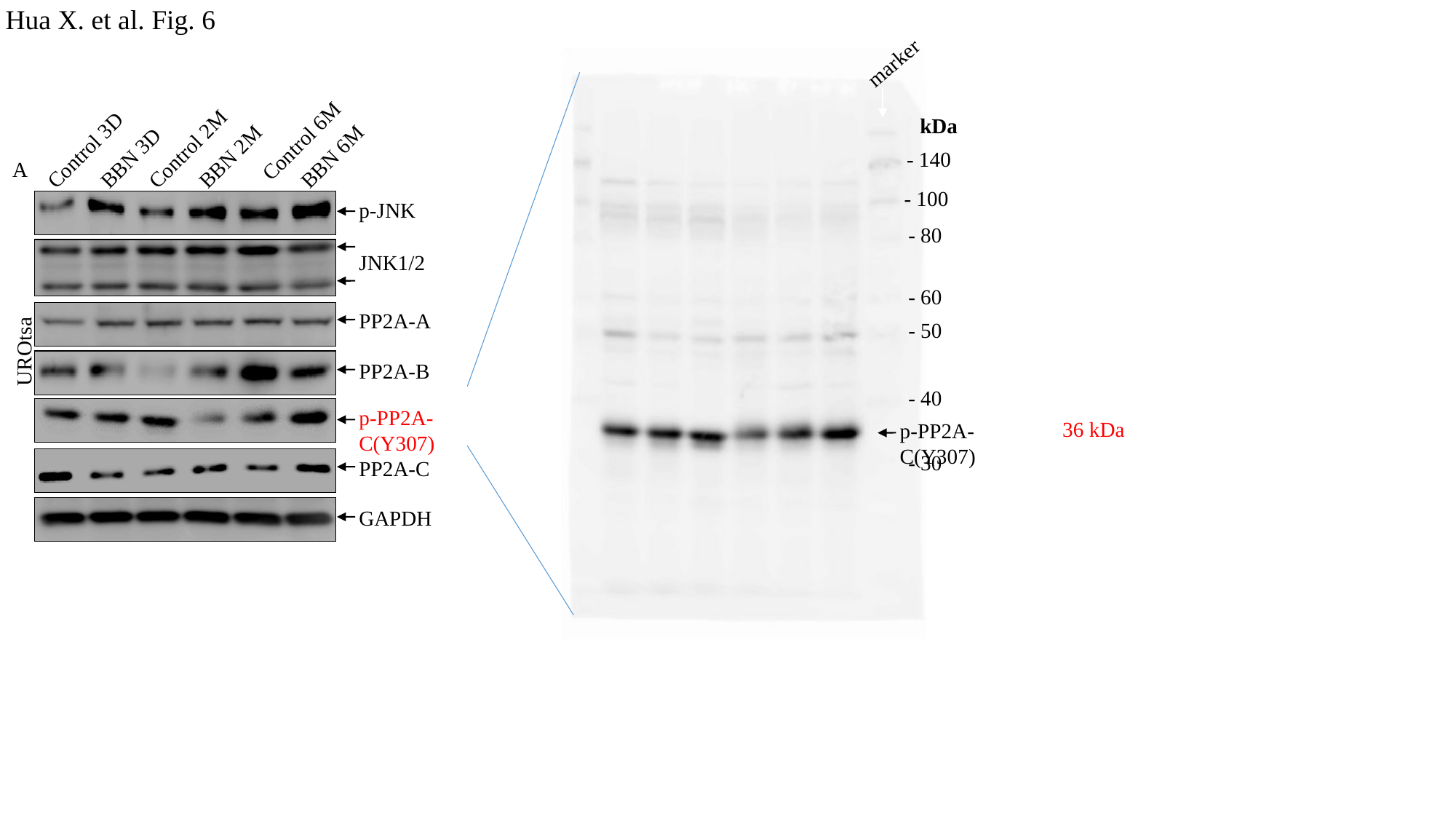

Hua X. et al. Fig. 6
marker
kDa
Control 6M
Control 2M
Control 3D
BBN 3D
BBN 2M
BBN 6M
- 140
A
- 100
p-JNK
- 80
JNK1/2
- 60
PP2A-A
- 50
UROtsa
PP2A-B
- 40
p-PP2A-C(Y307)
p-PP2A-C(Y307)
36 kDa
PP2A-C
- 30
GAPDH

## Slide 40
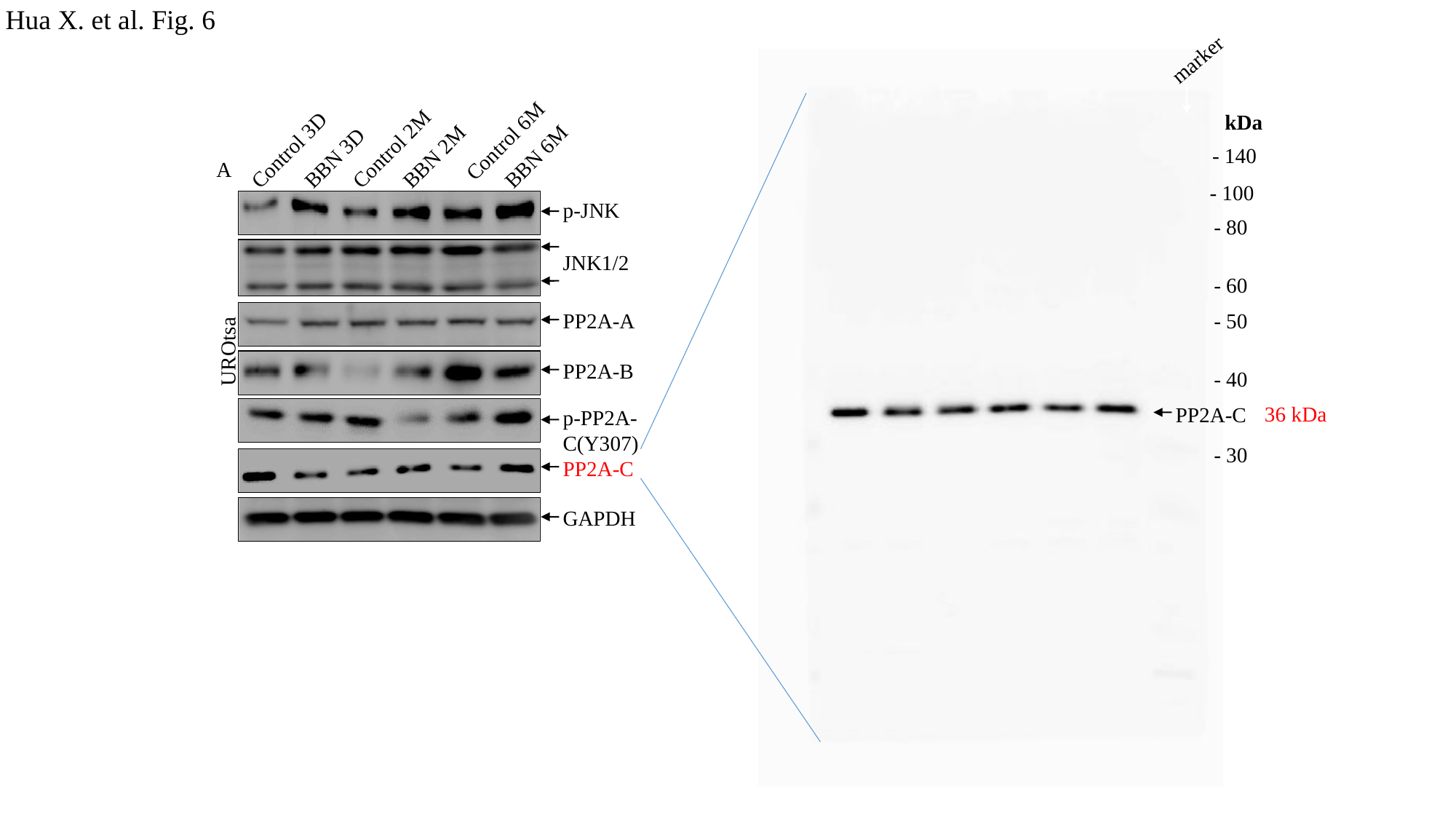

Hua X. et al. Fig. 6
marker
kDa
Control 6M
Control 2M
Control 3D
BBN 3D
BBN 2M
BBN 6M
- 140
A
- 100
p-JNK
- 80
JNK1/2
- 60
PP2A-A
- 50
UROtsa
PP2A-B
- 40
PP2A-C
p-PP2A-C(Y307)
36 kDa
- 30
PP2A-C
GAPDH

## Slide 41
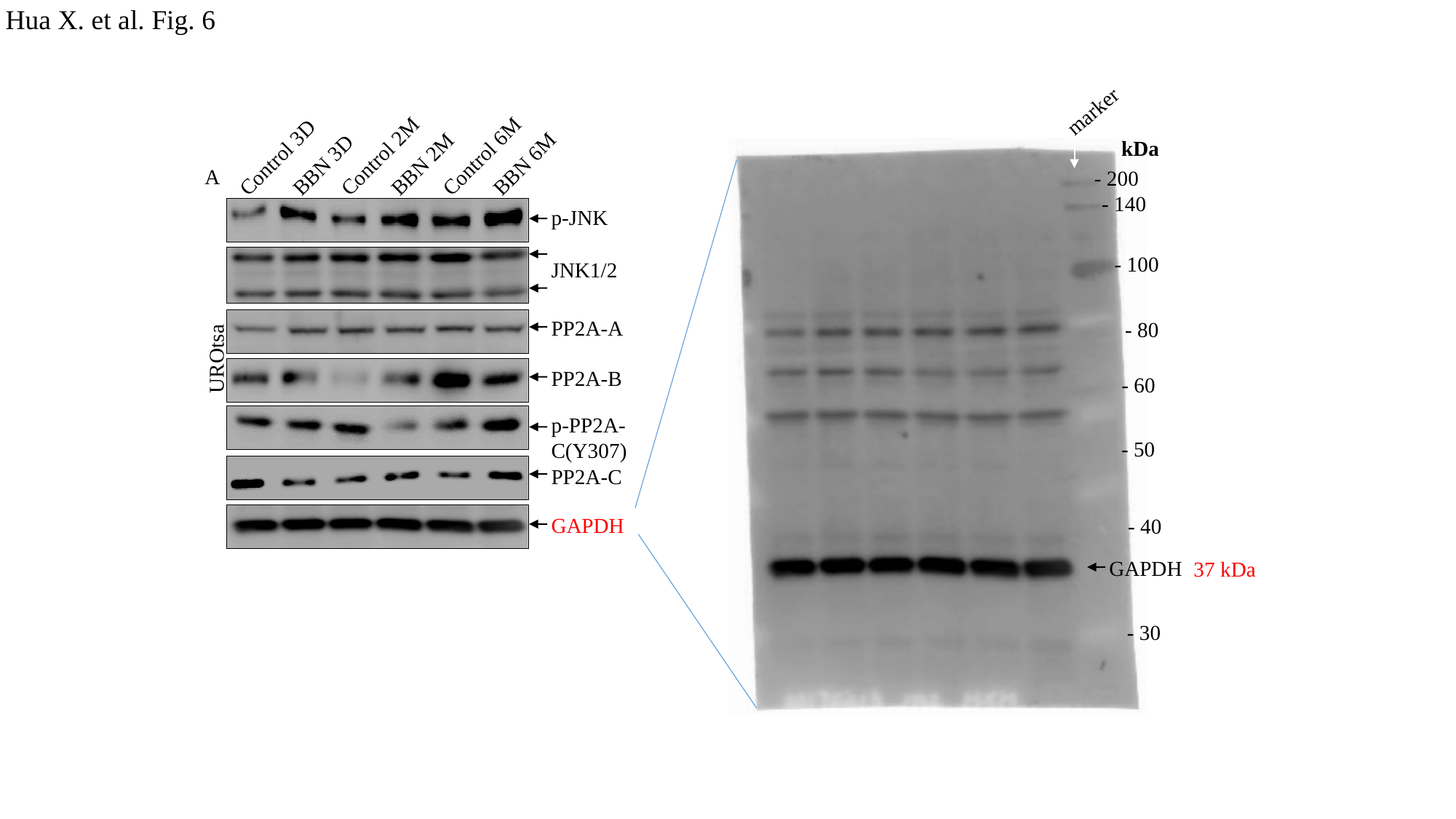

Hua X. et al. Fig. 6
marker
Control 6M
Control 2M
kDa
Control 3D
BBN 3D
BBN 2M
BBN 6M
A
- 200
- 140
p-JNK
JNK1/2
- 100
PP2A-A
- 80
UROtsa
PP2A-B
- 60
p-PP2A-C(Y307)
- 50
PP2A-C
GAPDH
- 40
GAPDH
37 kDa
- 30

## Slide 42
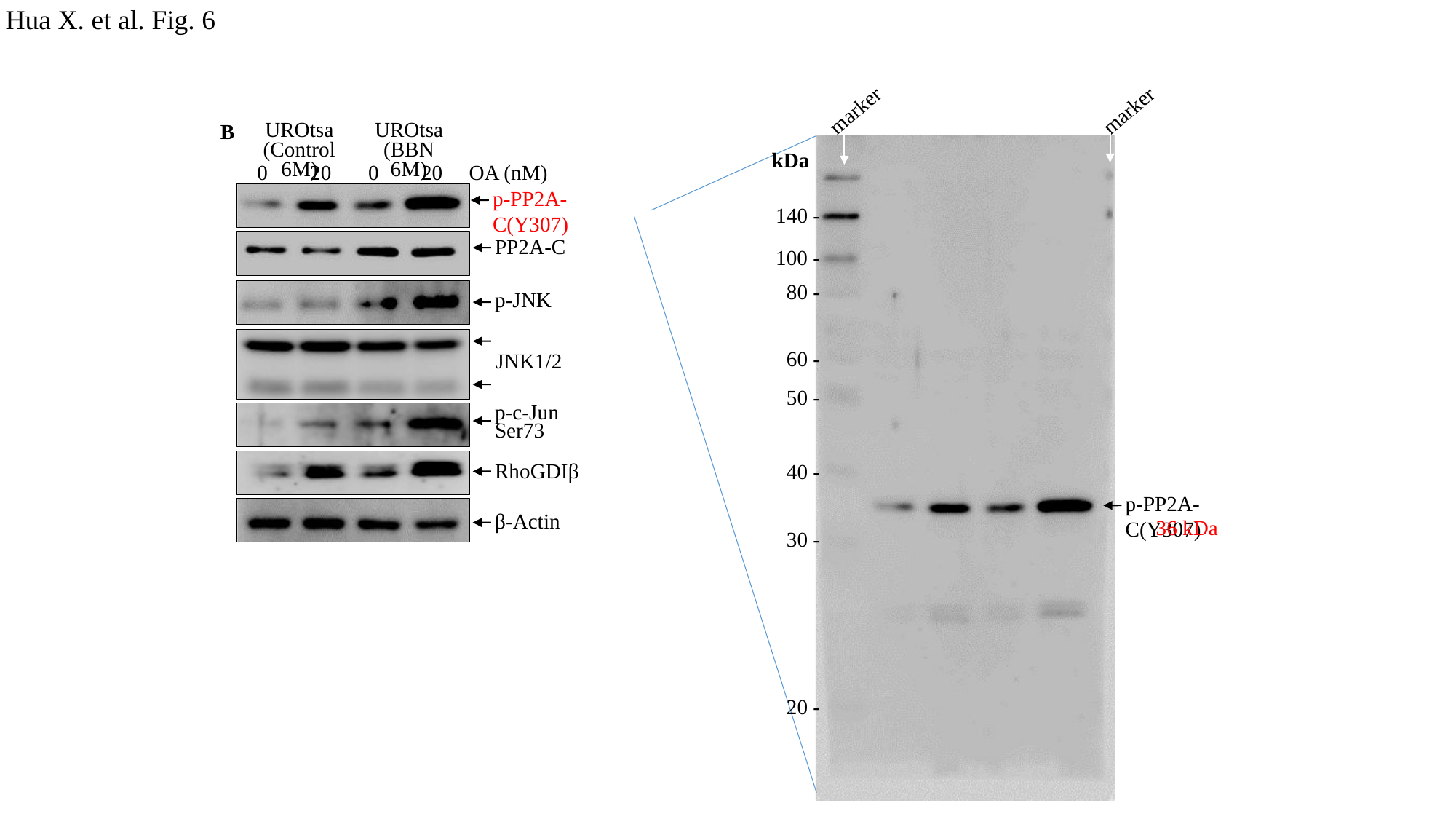

Hua X. et al. Fig. 6
marker
marker
B
UROtsa
(Control 6M)
UROtsa
(BBN 6M)
kDa
0 20 0 20 OA (nM)
p-PP2A-C(Y307)
140 -
PP2A-C
100 -
80 -
p-JNK
JNK1/2
60 -
50 -
p-c-Jun Ser73
RhoGDIβ
40 -
p-PP2A-C(Y307)
β-Actin
36 kDa
30 -
20 -

## Slide 43
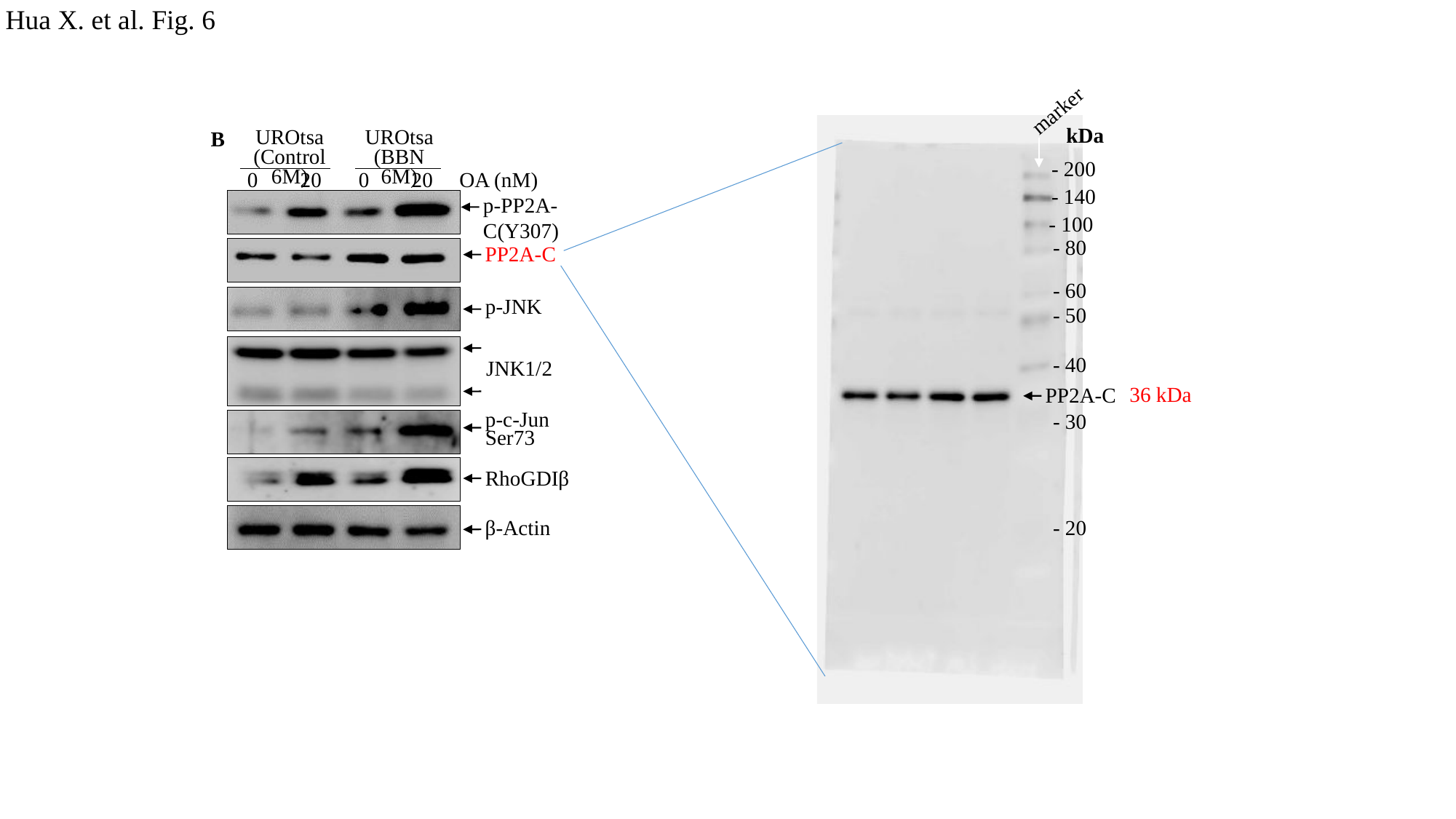

Hua X. et al. Fig. 6
marker
B
UROtsa
(Control 6M)
UROtsa
(BBN 6M)
kDa
- 200
0 20 0 20 OA (nM)
- 140
p-PP2A-C(Y307)
- 100
PP2A-C
- 80
- 60
p-JNK
- 50
JNK1/2
- 40
PP2A-C
36 kDa
p-c-Jun Ser73
- 30
RhoGDIβ
β-Actin
- 20

## Slide 44
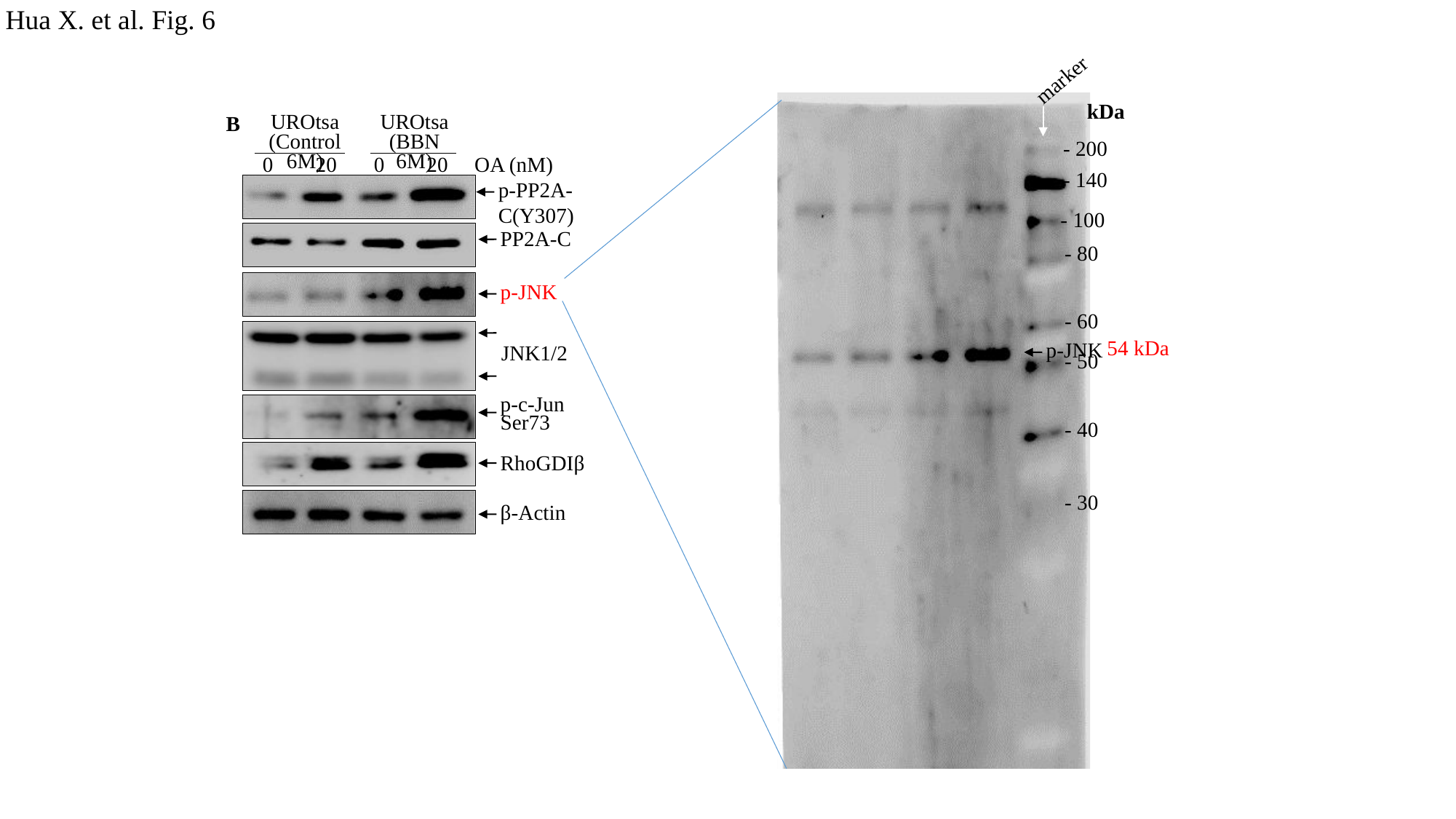

Hua X. et al. Fig. 6
marker
kDa
B
UROtsa
(Control 6M)
UROtsa
(BBN 6M)
- 200
0 20 0 20 OA (nM)
- 140
p-PP2A-C(Y307)
- 100
PP2A-C
- 80
p-JNK
- 60
p-JNK
JNK1/2
54 kDa
- 50
p-c-Jun Ser73
- 40
RhoGDIβ
- 30
β-Actin

## Slide 45
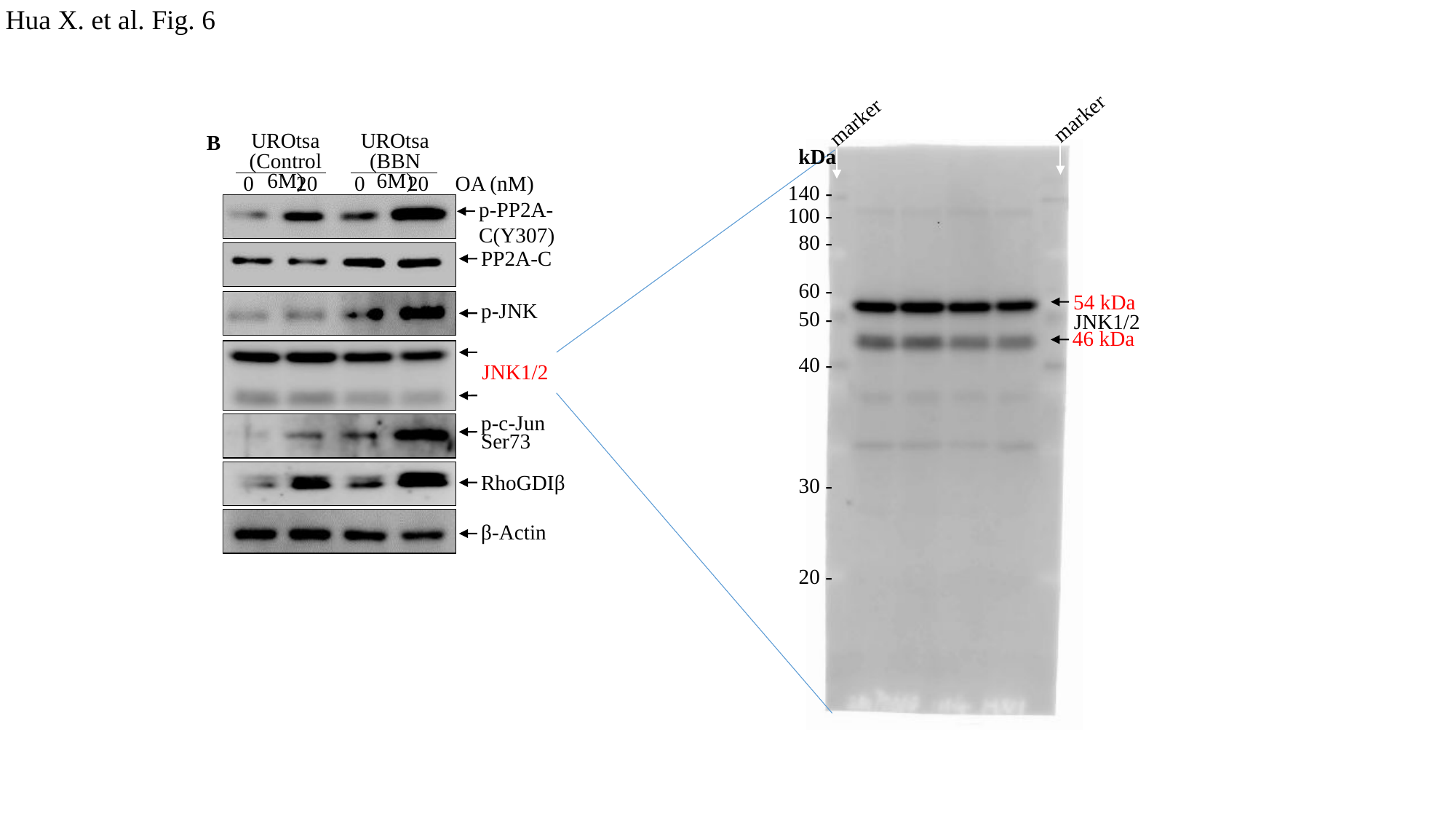

Hua X. et al. Fig. 6
marker
marker
B
UROtsa
(Control 6M)
UROtsa
(BBN 6M)
kDa
0 20 0 20 OA (nM)
140 -
p-PP2A-C(Y307)
100 -
80 -
PP2A-C
60 -
54 kDa
p-JNK
JNK1/2
50 -
46 kDa
JNK1/2
40 -
p-c-Jun Ser73
RhoGDIβ
30 -
β-Actin
20 -

## Slide 46
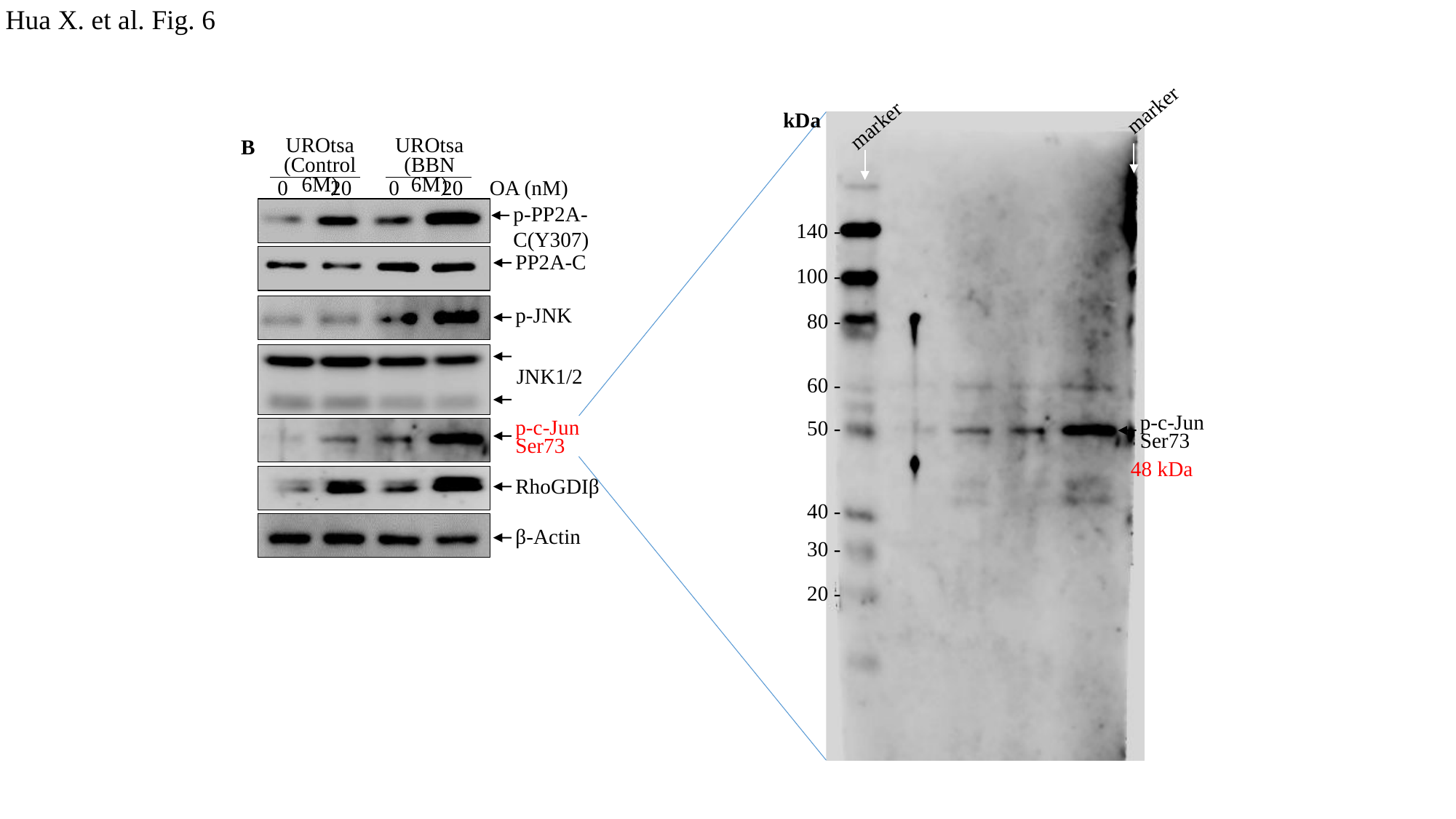

Hua X. et al. Fig. 6
marker
marker
kDa
B
UROtsa
(Control 6M)
UROtsa
(BBN 6M)
0 20 0 20 OA (nM)
p-PP2A-C(Y307)
140 -
PP2A-C
100 -
p-JNK
80 -
JNK1/2
60 -
p-c-Jun Ser73
p-c-Jun Ser73
50 -
48 kDa
RhoGDIβ
40 -
β-Actin
30 -
20 -

## Slide 47
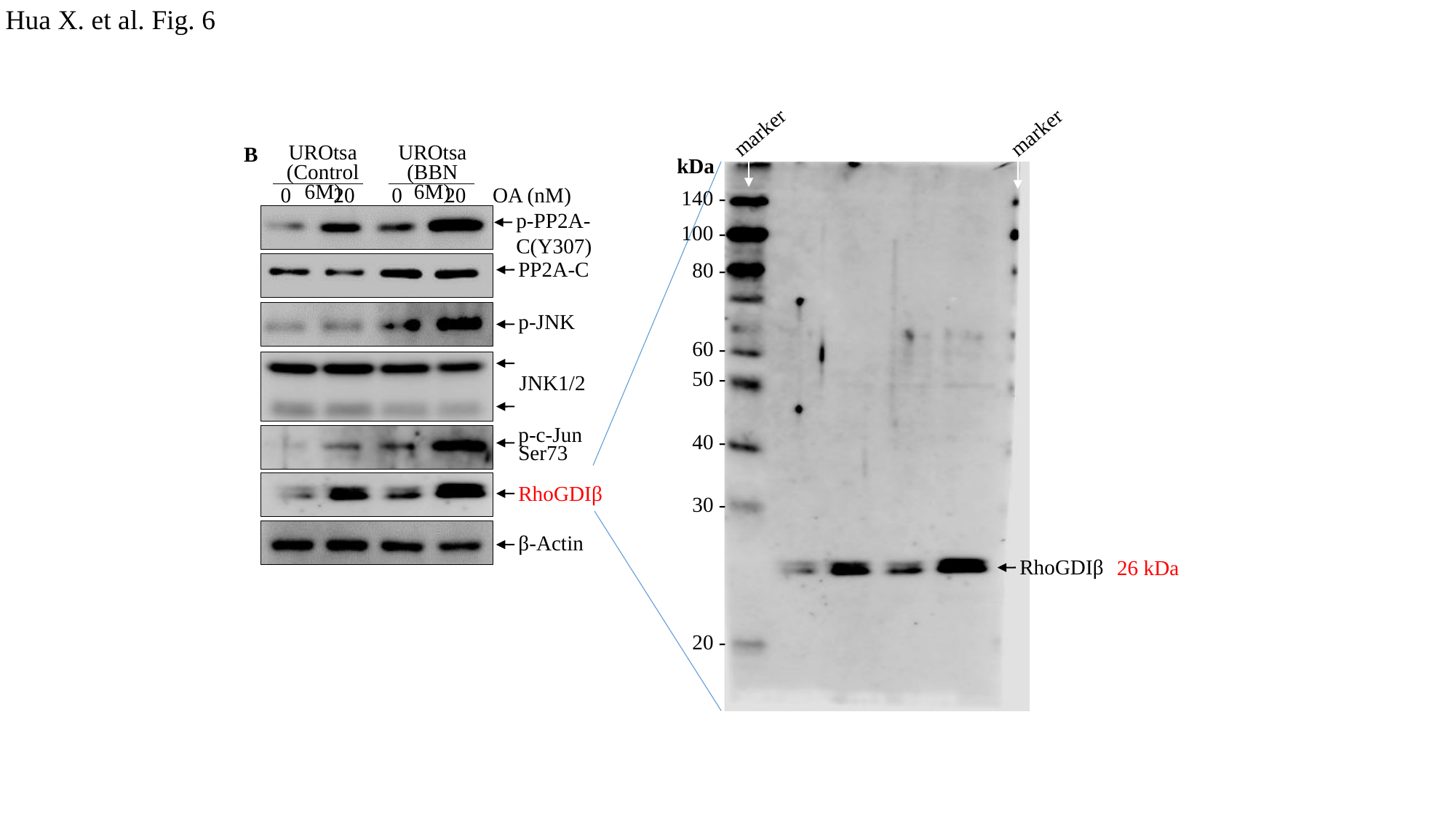

Hua X. et al. Fig. 6
marker
marker
B
UROtsa
(Control 6M)
UROtsa
(BBN 6M)
kDa
0 20 0 20 OA (nM)
140 -
p-PP2A-C(Y307)
100 -
PP2A-C
80 -
p-JNK
60 -
JNK1/2
50 -
p-c-Jun Ser73
40 -
RhoGDIβ
30 -
β-Actin
RhoGDIβ
26 kDa
20 -

## Slide 48
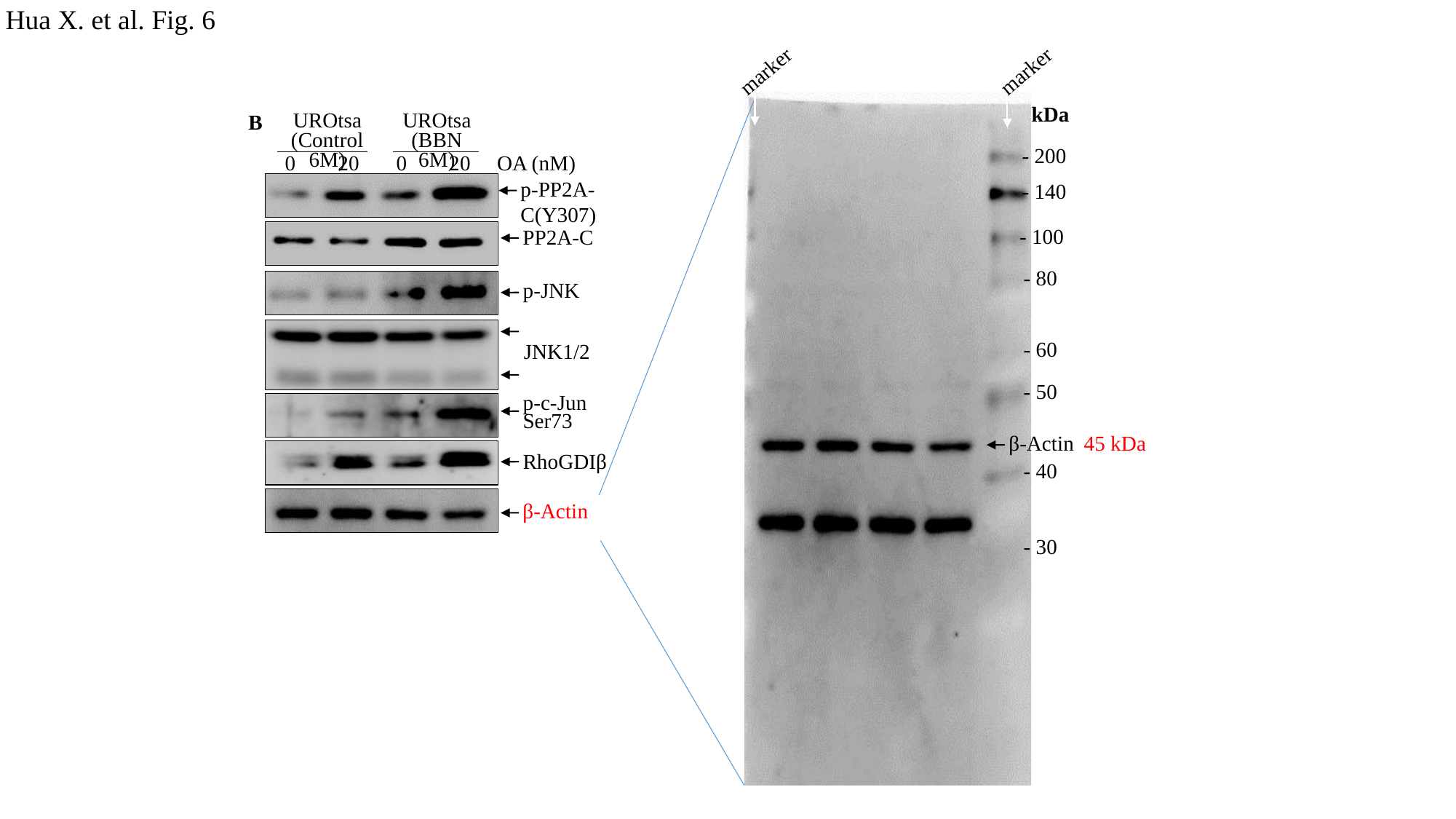

Hua X. et al. Fig. 6
marker
marker
kDa
B
UROtsa
(Control 6M)
UROtsa
(BBN 6M)
0 20 0 20 OA (nM)
- 200
p-PP2A-C(Y307)
- 140
PP2A-C
- 100
- 80
p-JNK
JNK1/2
- 60
- 50
p-c-Jun Ser73
β-Actin
45 kDa
RhoGDIβ
- 40
β-Actin
- 30

## Slide 49
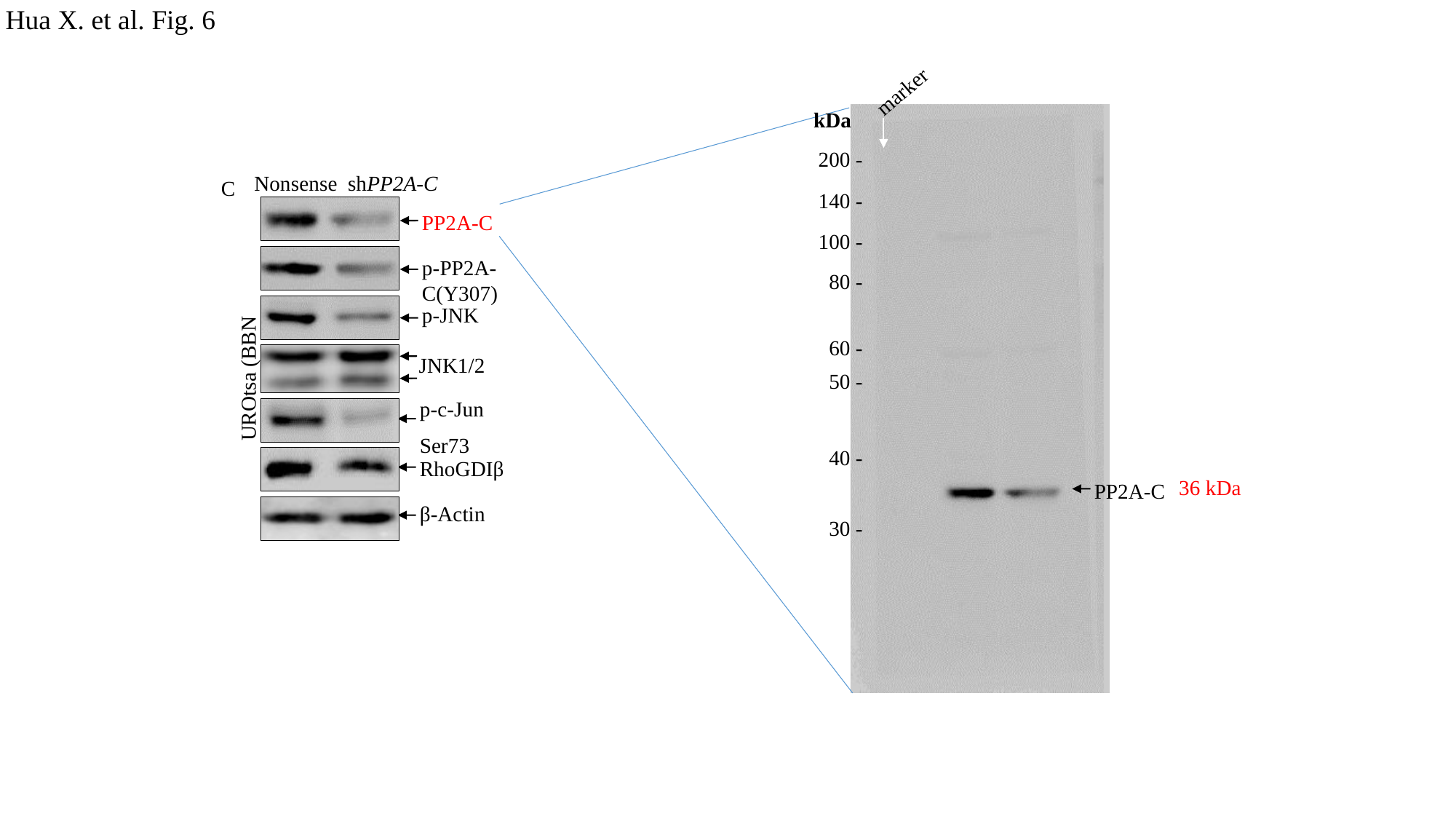

Hua X. et al. Fig. 6
marker
kDa
200 -
Nonsense shPP2A-C
C
140 -
PP2A-C
100 -
p-PP2A-C(Y307)
80 -
p-JNK
60 -
UROtsa (BBN 6M)
JNK1/2
50 -
p-c-Jun Ser73
40 -
RhoGDIβ
PP2A-C
36 kDa
β-Actin
30 -

## Slide 50
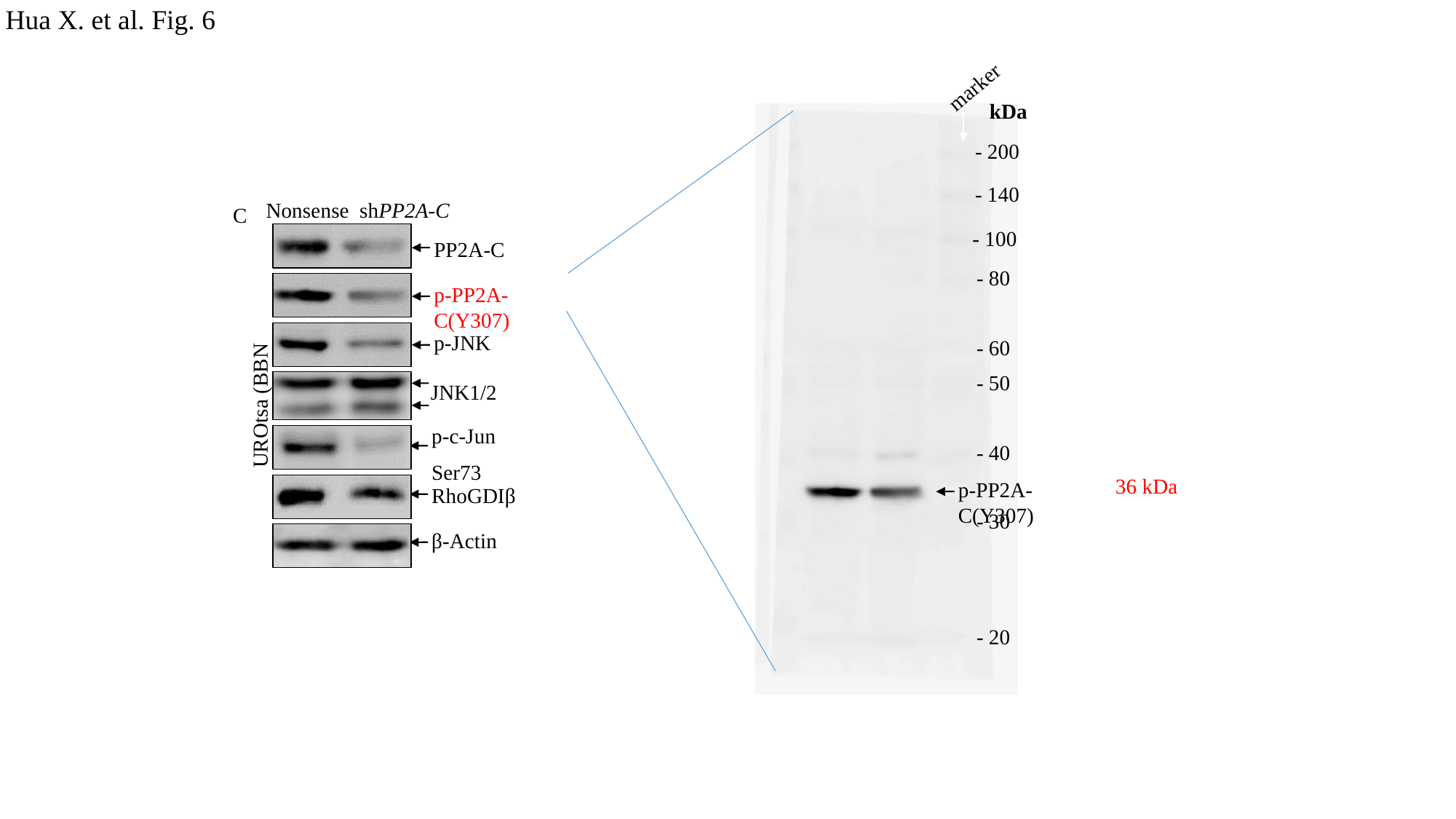

Hua X. et al. Fig. 6
marker
kDa
- 200
- 140
Nonsense shPP2A-C
C
- 100
PP2A-C
- 80
p-PP2A-C(Y307)
p-JNK
- 60
UROtsa (BBN 6M)
- 50
JNK1/2
p-c-Jun Ser73
- 40
p-PP2A-C(Y307)
36 kDa
RhoGDIβ
- 30
β-Actin
- 20

## Slide 51
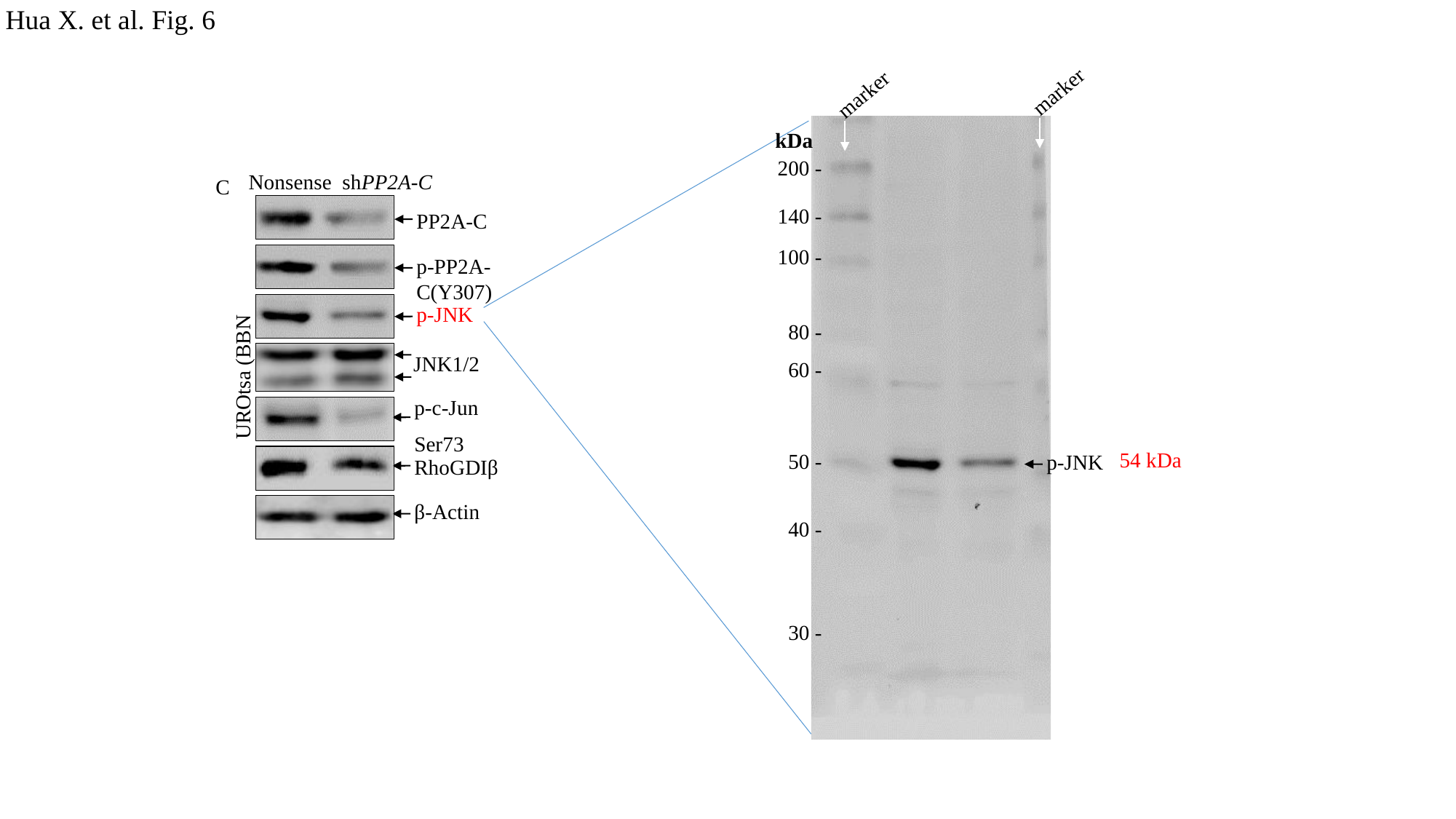

Hua X. et al. Fig. 6
marker
marker
kDa
200 -
Nonsense shPP2A-C
C
PP2A-C
140 -
100 -
p-PP2A-C(Y307)
p-JNK
80 -
UROtsa (BBN 6M)
JNK1/2
60 -
p-c-Jun Ser73
p-JNK
RhoGDIβ
54 kDa
50 -
β-Actin
40 -
30 -

## Slide 52
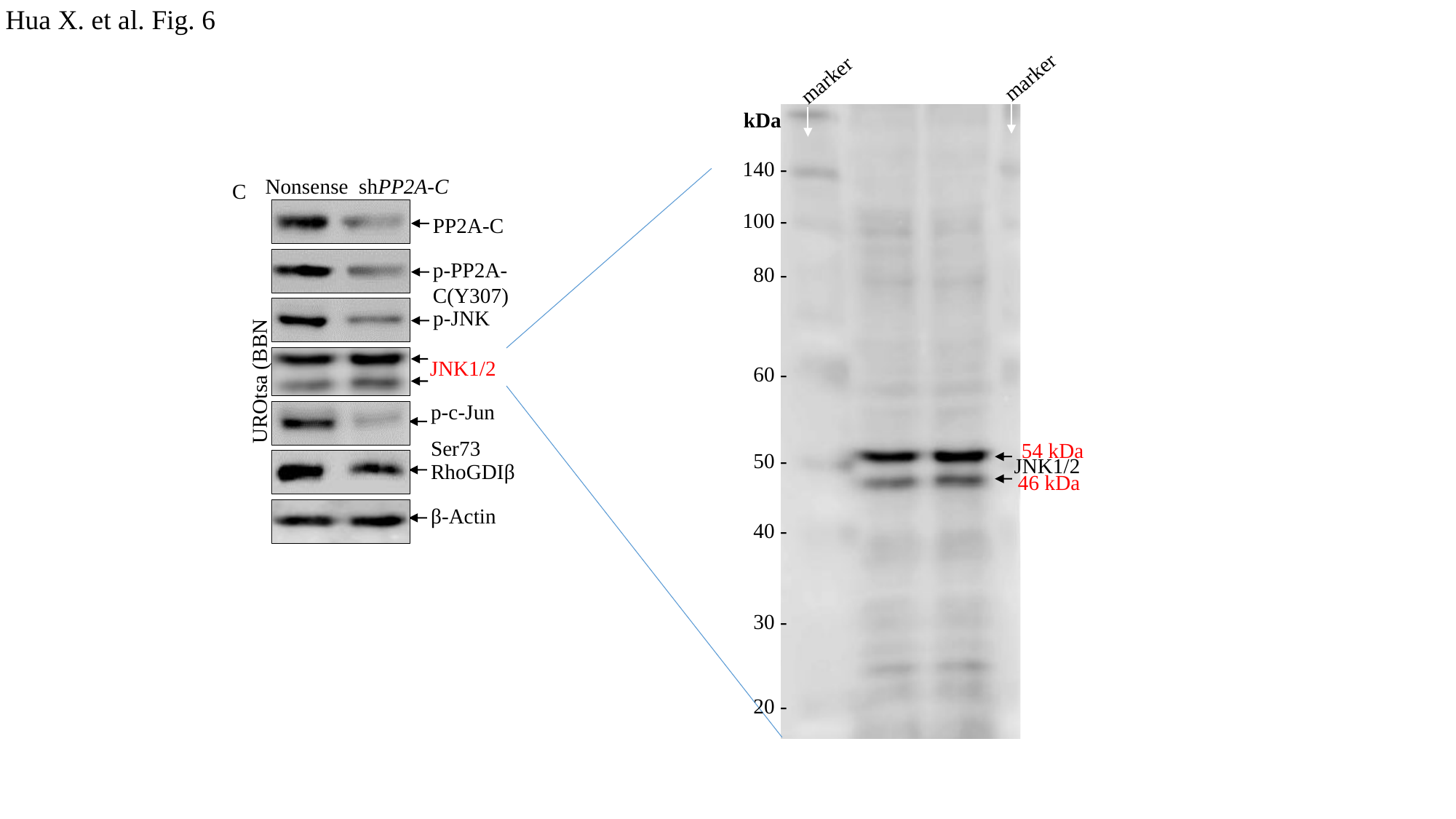

Hua X. et al. Fig. 6
marker
marker
kDa
140 -
Nonsense shPP2A-C
C
PP2A-C
100 -
p-PP2A-C(Y307)
80 -
p-JNK
UROtsa (BBN 6M)
JNK1/2
60 -
p-c-Jun Ser73
54 kDa
JNK1/2
50 -
RhoGDIβ
46 kDa
β-Actin
40 -
30 -
20 -

## Slide 53
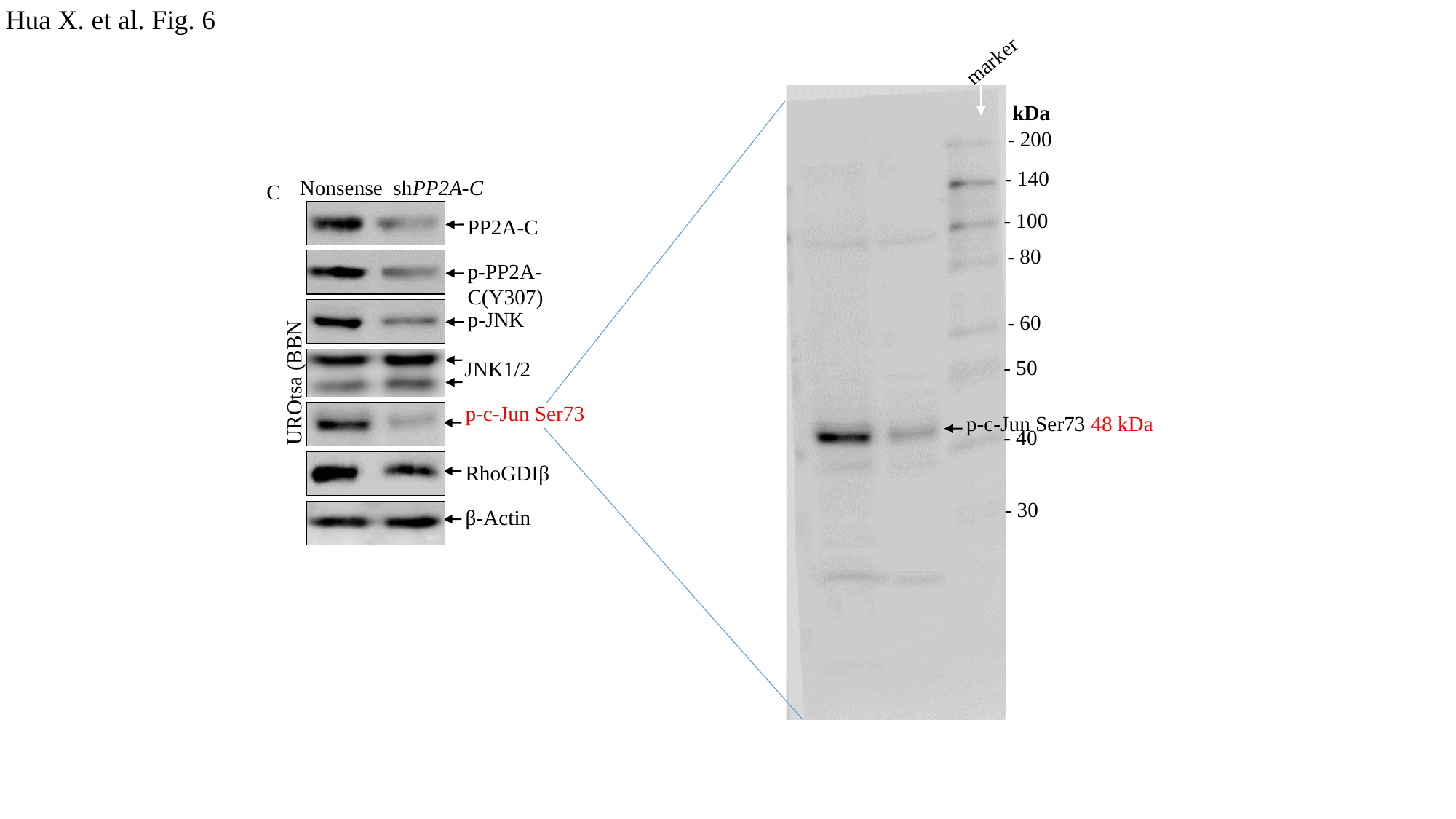

Hua X. et al. Fig. 6
marker
kDa
- 200
- 140
Nonsense shPP2A-C
C
PP2A-C
- 100
- 80
p-PP2A-C(Y307)
p-JNK
- 60
UROtsa (BBN 6M)
JNK1/2
- 50
p-c-Jun Ser73
48 kDa
p-c-Jun Ser73
- 40
RhoGDIβ
- 30
β-Actin

## Slide 54
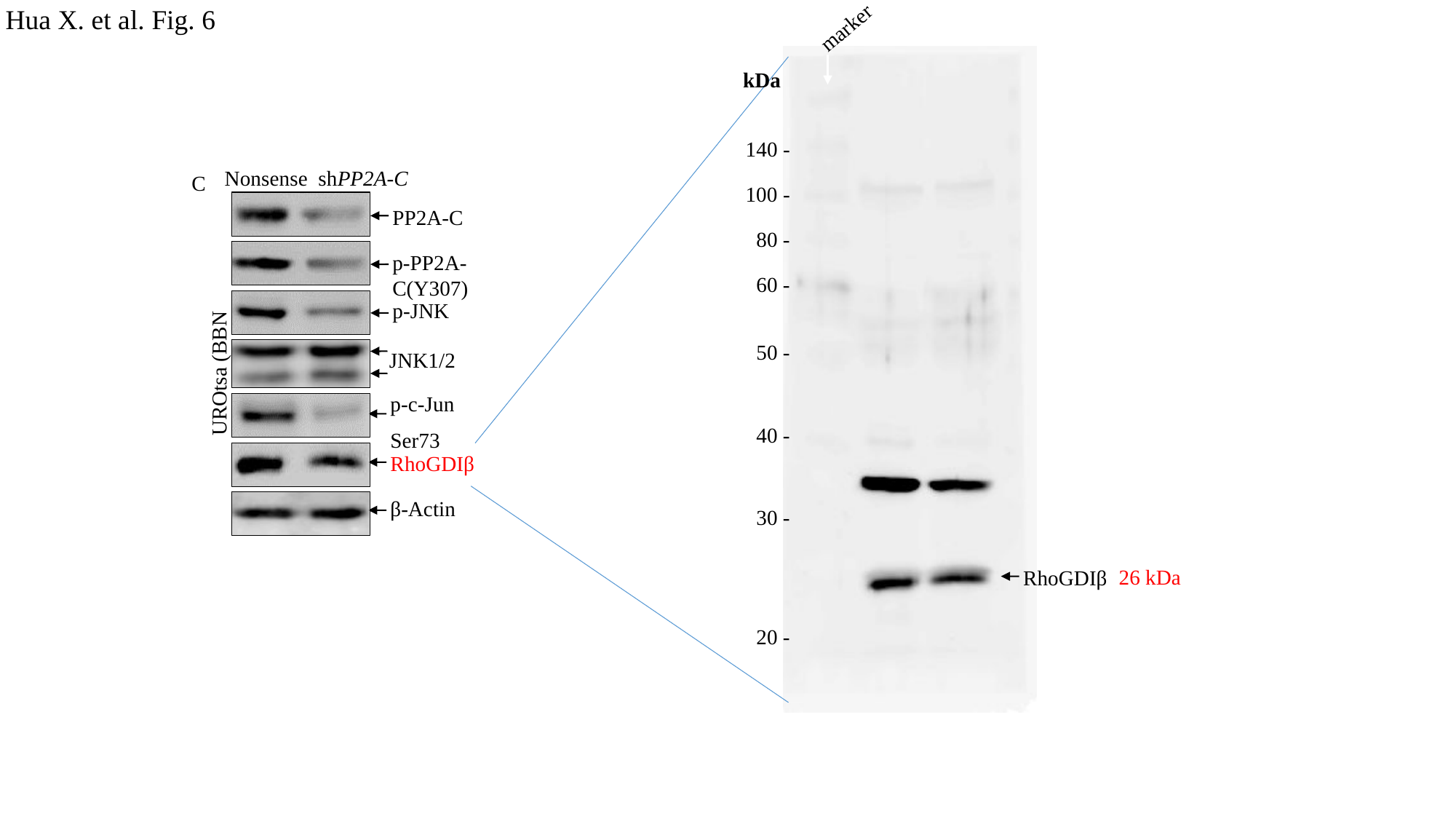

Hua X. et al. Fig. 6
marker
kDa
140 -
Nonsense shPP2A-C
C
100 -
PP2A-C
80 -
p-PP2A-C(Y307)
60 -
p-JNK
UROtsa (BBN 6M)
50 -
JNK1/2
p-c-Jun Ser73
40 -
RhoGDIβ
β-Actin
30 -
RhoGDIβ
26 kDa
20 -

## Slide 55
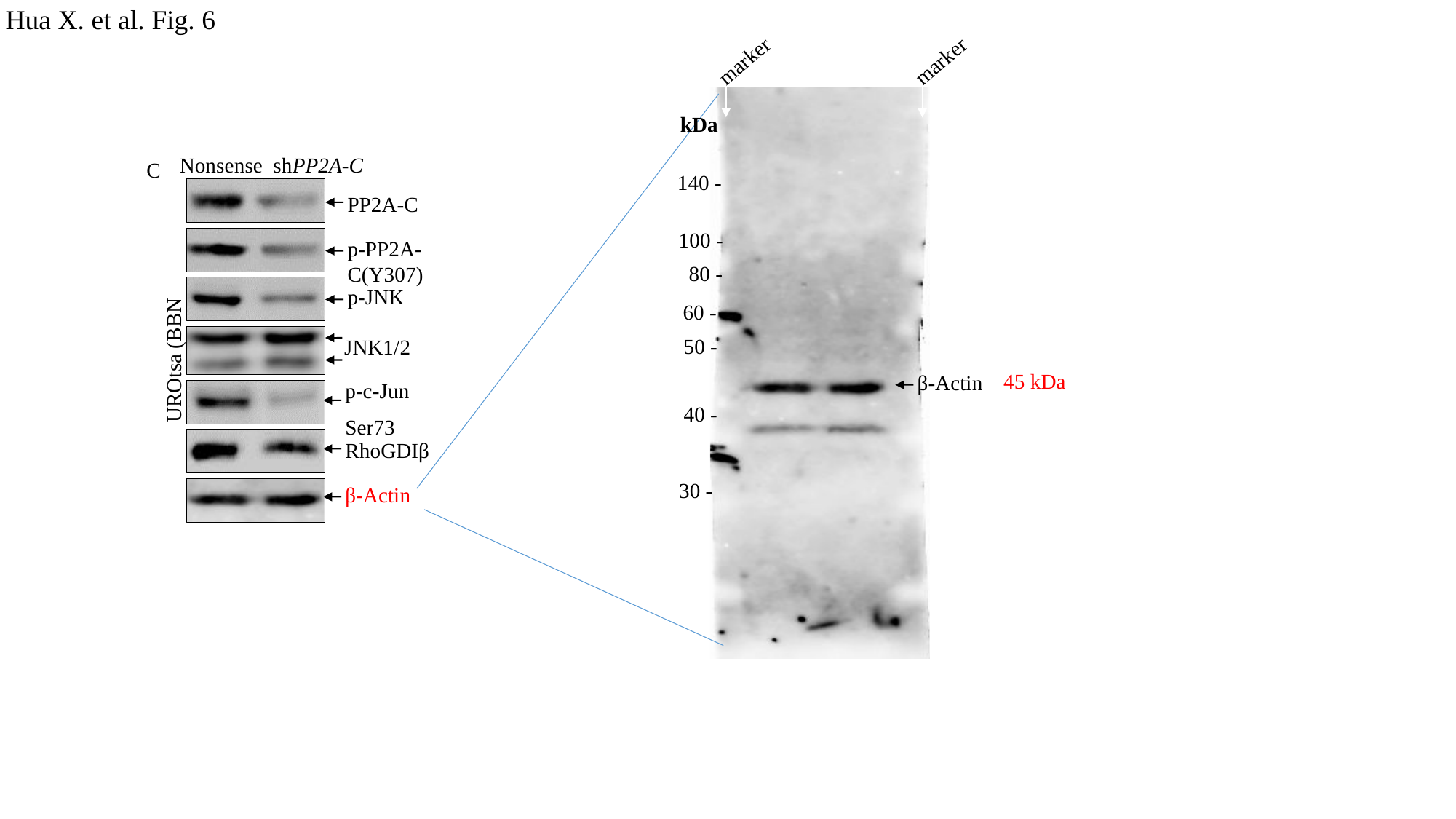

Hua X. et al. Fig. 6
marker
marker
kDa
Nonsense shPP2A-C
C
140 -
PP2A-C
100 -
p-PP2A-C(Y307)
80 -
p-JNK
60 -
UROtsa (BBN 6M)
JNK1/2
50 -
β-Actin
45 kDa
p-c-Jun Ser73
40 -
RhoGDIβ
β-Actin
30 -
